# Causal Associations between Dietary Habits and Chronic Pain: A Two-Sample Mendelian Randomization Study

**DOI:** 10.3390/nu15173709

**Published:** 2023-08-24

**Authors:** Ren Zhou, Lei Zhang, Yu Sun, Jia Yan, Hong Jiang

**Affiliations:** Department of Anesthesiology, The Ninth People’s Hospital of Shanghai, Jiao Tong University School of Medicine, Shanghai 200011, China; zhouren77@126.com (R.Z.); weiymzhl@126.com (L.Z.); dr_sunyu@163.com (Y.S.)

**Keywords:** chronic pain, dietary habits, Mendelian randomization, multisite chronic pain

## Abstract

Chronic pain is a prevalent and debilitating condition with significant impacts on individuals and society. While the role of diet in chronic pain is well-known, the relationship between special dietary choices and chronic pain remains unclear. This study investigates the causal associations between 20 dietary habits and chronic pain using a Mendelian randomization (MR) approach. Publicly available genome-wide association study data from the UK Biobank dataset were utilized for secondary analysis, and genetic instrumental variables strongly correlated with 20 different dietary habits were selected. Multisite chronic pain (MCP) scores were used as the primary outcome, with site-specific chronic pain (SSCP) including back pain, headache, knee pain, neck pain, and hip pain as secondary outcomes. The inverse-variance-weighted (IVW) method was the primary method used in the MR. The weighted median (WM) and Mendelian randomization pleiotropy residual sum and outlier test (MR-PRESSO) methods were used as sensitivity analyses. This study identified causal associations between specific dietary habits and chronic pain. A high intake of cheese, cereal, dried fruits, and fresh fruits was associated with lower MCP scores. Conversely, high alcohol, salt, pork, and poultry intakes were associated with higher MCP scores. Similar associations between special dietary habits and some types of SSCP, such as back and neck pain, were also observed. The findings were consistent across different statistical methods, and sensitivity analyses confirmed the reliability of the results. In conclusion, our study provides evidence of a causal relationship between various dietary habits and different types of chronic pain based on secondary analysis of the UK Biobank dataset. Adhering to an anti-inflammatory diet, including increased consumption of fruits and cereal while reducing salt and pork intake, may potentially alleviate chronic pain symptoms.

## 1. Introduction

Chronic pain (CP) is a complex condition that can arise from various causes and is characterized by persistent or recurring pain lasting for over three months [1,2]. It significantly impacts an individual’s physical and emotional well-being. Globally, approximately 30% of the population experiences CP, with an average of three reported pain locations [3]. In the United States, more than 50 million adults (20.5%) suffer from pain on most days [4]. The burden of CP on society and health systems continues to increase, especially with an aging population. While the exact mechanisms underlying CP are not fully understood, certain risk factors such as smoking, being overweight, and lifestyle choices have been identified [5,6].

The role of dietary habits in overall health and well-being is crucial. A balanced and nutritious diet has been associated with numerous health benefits, including weight management, disease prevention, and improved immune function [7,8]. Conversely, poor dietary choices characterized by excessive consumption of processed foods, added salts, and unhealthy fats have been linked to an increased risk of chronic conditions such as obesity, diabetes, cardiovascular diseases, and certain types of cancer [9,10,11,12]. Moreover, studies have suggested that an unhealthy diet may contribute to systemic inflammation, oxidative stress [13], and impaired gut health [14], all of which are implicated in the development and maintenance of chronic pain [15,16]. Therefore, investigating the relationship between dietary habits and CP is essential for a comprehensive understanding of how food choices impact overall health outcomes.

Dietary behavior and intake have the potential to influence the occurrence, maintenance, and perception of chronic pain. Unhealthy dietary behavior, often associated with overweight and obesity, is a primary contributing factor to adverse nutritional status in chronic pain patients [17,18]. Furthermore, studies have explored the connection between specific nutrients and CP, such as the correlation between chronic lower back pain or rheumatoid arthritis and diminished intake of vitamin D [19], alkaline minerals [20], and omega-3 polyunsaturated fatty acids [21]. Certain dietary habits, such as the consumption of dried fruits, have also been causally associated with chronic pain [22]. However, systematic studies focusing specifically on the examination of dietary habits in the context of CP are lacking.

In this study, we used Mendelian randomization to assess the effects of 20 dietary habits on chronic pain, including multi-site chronic pain (MCP), chronic back pain (BP), chronic headache (Headache), chronic knee pain (KP), chronic neck/shoulder pain (NP), and chronic hip pain (HP). Our aim is to provide insights into the role of dietary habits in CP prevention and guide recommendations regarding food source quality.

## 2. Method

### 2.1. Study Design

**Figure 1:**
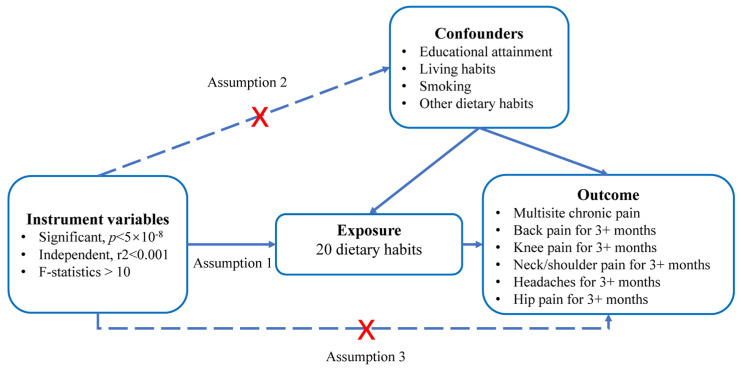
A directed acyclic graph is used to illustrate the hypothetical impact of dietary habits on chronic pain. The presence of a dotted line indicates a potential direct causal relationship or pleiotropic effect between the exposure and the outcome.

**Figure 2:**
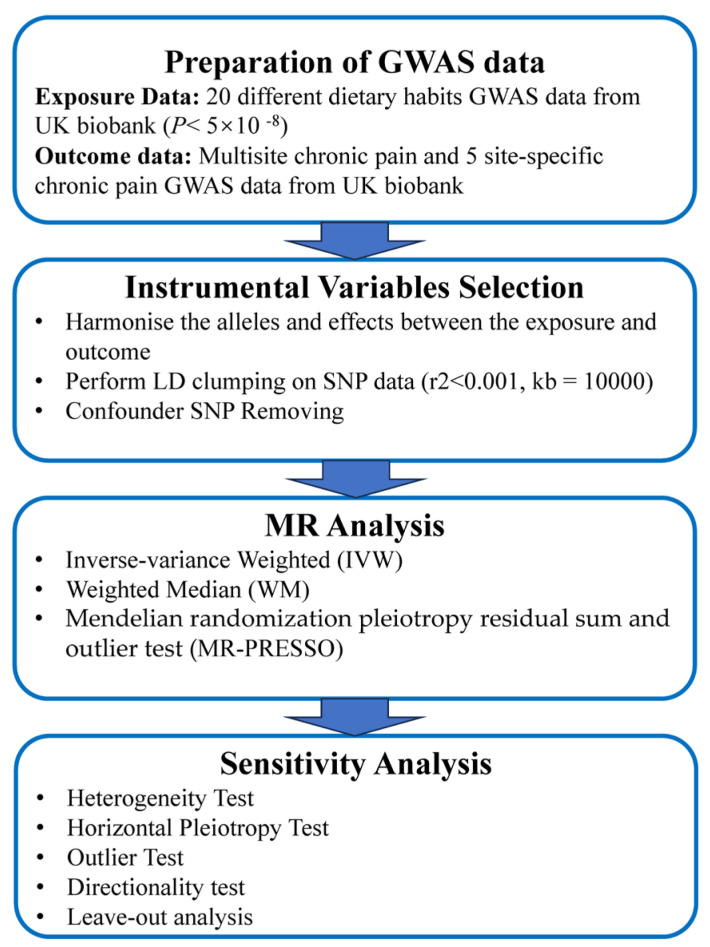
Flowchart of the MR analysis.

This study is a secondary analysis of the UK biobank dataset. We used a two-sample Mendelian randomization (MR) approach to investigate the causal relationships between dietary habits and CP. Figure 1 provides a schematic overview of the study design and data sources. Figure 2 provides a summary experimental protocol of our study. We exclusively used publicly available genome-wide association study (GWAS) summary statistics, eliminating the need for additional ethical approval or informed consent.

### 2.2. Exposure Data

In this study, we considered 20 different dietary habits as exposures based on previous studies [23,24], including beef intake, bread intake, cereal intake, cheese intake, coffee intake, cooked vegetable intake, dried fruit intake, fresh fruit intake, lamb/mutton intake, non-oily fish intake, oily fish intake, pork intake, poultry intake, processed meat intake, salad/raw vegetable intake, salt added to food, water intake, hot drink temperature, tea intake, and alcohol intake frequency. We obtained summary data from the GWAS conducted by the UK Biobank, a large prospective study comprising approximately 500,000 participants aged between 38 years and 73 years who provided their genetic and phenotypic information and were recruited between the years 2006 and 2010 from across the UK [25]. A full description of the study design, participants, and quality control (QC) methods has previously been described in detail [26]. The data for each dietary pattern consisted of integer variables, such as, on average, the number of cups of coffee per day, or categorical variables, such as how often one eats poultry. Unreasonable responses were excluded during data submission. The dietary habit assessment questions and specific definition of units (e.g., one tablespoon or one cup) used in our study can be found in the publicly available information on the UK Biobank website (http://biobank.ctsu.ox.ac.uk/crystal/label.cgi?id=100052, accessed on 10 August 2023, https://biobank.ctsu.ox.ac.uk/crystal/field.cgi?id=1558, accessed on 10 August 2023). The content of the specific questionnaires can be seen in Table S1. Summary GWAS data for dietary habits were collected from OpenGWAS, conducted by Elsworth et al., at MRC-IEU, and are maintained by MRC-IEU. Information on the collection, cleaning, and analysis of these raw GWAS data can be found in the original documentation at MRC-IEU.

### 2.3. Outcome Data

Our primary outcome was MCP, for which we obtained GWAS data from a large study conducted by Johnston et al. [27] involving 387,649 European individuals. MCP is defined as self-reported pain lasting at least three months in seven distinct bodily regions (head, face, neck/shoulder, back, stomach/abdomen, hip, and knee).

Secondary outcomes included specific site-specific chronic pain (SSCP) lasting more than three months. Summary GWAS data for SSCP were collected from OpenGWAS, by Elsworth et al., at MRC-IEU, and maintained by MRC-IEU. Information on the collection, cleaning, and analysis of this raw GWAS data can be found in the original documentation at MRC-IEU. QC files are also included in the documentation. The GWAS data related to headache, chronic hip pain, chronic neck/shoulder pain, chronic back pain, and chronic knee pain were included in our study. The CP assessment questions used in our study can be found in publicly available information from the UK Biobank website (https://biobank.ctsu.ox.ac.uk/crystal/label.cgi?id=154, accessed on 10 August 2023).

### 2.4. Selection of Instrumental Variables

To explore the causal impact of dietary habits on chronic pain (CP), we employed a two-sample MR design (see Figure 1). We established five criteria to screen single-nucleotide polymorphisms (SNPs) used as instrumental variables:

(a) We selected SNPs significantly correlated with dietary habits (*p* < 5 × 10^−8^), ensuring their independence from each other (r2 < 0.001) within a clumping distance of 10,000 kb;
(b) SNPs associated with the outcomes of interest (*p* < 5 × 10^−8^) were excluded from the instrumental variables;
(c) SNPs associated with potential confounding factors, such as educational attainment, living habits (e.g., usual walking pace, time spent watching television), smoking, and other dietary habits, were excluded from all analyses. The relevant information was obtained from the PhenoScanner database V2 (http://www.phenoscanner.medschl.cam.ac.uk/, accessed on 10 June 2023);
(d) To ensure a strong correlation between instrumental variables and exposure factors, we verified that the F-statistic of each SNP was larger than 10 using the formula F = β^2^/SE_β_^2^;
(e) Palindromic SNPs with intermediate allele frequencies were excluded from the analysis to maintain consistency between the effects of SNPs on exposures and outcomes.

Supplementary Tables S2–S5 provide detailed information on the selected SNPs.

### 2.5. Statistical Analysis

All statistical analyses were performed using R version 4.0.5 (R Foundation for Statistical Computing, Vienna, Austria) and using the TwoSampleMR and MRPRESSO R packages. When SNPs were unavailable in the outcome summary data, we used proxy SNPs with an r2 ≥ 0.8 in the highest linkage disequilibrium. We harmonized the exposure and outcome data and aligned the effect allele in the exposure and outcome GWAS using the TwoSampleMR package. 

The primary Mendelian randomization (MR) analysis relied on the inverse-variance-weighted (IVW) method. This approach employs a meta-analysis technique to combine the Wald ratios of individual SNPs. It assumes that instrumental variables (IVs) solely affect outcomes through specific exposures. In the absence of horizontal pleiotropy, the IVW method provides reliable results. The IVW method is commonly employed as a major method in MR studies. To ensure the robustness of our findings, we conducted several complementary analyses using the weighted median (WM) method and Mendelian randomization pleiotropy residual sum and outlier test (MR-PRESSO) methods [28] in subsequent sensitivity analyses. The IVW method only gives consistent estimates if all genetic variants are included. Additionally, the weighted median requires 50% of the weight to come from valid IVs. The MR-PRESSO analysis detects and attempts to reduce horizontal pleiotropy by removing significant outliers. Data are presented as β, standard errors (SE) of β, and *p*-values. A positive β means that an increased intake of a certain food is positively associated with pain, while a negative β means that increased intake of certain food is negatively associated with pain. In our study, we only considered a dietary habit to be associated with a specific CP when significant results were obtained for IVW and the results for WM and MR-PRESSO showed the same trend.

Heterogeneity in MR analysis refers to inconsistencies in the estimates derived from different instrumental variables, reflecting the compatibility of the instrumental variables with the causal inference being made. We calculated the Cochran Q statistic to quantify the heterogeneity of causal estimates generated from both the IVW and MR-Egger regression methods. A *p*-level > 0.05 means there is no significant heterogeneity detected in this analysis. The Mendelian randomization pleiotropy residual sum and outlier test (MR-PRESSO) [28] was also used to detect horizontal pleiotropic outliers in the multi-instrument MR study and to correct for horizontal pleiotropy via outlier removal. 

We used the Steiger directionality test to confirm the causal direction between dietary habits and CP; a *p*-level <0.05 meant the causal direction was correct in this analysis. To validate the reliability of the estimates, we performed a leave-one-out sensitivity analysis by recalculating the overall effect size and removing each SNP one at a time until reaching significant results for the primary outcome. 

For our primary outcome, we adjusted the *p*-values using the false detection rate (FDR) [29]. We considered associations with an FDR of less than 0.05 as significant. However, for the secondary outcome, we considered both the raw *p*-value and the FDR in our analysis. 

## 3. Results

### 3.1. Genetic Instruments for 20 Dietary Habits

Table 1 provides comprehensive information about each participating GWAS study. The analyses included a total of 20 different dietary habits as exposures. The number of single nucleotide polymorphisms (SNPs) considered for each dietary habit varied between 3 and 87. Supplementary Tables S2–S5 contain detailed information regarding the instrumental variables (IVs) employed for these 20 dietary habits. We assessed the impact of various dietary exposures on the outcome, and, in all cases, the F statistics of the identified SNPs surpassed the empirical threshold of 10, ranging from 29.74 to 811.86. This finding suggests that the obtained results are less susceptible to deviations caused by weak IVs.

### 3.2. Causal Effects of Dietary Habits on CP

**Figure 3:**
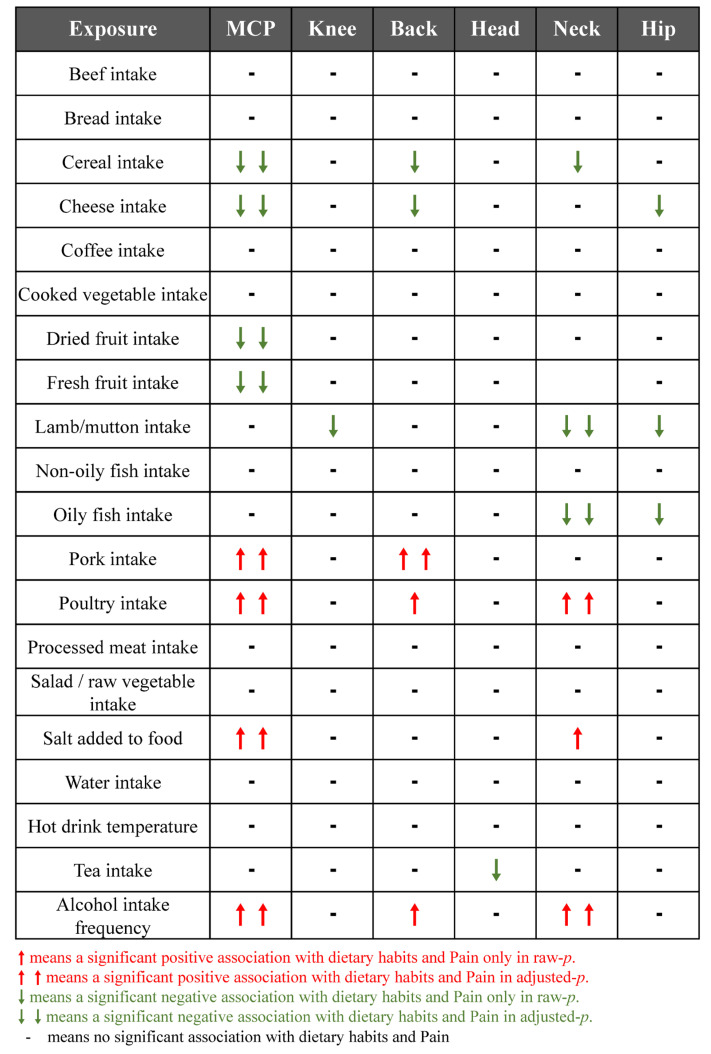
Summary results of casual association between dietary habits and chronic pain according to the inverse-variance-weighted method.

**Figure 4:**
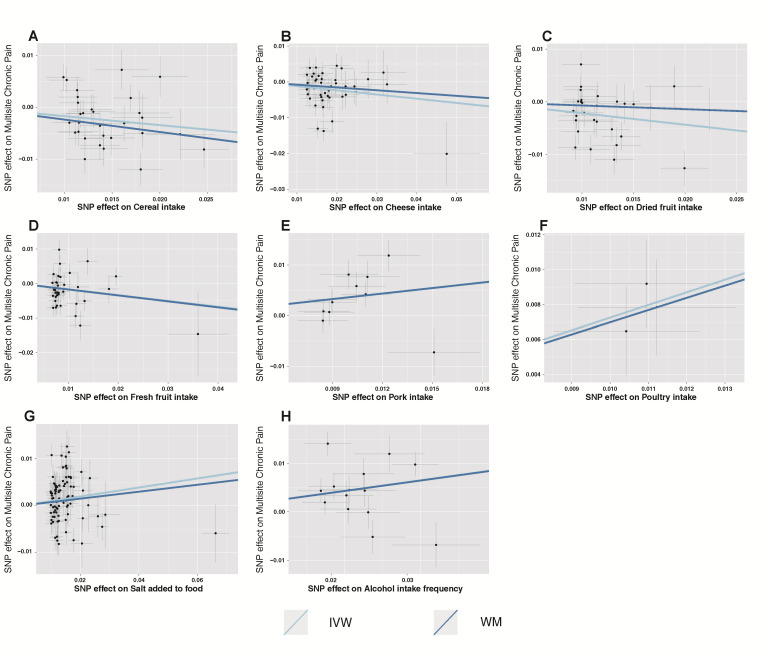
Scatter plots depicting the results of Mendelian randomization (MR) analyses investigating the association between dietary habits and multisite chronic pain. Each line in the plot represents a different MR method, and the slope of each line represents the estimated association between the two variables. (**A**) Scatter plot between cereal intake and multisite chronic pain; (**B**) Scatter plot between cheese intake and multisite chronic pain; (**C**) Scatter plot between dried fruit intake and multisite chronic pain; (**D**) Scatter plot between fresh fruit intake and multisite chronic pain; (**E**) Scatter plot between pork intake and multisite chronic pain; (**F**) Scatter plot between poultry intake and multisite chronic pain; (**G**) Scatter plot between salt added in food and multisite chronic pain; (**H**) Scatter plot between alcohol intake frequency and multisite chronic pain. IVW, inverse-variance-weighted; WM, weight median.

The summary of causal effects of dietary habits on different types of CP are shown in Figure 3. Therein, 8 causal associations between 20 dietary habits and MCP were identified based on the IVW method with adjusted *p*-values (Table 2). Among them, cheese intake (β = −0.12, SE_β_ = 0.04, *p*_Raw_ = 0.002, *p*_Adjusted_ = 0.008), cereal intake (β = −0.17, SE_β_ = 0.06, *p*_Raw_ = 0.004, *p*_Adjusted_ = 0.015), and high intake of dried fruit (β = −0.22, SE_β_ = 0.07, *p*_Raw_ = 0.001, *p*_Adjusted_ = 0.008) and fresh fruit (β = −0.17, SE_β_ = 0.07, *p*_Raw_ = 0.017, *p*_Adjusted_ = 0.041) reduced MCP scores. In contrast, high alcohol intake (β = 0.22, SE_β_ = 0.07, *P*_Raw_ = 0.001, *P*_Adjusted_ = 0.008), salt intake (β = 0.10, SE_β_ = 0.03, *p*_Raw_ = 0.006, *p*_Adjusted_ = 0.016), pork intake (β = 0.36, SE_β_ = 0.12, *p*_Raw_ = 0.003, *p*_Adjusted_ = 0.013), and poultry intake (β = 0.73, SE_β_ = 0.14, *p*_Raw_ < 0.001, *p*_Adjusted_ < 0.001) increased MCP scores. Further, our study found an association between dietary habits with different types of CP. Six dietary habits were associated with NP and four of them were still significant after adjusting the *p*-value (Table 3) with the IVW method. Five dietary habits were associated with BP and one was still significant after adjusting the *p*-value (Table 4) with the IVW method. Based on the IVW method, HP, KP, and headaches were associated with three, one, and one dietary habits, respectively. However, none of these was still significant after the adjusted *p*-value was applied (Supplementary Tables S6–S8). The results of the WM method and PRESSO method mostly showed a similar trend with those of the IVW method, though some *p*-values were slightly above 0.05 (Supplementary Tables S9–S20). We also constructed scatter plots to visualize the main results (Figure 4).

There was no evidence for potential pleiotropy in our study. However, a widespread heterogeneity effect was shown in our study. Thus, we used the random effect IVW as the major analysis method. For those dietary habits that did not show a significant heterogeneity effect in MR analysis, the fixed effect IVW was used. The MR-PRESSO global test showed no significant outliers in some results; however, all those results remained the same after excluding the outlier SNPs (Supplementary Tables S21–S26). The Steiger directionality test confirmed all the causal directions of dietary habits and CP (Supplementary Tables S21–S26).

**Figure 5:**
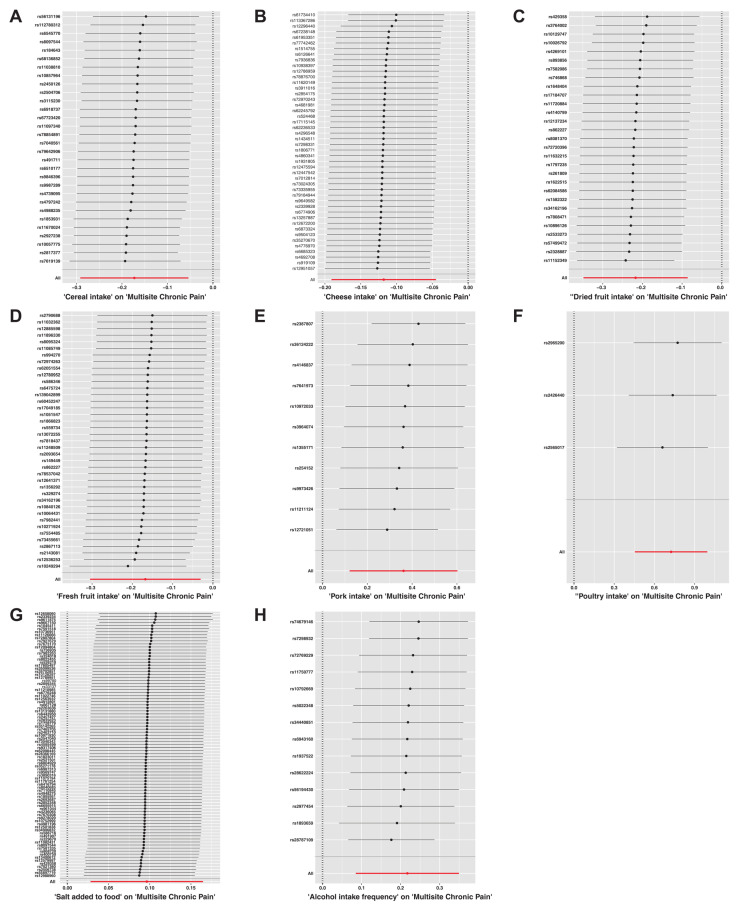
The results of a leave-one-out analysis on Mendelian randomization (MR). Each black line in the figure corresponds to the outcome of the MR analysis when one single nucleotide polymorphism (SNP) is removed from the analysis, while the remaining SNPs are used on the left. (**A**) Leave-one-out analysis between cereal intake and multisite chronic pain; (**B**) Leave-one-out analysis between cheese intake and multisite chronic pain; (**C**) Leave-one-out analysis between dried fruit intake and multisite chronic pain; (**D**) Leave-one-out analysis between fresh fruit intake and multisite chronic pain; (**E**) Leave-one-out analysis between pork intake and multisite chronic pain; (**F**) Leave-one-out analysis between poultry intake and multisite chronic pain; (**G**) Leave-one-out analysis between salt added in food and multisite chronic pain; (**H**) Leave-one-out analysis between alcohol intake frequency and multisite chronic pain. Red line reference the overall effect.

In our sensitivity analysis of the primary outcome, we conducted a leave-one-out analysis, excluding one significant SNP each time. For all the significant associations with MCP, the leave-one out estimates were also statistically significant (Figure 5). Funnel plots also proved the credibility of the results of the IVW method (Figure S1).

## 4. Discussion

In this study, we conducted a secondary analysis of the UK biobank dataset to investigate the impact of 20 dietary habits on chronic pain, including multi-site pain (MCP), back pain (BP), neck pain (NP), headache, hip pain (HP), and knee pain (KP). The results of our study revealed causal associations between various dietary habits and different types of chronic pain. For the primary outcome of MCP, we identified that cheese intake, cereal intake, and high consumption of dried and fresh fruit were associated with lower MCP scores. Conversely, high alcohol intake, salt intake, pork intake, and poultry intake were associated with higher MCP scores. Additionally, we found some correlations between dietary habits and other types of chronic pain other than headache.

Our main findings suggest that an inflammatory diet plays a role in chronic pain. Previous studies have extensively demonstrated the relationship between dietary intake and chronic inflammation [30,31]. Certain foods, such as vegetables and fruits, are considered anti-inflammatory, while others, including processed meats and trans-fatty acids, are known to promote inflammation and are referred to as pro-inflammatory foods. In our study, we observed a causal association between lower cereal and fruit intake and higher MCP scores. Both cereal and fruit are recognized as anti-inflammatory foods based on previous research [32,33,34]. On the other hand, high intakes of alcohol and salt, which showed a significant positive association with MCP scores in our study, are widely known to induce inflammatory responses in the body [35,36,37]. Some studies have also linked pork and poultry intake in the context of a high-fat diet to organismal inflammation [38]. While the association between chronic pain and many chronic inflammatory conditions is well-established [15,39], it is important to note that certain factors associated with dietary inflammation, such as processed meat intake, had a lesser impact on chronic pain [39]. Further research is needed to understand whether chronic inflammation is the sole mediator of chronic pain related to dietary habits.

Interestingly, our study revealed a negative association between cheese intake and MCP. Dairy fats, which are rich in saturated fatty acids, have been traditionally believed to have adverse effects on the body by raising LDL cholesterol levels. However, recent studies have suggested that certain types of dairy fat intake, such as cheese, may have a protective effect against the development of certain diseases such as heart disease [40]. In our study, we found an inverse causal association between cheese intake and MCP scores, indicating a potential protective effect of cheese consumption on MCP. In addition to saturated fatty acids, cheese is a good source of protein, calcium, and vitamin D [41], which are essential for maintaining bone and muscle health [42]. This is particularly relevant for individuals with chronic pain, as musculoskeletal issues often contribute to their discomfort.

Our study also demonstrated a positive causal association between pork and poultry intake and MCP scores. Pork and poultry may contain higher levels of pro-inflammatory compounds, which can contribute to increased inflammation in the body. Previous studies have suggested that red meat intake can contribute to the development of chronic inflammation in the body [43], and, although there is less research on poultry intake and inflammation, one study also found that high intake of chicken and pork proteins aggravated high-fat-diet-induced inflammation [38]. Chronic pain conditions often involve an underlying inflammatory response, and consuming foods that promote inflammation can worsen pain symptoms and increase discomfort. Interestingly, our study did not find a strong association between beef and lamb intake and CP, and lamb intake even showed a protective effect on certain types of chronic pain. The differential effects of different types of meat intake on health outcomes have also been observed in previous studies. For instance, one study found a beneficial effect of beef intake on depression, while pork and poultry did not show a similar effect [6]. Another study highlighted the potential negative impact of high chicken or pork intake in a high-fat diet on neuronal integrity and neurodegenerative disorders [38]. It is essential for future studies to further differentiate between various types of meat intake when assessing their health effects.

It is important to acknowledge that our study shares common limitations with Mendelian randomization studies. Assumptions may be violated due to horizontal pleiotropy, where a genetic variant affecting the outcome through a different pathway from the investigated exposure could introduce biased estimates. However, we conducted several sensitivity analyses, including MR-PRESSO, an intercept test in MR-Egger regression, a heterogeneity test, and leave-one-out analysis, which consistently supported our results. Another limitation is that the analyzed GWASs primarily focused on individuals of European ancestry, which may limit the generalizability of our findings to other ethnicities. Furthermore, due to the lack of detailed data in the original studies, additional non-linear regression and subgroup analyses were not feasible.

## 5. Conclusions

In conclusion, our study provides evidence of a causal relationship between various dietary habits and different types of chronic pain. Adhering to an anti-inflammatory diet, including increased consumption of fruit and cereal while reducing salt and pork intake, may potentially alleviate chronic pain symptoms.

## Figures and Tables

**Table 1:** Detailed information on the GWAS datasets used in this MR study.

ID or PMID	Food Types	Trait	Sample Size	Case	Control	SNPs (N)	Consortium (Author)	Population	Category	Year
ukb-b-2862	Meat and Poultry	Beef intake	461,053	NA	NA	9,851,867	MRC-IEU (Elsworth et al.)	European	Categorical (single)	2018
ukb-b-11348	Bread	Bread intake	452,236	NA	NA	9,851,867	MRC-IEU (Elsworth et al.)	European	Integer, slices/week	2018
ukb-b-15926	Grains, Nuts, and Seeds	Cereal intake	441,640	NA	NA	9,851,867	MRC-IEU (Elsworth et al.)	European	Integer, bowls/week	2018
ukb-b-1489	Dairy Products	Cheese intake	451,486	NA	NA	9,851,867	MRC-IEU (Elsworth et al.)	European	Categorical (single)	2018
ukb-b-5237	Drinks	Coffee intake	428,860	NA	NA	9,851,867	MRC-IEU (Elsworth et al.)	European	Integer, cups/day	2018
ukb-b-8089	Vegetable	Cooked vegetable intake	448,651	NA	NA	9,851,867	MRC-IEU (Elsworth et al.)	European	Integer, tablespoons/day	2018
ukb-b-16576	Fruit	Dried fruit intake	421,764	NA	NA	9,851,867	MRC-IEU (Elsworth et al.)	European	Integer, pieces/day	2018
ukb-b-3881	Fruit	Fresh fruit intake	446,462	NA	NA	9,851,867	MRC-IEU (Elsworth et al.)	European	Integer, pieces/day	2018
ukb-b-14179	Meat and Poultry	Lamb/mutton intake	460,006	NA	NA	9,851,867	MRC-IEU (Elsworth et al.)	European	Categorical (single)	2018
ukb-b-17627	Seafood	Non-oily fish intake	460,880	NA	NA	9,851,867	MRC-IEU (Elsworth et al.)	European	Categorical (single)	2018
ukb-b-2209	Seafood	Oily fish intake	460,443	NA	NA	9,851,867	MRC-IEU (Elsworth et al.)	European	Categorical (single)	2018
ukb-b-5640	Meat and Poultry	Pork intake	460,162	NA	NA	9,851,867	MRC-IEU (Elsworth et al.)	European	Categorical (single)	2018
ukb-b-8006	Meat and Poultry	Poultry intake	461,900	NA	NA	9,851,867	MRC-IEU (Elsworth et al.)	European	Categorical (single)	2018
ukb-b-6324	Meat and Poultry	Processed meat intake	461,981	NA	NA	9,851,867	MRC-IEU (Elsworth et al.)	European	Categorical (single)	2018
ukb-b-1996	Vegetable	Salad/raw vegetable intake	435,435	NA	NA	9,851,867	MRC-IEU (Elsworth et al.)	European	Integer, tablespoons/day	2018
ukb-b-8121	Food additive	Salt added to food	462,630	NA	NA	9,851,867	MRC-IEU (Elsworth et al.)	European	Categorical (single)	2018
ukb-b-14898	Drinks	Water intake	427,588	NA	NA	9,851,867	MRC-IEU (Elsworth et al.)	European	Integer, glasses/day	2018
ukb-b-14203	-	Hot drink temperature	457,873	NA	NA	9,851,867	MRC-IEU (Elsworth et al.)	European	Categorical (single)	2018
ukb-b-6066	Drinks	Tea intake	447,485	NA	NA	9,851,867	MRC-IEU (Elsworth et al.)	European	Integer, cups/day	2018
ukb-b-5779	Drinks	Alcohol intake frequency.	462,346	NA	NA	9,851,867	MRC-IEU (Elsworth et al.)	European	Categorical (single)	2018
31194737	-	Multisite chronic pain	387,649	NA	NA	9,926,106	Johnston et al. [27]	European	Categorical Ordered	2019
ukb-b-8463	-	Back pain for 3+ months	117,404	80,588	36,816	9,851,867	MRC-IEU (Elsworth et al.)	European	Binary	2018
ukb-b-8906	-	Knee pain for 3+ months	97,889	76,910	20,979	9,851,867	MRC-IEU (Elsworth et al.)	European	Binary	2018
ukb-b-16118	-	Neck/shoulder pain for 3+ months	105,396	72,887	32,509	9,851,867	MRC-IEU (Elsworth et al.)	European	Binary	2018
ukb-b-13092	-	Headache for 3+ months	91,269	41,719	49,550	9,851,867	MRC-IEU (Elsworth et al.)	European	Binary	2018
ukb-b-133	-	Hip pain for 3+ months	51,516	40,152	11,364	9,851,867	MRC-IEU (Elsworth et al.)	European	Binary	2018

**Table 2:** MR results of the IVW method for the association of dietary habits with multi-site chronic pain.

Exposure	Number of SNPs	β	SE_β_	*p*-Value	Adjusted *p*-Value
Beef intake	13	0.10	0.11	0.389	0.648
Bread intake	26	−0.01	0.07	0.882	0.936
Cereal intake	30	−0.17	0.06	**0.004**	**0.015**
Cheese intake	46	−0.12	0.04	**0.002**	**0.008**
Coffee intake	29	−0.04	0.06	0.554	0.791
Cooked vegetable intake	11	0.11	0.11	0.294	0.534
Dried fruit intake	29	−0.22	0.07	**0.001**	**0.008**
Fresh fruit intake	39	−0.17	0.07	**0.017**	**0.041**
Lamb/mutton intake	29	−0.06	0.08	0.479	0.737
Non-oily fish intake	6	−0.02	0.22	0.924	0.936
Oily fish intake	50	−0.06	0.05	0.214	0.428
Pork intake	11	0.36	0.12	**0.003**	**0.013**
Poultry intake	3	0.73	0.14	**<0.001**	**<0.001**
Processed meat intake	18	0.01	0.08	0.936	0.936
Salad/raw vegetable intake	14	−0.16	0.11	0.158	0.351
Salt added to food	87	0.10	0.03	**0.006**	**0.016**
Water intake	33	−0.01	0.06	0.807	0.936
Hot drink temperature	57	0.02	0.07	0.777	0.936
Tea intake	31	−0.03	0.07	0.685	0.914
Alcohol intake frequency	14	0.22	0.07	**0.001**	**0.008**

MR, Mendelian randomization; IVW, Inverse-variance-weighted. Bold indicated a *p* value less than 0.05.

**Table 3:** MR results of IVW method for association of dietary habits with chronic neck/shoulder pain.

Exposure	Number of SNPs	β	SE_β_	*p*-Value	Adjusted *p*-Value
Beef intake	13	0.08	0.05	0.09	0.301
Bread intake	27	−0.01	0.03	0.795	0.837
Cereal intake	30	−0.08	0.04	**0.037**	0.148
Cheese intake	46	−0.06	0.02	**0.007**	0.069
Coffee intake	29	−0.02	0.03	0.607	0.828
Cooked vegetable intake	11	0.06	0.06	0.34	0.617
Dried fruit intake	29	−0.05	0.03	0.14	0.354
Fresh fruit intake	39	−0.04	0.04	0.274	0.61
Lamb/mutton intake	29	0.01	0.04	0.749	0.837
Non-oily fish intake	6	0.11	0.08	0.142	0.354
Oily fish intake	50	−0.02	0.02	0.329	0.617
Pork intake	11	0.23	0.06	**<0.001**	**0.01**
Poultry intake	3	0.23	0.11	**0.037**	0.148
Processed meat intake	18	−0.02	0.04	0.662	0.828
Salad/raw vegetable intake	14	0.02	0.06	0.779	0.837
Salt added to food	87	0.01	0.02	0.647	0.828
Water intake	34	−0.02	0.03	0.55	0.828
Hot drink temperature	57	0	0.04	0.986	0.986
Tea intake	32	−0.02	0.02	0.449	0.748
Alcohol intake frequency	15	0.06	0.03	**0.023**	0.148

MR, Mendelian randomization; IVW, Inverse-variance-weighted. Bold indicated a *p* value less than 0.05.

**Table 4:** MR results of IVW method for association of dietary habits with chronic back pain.

Exposure	Number of SNPs	β	SE_β_	*p*-Value	Adjusted *p*-Value
Beef intake	13	0.08	0.05	0.09	0.301
Bread intake	27	−0.01	0.03	0.795	0.837
Cereal intake	30	−0.08	0.04	**0.037**	0.148
Cheese intake	46	−0.06	0.02	**0.007**	0.069
Coffee intake	29	−0.02	0.03	0.607	0.828
Cooked vegetable intake	11	0.06	0.06	0.34	0.617
Dried fruit intake	29	−0.05	0.03	0.14	0.354
Fresh fruit intake	39	−0.04	0.04	0.274	0.61
Lamb/mutton intake	29	0.01	0.04	0.749	0.837
Non-oily fish intake	6	0.11	0.08	0.142	0.354
Oily fish intake	50	−0.02	0.02	0.329	0.617
Pork intake	11	0.23	0.06	**<0.001**	**0.01**
Poultry intake	3	0.23	0.11	**0.037**	0.148
Processed meat intake	18	−0.02	0.04	0.662	0.828
Salad/raw vegetable intake	14	0.02	0.06	0.779	0.837
Salt added to food	87	0.01	0.02	0.647	0.828
Water intake	34	−0.02	0.03	0.55	0.828
Hot drink temperature	57	0	0.04	0.986	0.986
Tea intake	32	−0.02	0.02	0.449	0.748
Alcohol intake frequency	15	0.06	0.03	**0.023**	0.148

MR, Mendelian randomization; IVW, Inverse-variance-weighted. Bold indicated a *p* value less than 0.05.

## Data Availability

The data supporting the findings of this study are available in IEU open GWAS project websites (https://gwas.mrcieu.ac.uk/) and can also obtained from the corresponding author upon reasonable request.

## Supplementary Materials

The following supporting information can be downloaded at: https://www.mdpi.com/article/10.3390/nu15173709/s1, Figure S1: The figure displays the results of funnel plots in Mendelian randomization (MR). Table S1 shown summary of 20 dietary habits questionnaire; Table S2–S5 shown the details of SNPs select; Table S6–S8 shown the association between dietary habits and headache, chronic knee pain and chronic hip pain measured by IVW method; Table S9–S14 shown the association between dietary habits and chronic pain measured by weight median method; Table S15–S20 shown the association between dietary habits and chronic pain measured by weight median method; Table S21–S26 shown the results of Heterogeneity, Pleiotropy, and directionality test.
