# Supplementary material for: Causal Associations between Dietary Habits and Chronic Pain: A Two-Sample Mendelian Randomization Study
Source: Nutrients. 2023 Aug 24;15(17):3709. doi: 10.3390/nu15173709 (PMC10490345; doi:10.3390/nu15173709)
Supplement: Supplementary file 1 [file nutrients-15-03709-s001.zip › nutrients-2481613-supplementary.pdf]

Causal Associations Between Dietary Habits and Chronic Pain: A Two-Sample Mendelian Randomization Study

Ren Zhou, Lei Zhang, Yu Sun, Jia Yan*, and Hong Jiang*

Department of Anesthesiology, The Ninth People's Hospital of Shanghai, Jiao Tong University School of Medicine, Shanghai, 200011, PR China

*** Corresponding author**

Hong Jiang

The Ninth People's Hospital of Shanghai, Jiao Tong University School of Medicine No. 639 Zhizaoju Road, Shanghai, 200011, PR China.

Email: jianghongjiuyuan@163.com

Jia Yan

The Ninth People's Hospital of Shanghai, Jiao Tong University School of Medicine No. 639 Zhizaoju Road, Shanghai, 200011, PR China.

Email: mzkyanj@163.com

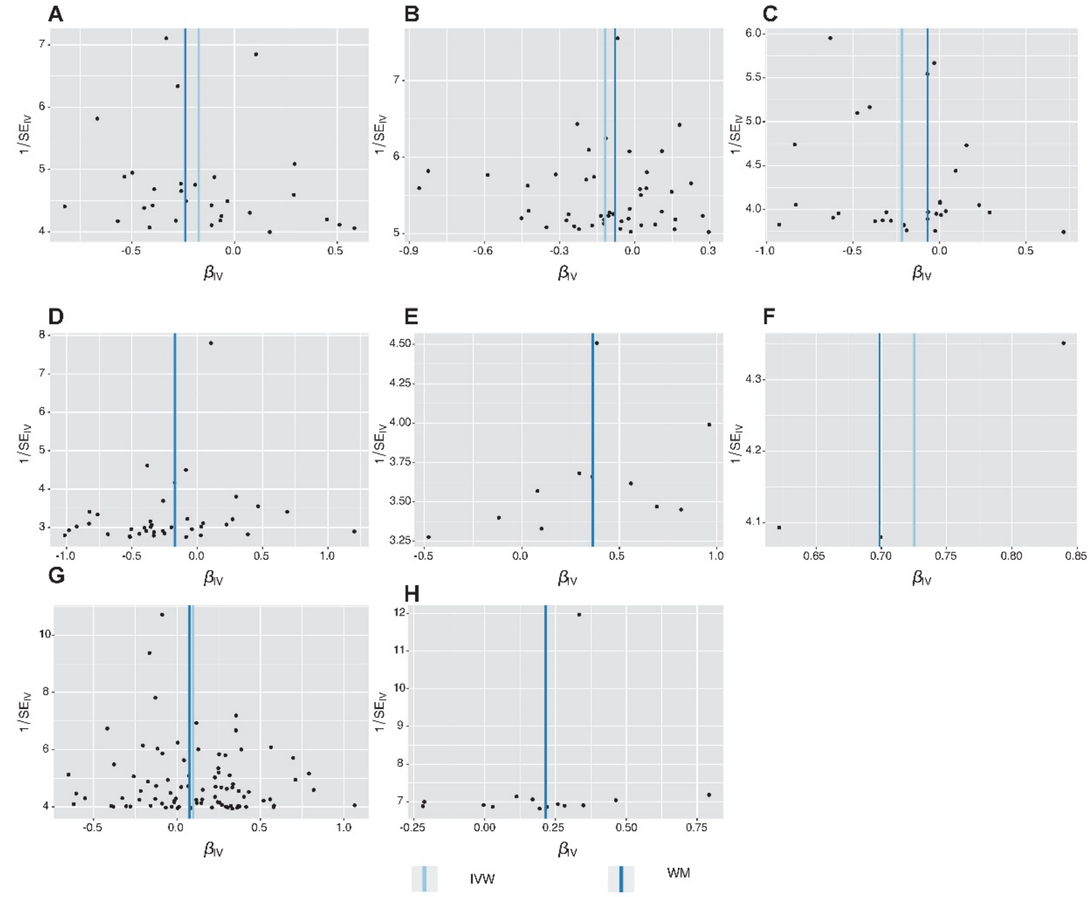

Figure S1: The figure displays the results of funnel plots in Mendelian randomization (MR). Funnel plot shows the estimates of precision (1/SE) and Wald ratios for each SNP. A: Funnel plot for cereal intake and multisite chronic pain; B: Funnel plot for cheese intake and multisite chronic pain; C: Funnel plot for dried fruits intake and multisite chronic pain; D: Funnel plot for fresh fruits intake and multisite chronic pain; E: Funnel plot for pork intake and multisite chronic pain; F: Funnel plot for poultry intake and multisite chronic pain; G: Funnel plot for salt added in food and multisite chronic pain; H: Funnel plot for alcohol intake frequency and multisite chronic pain. IVW, inverse-variance-weighted; WM, weight median.

Tables

Table S1 shown summary of 20 dietary habits questionnaire

Table S2-S5 shown the details of SNPs select.

Table S6-S8 shown the association between dietary habits and headache, chronic knee pain and chronic hip pain measured by IVW method.

Table S9-S14 shown the association between dietary habits and chronic pain measured by weight median method.

Table S15-S20 shown the association between dietary habits and chronic pain measured by weight median method.

Table S21-S26 shown the results of Heterogeneity, Pleiotropy, and directionality test.

Table S1 Summary of 20 dietary habits questionnaire

Trait	Category	Question	Options
Beef intake	Categorical (single)	How often do you eat beef? (Do not count processed meats)	Options: never, less than once a week, once a week, 2-4 times a week, 5-6 times a week, once or more daily, do not know, prefer not to answer
Bread intake	Integer, slices/week	How many slices of bread do you eat each WEEK?<br>For other types of bread:<br>- one bread roll = 2 slices<br>- one pitta bread = 2 slices	-
Cereal intake	Integer, bowls/week	How many bowls of cereal do you eat a WEEK?	-
Cheese intake	Categorical (single)	How often do you eat cheese? (Include cheese in pizzas, quiches, cheese sauce etc)	Options: never, less than once a week, once a week, 2-4 times a week, 5-6 times a week, once or more daily, do not know, prefer not to answer
Coffee intake	Integer, cups/day	How many cups of coffee do you drink each DAY? (Include decaffeinated coffee)	-
Cooked vegetable intake	Integer, tablespoons/ day	On average how many heaped tablespoons of COOKED vegetables would you eat per DAY? (Do not include potatoes; put '0' if you do not eat any)	-
Dried fruit intake	Integer, pieces/day	About how many pieces of DRIED fruit would you eat per DAY? (Count one prune, one dried apricot, 10 raisins as one piece; put '0' if you do not eat any)	-
Fresh fruit intake	Integer, pieces/day	About how many pieces of FRESH fruit would you eat per DAY? (Count one apple, one banana, 10 grapes etc as one piece; put '0' if you do not eat any)	-
Lamb/mutton intake	Categorical (single)	How often do you eat lamb/mutton? (Do not count processed meats)	Options: never, less than once a week, once a week, 2-4 times a week, 5-6 times a week, once or more daily, do not know, prefer not to answer
Oily fish intake	Categorical (single)	How often do you eat oily fish? (e.g. sardines, salmon, mackerel, herring)<br>Oily fish include: Salmon Anchovies, Trout Swordfish, Mackerel Bloater, Herring Cacha, Sardines Carp, Pilchards Hilsa, Kipper Jack fish, Eel Katla, Whitebait Orange roughy, Tuna (fresh only) Pangas, Sprats	Options: never, less than once a week, once a week, 2-4 times a week, 5-6 times a week, once or more daily, do not know, prefer not to answer
Non-oily fish intake	Categorical (single)	How often do you eat other types of fish? (e.g. cod, tinned tuna, haddock)	Options: never, less than once a week, once a week, 2-4 times a week, 5-6 times a week, once or more daily, do not know, prefer not to answer
Pork intake	Categorical (single)	How often do you eat pork? (Do not count processed meats such as bacon or ham)	Options: never, less than once a week, once a week, 2-4 times a week, 5-6 times a week, once or more daily, do not know, prefer not to answer
Poultry intake	Categorical (single)	How often do you eat chicken, turkey or other poultry? (Do not count processed meats)	Options: never, less than once a week, once a week, 2-4 times a week, 5-6 times a week, once or more daily, do not know, prefer not to answer

Processed meat intake	Categorical (single)	How often do you eat processed meats (such as bacon, ham, sausages, meat pies, kebabs, burgers, chicken nuggets)?	Options: never, less than once a week, once a week, 2-4 times a week, 5-6 times a week, once or more daily, do not know, prefer not to answer
Salad / raw vegetable intake	Integer, tablespoons/ day	On average how many heaped tablespoons of SALAD or RAW vegetables would you eat per DAY? (Include lettuce, tomato in sandwiches; put '0' if you do not eat any)	-
Salt added to food	Categorical (single)	Do you add salt to your food? (Do not include salt used in cooking)	Options: never/ rarely, sometimes, usually, always, prefer not to answer
Water intake	Integer, glasses/day	How many glasses of water do you drink each DAY?	-
Hot drink temperature	Categorical (single)	How do you like your hot drinks? (Such as coffee or tea)	Options: very hot, hot, warm, do not drink hot drinks, prefer not to answer
Tea intake	Integer, cups/day	How many cups of tea do you drink each DAY? (Include black and green tea)	-
Alcohol intake frequency	Categorical (single)	About how often do you drink alcohol?	Options: daily or almost daily, three or four times a week, once or twice a week, one to three times a month, special occasions only, never, prefer not to answer

Table S2:SNPs significantly correlated with dietary habits ($P < 5 \times 10^{-8}$) and independence from each other ($r^2 < 0.001$) within a clumping distance of 10,000 kb.

exposure	SNP	samplesize.exposure	beta.exposure	se.exposure	pval.exposure	effect_allele.exposure	other_allele.exposure	eaf.exposure	F_value
Beef intake || id:ukb-b-2862	rs11165829	461053	-0.0102	0.001779	9.80E-09	G	C	0.35997	32.88727317
Beef intake || id:ukb-b-2862	rs1105388	461053	-0.01138	0.001876	1.30E-09	T	C	0.300147	36.78231107
Beef intake || id:ukb-b-2862	rs10789340	461053	-0.01378	0.001769	6.80E-15	G	A	0.626712	60.65227778
Beef intake || id:ukb-b-2862	rs1470610	461053	-0.01222	0.002158	1.50E-08	C	G	0.196185	32.05033591
Beef intake || id:ukb-b-2862	rs62169335	461053	-0.00969	0.001735	2.40E-08	T	C	0.543241	31.16017894
Beef intake || id:ukb-b-2862	rs4676964	461053	0.013353	0.001724	9.60E-15	T	C	0.510605	59.96685299
Beef intake || id:ukb-b-2862	rs62396185	461053	-0.01484	0.001951	2.80E-14	C	G	0.260057	57.89211388
Beef intake || id:ukb-b-2862	rs7791463	461053	0.009548	0.001711	2.40E-08	A	G	0.534781	31.15691847
Beef intake || id:ukb-b-2862	rs79809011	461053	-0.02806	0.005085	3.40E-08	A	G	0.029465	30.45106577
Beef intake || id:ukb-b-2862	rs9407624	461053	-0.01376	0.001716	1.10E-15	A	T	0.488187	64.33126461
Beef intake || id:ukb-b-2862	rs10959890	461053	-0.01267	0.002096	1.50E-09	C	T	0.212297	36.51212522
Beef intake || id:ukb-b-2862	rs12247907	461053	0.009841	0.001711	8.90E-09	C	G	0.485752	33.0688003
Beef intake || id:ukb-b-2862	rs1421085	461053	-0.01212	0.001743	3.50E-12	C	T	0.403433	48.39152861
Beef intake || id:ukb-b-2862	rs784251	461053	-0.01034	0.001717	1.70E-09	T	C	0.477649	36.27765374
Beef intake || id:ukb-b-2862	rs429358	461053	-0.01485	0.002368	3.60E-10	C	T	0.154226	39.31868769
Beef intake || id:ukb-b-2862	rs11878917	461053	0.015019	0.002748	4.60E-08	A	G	0.109688	29.86711671
Beef intake || id:ukb-b-2862	rs132901	461053	0.013925	0.002093	2.90E-11	T	C	0.787662	44.27111051
Bread intake || id:ukb-b-11348	rs9662365	452236	0.012166	0.001989	9.60E-10	T	C	0.499481	37.40601951
Bread intake || id:ukb-b-11348	rs13023099	452236	-0.01147	0.002023	1.40E-08	A	C	0.571784	32.15521872
Bread intake || id:ukb-b-11348	rs6754311	452236	0.014135	0.002247	3.10E-10	C	T	0.264485	39.58860057
Bread intake || id:ukb-b-11348	rs4665972	452236	-0.01424	0.002043	3.20E-12	C	T	0.604541	48.53963829
Bread intake || id:ukb-b-11348	rs75287965	452236	-0.02475	0.004097	1.50E-09	A	G	0.062933	36.47836298
Bread intake || id:ukb-b-11348	rs13016665	452236	0.014754	0.002028	3.50E-13	A	C	0.423235	52.9155126
Bread intake || id:ukb-b-11348	rs1492988	452236	0.011545	0.002035	1.40E-08	G	C	0.599095	32.16824424
Bread intake || id:ukb-b-11348	rs9832088	452236	0.014729	0.001989	1.30E-13	A	T	0.521617	54.83556556
Bread intake || id:ukb-b-11348	rs9881332	452236	0.01137	0.002022	1.90E-08	G	C	0.581804	31.62463015
Bread intake || id:ukb-b-11348	rs1994315	452236	-0.01682	0.002144	4.30E-15	C	T	0.685162	61.5490717
Bread intake || id:ukb-b-11348	rs73802707	452236	-0.01593	0.002762	8.00E-09	T	C	0.153694	33.2715066
Bread intake || id:ukb-b-11348	rs2068650	452236	-0.01394	0.001999	3.10E-12	C	A	0.472074	48.60146191
Bread intake || id:ukb-b-11348	rs17083079	452236	0.030114	0.004679	1.20E-10	A	G	0.047423	41.41469758
Bread intake || id:ukb-b-11348	rs2517678	452236	0.013233	0.002086	2.20E-10	T	C	0.368395	40.24355561
Bread intake || id:ukb-b-11348	rs596878	452236	-0.01174	0.002011	5.30E-09	C	A	0.449595	34.08568053
Bread intake || id:ukb-b-11348	rs79436018	452236	-0.01761	0.003116	1.60E-08	C	T	0.116276	31.94700686
Bread intake || id:ukb-b-11348	rs7802468	452236	-0.02335	0.002056	6.90E-30	T	C	0.371501	128.9749813
Bread intake || id:ukb-b-11348	rs10761661	452236	-0.01149	0.002005	1.00E-08	T	C	0.45322	32.81776663
Bread intake || id:ukb-b-11348	rs55745436	452236	0.013425	0.002343	1.00E-08	T	C	0.237279	32.83891785
Bread intake || id:ukb-b-11348	rs1940033	452236	-0.01108	0.002028	4.70E-08	T	C	0.592727	29.831911
Bread intake || id:ukb-b-11348	rs11183201	452236	-0.01673	0.001996	5.30E-17	C	T	0.507856	70.23026857
Bread intake || id:ukb-b-11348	rs6580721	452236	0.017205	0.002544	1.30E-11	G	A	0.188665	45.7539713
Bread intake || id:ukb-b-11348	rs11060853	452236	-0.01279	0.002021	2.50E-10	G	A	0.411804	40.04943754
Bread intake || id:ukb-b-11348	rs9564268	452236	-0.01216	0.002049	3.00E-09	C	T	0.615619	35.21198027
Bread intake || id:ukb-b-11348	rs9529024	452236	-0.01292	0.002068	4.20E-10	T	A	0.370396	39.01657363
Bread intake || id:ukb-b-11348	rs11628639	452236	-0.0135	0.002322	6.20E-09	C	T	0.243333	33.76945832
Bread intake || id:ukb-b-11348	rs9323989	452236	-0.01161	0.002055	1.60E-08	C	T	0.379169	31.92741773
Bread intake || id:ukb-b-11348	rs28406095	452236	-0.01095	0.002	4.40E-08	A	G	0.461729	29.96655416
Bread intake || id:ukb-b-11348	rs4984685	452236	0.013585	0.002481	4.40E-08	A	G	0.201006	29.98602382
Bread intake || id:ukb-b-11348	rs62091167	452236	-0.01384	0.002425	1.20E-08	C	A	0.215931	32.56520365
Bread intake || id:ukb-b-11348	rs656817	452236	-0.01269	0.002109	1.80E-09	G	A	0.334442	36.18547597
Bread intake || id:ukb-b-11348	rs7276867	452236	0.011348	0.002003	1.50E-08	C	G	0.541677	32.09791005
Cereal intake || id:ukb-b-15926	rs10857964	441640	0.014087	0.002205	1.70E-10	C	T	0.205117	40.80158275
Cereal intake || id:ukb-b-15926	rs12354267	441640	0.011647	0.001933	1.70E-09	C	T	0.309145	36.31459321
Cereal intake || id:ukb-b-15926	rs112780312	441640	-0.01215	0.002019	1.80E-09	A	G	0.274969	36.20773852
Cereal intake || id:ukb-b-15926	rs184643	441640	-0.01217	0.001805	1.60E-11	A	G	0.56672	45.43770193
Cereal intake || id:ukb-b-15926	rs6545770	441640	-0.01373	0.00206	2.70E-11	T	A	0.748101	44.39225873
Cereal intake || id:ukb-b-15926	rs4988235	441640	0.0114	0.002012	1.50E-08	A	G	0.736893	32.09052108
Cereal intake || id:ukb-b-15926	rs67723420	441640	0.010525	0.001847	1.20E-08	A	T	0.376335	32.48564794
Cereal intake || id:ukb-b-15926	rs7619139	441640	-0.01695	0.001815	9.70E-21	A	T	0.588533	87.22524031
Cereal intake || id:ukb-b-15926	rs9846396	441640	0.011967	0.0018	3.00E-11	T	C	0.441556	44.18806933
Cereal intake || id:ukb-b-15926	rs11097340	441640	-0.01153	0.001815	2.10E-10	T	C	0.399592	40.37910276
Cereal intake || id:ukb-b-15926	rs3115230	441640	-0.01148	0.002071	3.00E-08	A	C	0.752001	30.70680772
Cereal intake || id:ukb-b-15926	rs11940694	441640	-0.01266	0.001834	5.00E-12	G	A	0.604068	47.67251045
Cereal intake || id:ukb-b-15926	rs10057775	441640	0.020029	0.002894	4.50E-12	C	T	0.893563	47.90717053
Cereal intake || id:ukb-b-15926	rs79642906	441640	-0.01816	0.003228	1.90E-08	A	G	0.083314	31.64036363
Cereal intake || id:ukb-b-15926	rs1853931	441640	-0.01134	0.00181	3.80E-10	A	G	0.531294	39.22312969
Cereal intake || id:ukb-b-15926	rs6918737	441640	0.013733	0.00211	7.60E-11	A	T	0.234488	42.36752626
Cereal intake || id:ukb-b-15926	rs2817377	441640	0.009901	0.001789	3.10E-08	A	G	0.537948	30.61703973
Cereal intake || id:ukb-b-15926	rs2504706	441640	0.018179	0.002102	5.30E-18	C	T	0.234682	74.77077467
Cereal intake || id:ukb-b-15926	rs9374896	441640	0.017527	0.001792	1.30E-22	T	C	0.466274	95.69385416
Cereal intake || id:ukb-b-15926	rs4410790	441640	-0.01091	0.001847	3.40E-09	C	T	0.630668	34.91982764
Cereal intake || id:ukb-b-15926	rs62442924	441640	0.012731	0.002257	1.70E-08	T	C	0.194276	31.81415996
Cereal intake || id:ukb-b-15926	rs13234131	441640	0.017012	0.00266	1.60E-10	G	A	0.128369	40.8946836
Cereal intake || id:ukb-b-15926	rs9987289	441640	0.017868	0.003095	7.80E-09	G	A	0.908767	33.33572591
Cereal intake || id:ukb-b-15926	rs4739095	441640	-0.01287	0.002105	9.90E-10	A	G	0.765732	37.34745298
Cereal intake || id:ukb-b-15926	rs2927238	441640	0.010247	0.001829	2.10E-08	G	T	0.613181	31.39197665
Cereal intake || id:ukb-b-15926	rs2799849	441640	-0.01233	0.001906	9.80E-11	T	C	0.678123	41.85293497
Cereal intake || id:ukb-b-15926	rs7040561	441640	-0.01627	0.00252	1.10E-10	A	T	0.850635	41.67609777
Cereal intake || id:ukb-b-15926	rs491711	441640	0.011689	0.001934	1.50E-09	C	A	0.311975	36.53905705
Cereal intake || id:ukb-b-15926	rs2450126	441640	-0.0149	0.002457	1.30E-09	G	A	0.156746	36.77447185
Cereal intake || id:ukb-b-15926	rs10837531	441640	0.010784	0.001797	2.00E-09	G	C	0.455002	36.01589606
Cereal intake || id:ukb-b-15926	rs11038810	441640	0.011134	0.001863	2.30E-09	G	A	0.644091	35.70948423

Cereal intake || id:ukb-b-15926	rs627185	441640	-0.01083	0.001791	1.50E-09	G	C	0.544427	36.55387994
Cereal intake || id:ukb-b-15926	rs2472297	441640	-0.01585	0.002022	4.50E-15	T	C	0.261458	61.46701454
Cereal intake || id:ukb-b-15926	rs1104608	441640	0.010863	0.001818	2.30E-09	C	G	0.426239	35.68604246
Cereal intake || id:ukb-b-15926	rs68136852	441640	-0.01412	0.002478	1.20E-08	A	C	0.152389	32.46638078
Cereal intake || id:ukb-b-15926	rs3859193	441640	-0.01033	0.001799	9.50E-09	A	T	0.470074	32.93551018
Cereal intake || id:ukb-b-15926	rs8097544	441640	-0.02464	0.002541	3.20E-22	G	A	0.145206	94.00088012
Cereal intake || id:ukb-b-15926	rs4797242	441640	0.011431	0.001949	4.50E-09	A	C	0.297207	34.4040042
Cereal intake || id:ukb-b-15926	rs11670024	441640	0.016011	0.002799	1.10E-08	G	A	0.115508	32.71199107
Cereal intake || id:ukb-b-15926	rs6510177	441640	-0.01304	0.002287	1.20E-08	C	T	0.805589	32.47501065
Cereal intake || id:ukb-b-15926	rs78854891	441640	0.022141	0.003633	1.10E-09	C	T	0.065728	37.13974364
Cereal intake || id:ukb-b-15926	rs56131196	441640	0.018002	0.002277	2.70E-15	A	G	0.188724	62.51062288
Cereal intake || id:ukb-b-15926	rs838133	441640	-0.02065	0.001843	3.90E-29	G	A	0.549201	125.5181256
Cheese intake || id:ukb-b-1489	rs78876700	451486	0.018097	0.003279	3.40E-08	A	G	0.137431	30.46726708
Cheese intake || id:ukb-b-1489	rs531358	451486	0.013167	0.002337	1.80E-08	T	C	0.649768	31.73325704
Cheese intake || id:ukb-b-1489	rs2802530	451486	0.018627	0.003397	4.20E-08	A	G	0.876502	30.06532171
Cheese intake || id:ukb-b-1489	rs6685323	451486	-0.01319	0.002416	4.80E-08	T	C	0.309293	29.80110002
Cheese intake || id:ukb-b-1489	rs2339928	451486	0.014858	0.002445	1.20E-09	A	G	0.704069	36.92943
Cheese intake || id:ukb-b-1489	rs12475594	451486	0.016007	0.002925	4.40E-08	G	A	0.178459	29.94545924
Cheese intake || id:ukb-b-1489	rs504675	451486	0.027442	0.002342	1.00E-31	T	C	0.352634	137.3150591
Cheese intake || id:ukb-b-1489	rs72970243	451486	0.022201	0.003402	6.70E-11	A	G	0.12044	42.59583927
Cheese intake || id:ukb-b-1489	rs1514755	451486	0.016375	0.002617	3.90E-10	G	A	0.239596	39.16629721
Cheese intake || id:ukb-b-1489	rs79184944	451486	0.019602	0.003285	2.40E-09	A	T	0.134346	35.61494018
Cheese intake || id:ukb-b-1489	rs4296548	451486	0.013025	0.002288	1.20E-08	G	T	0.609594	32.4102374
Cheese intake || id:ukb-b-1489	rs62245792	451486	-0.01793	0.003163	1.40E-08	A	T	0.150049	32.14505017
Cheese intake || id:ukb-b-1489	rs77742462	451486	-0.04747	0.008278	9.80E-09	G	A	0.020525	32.8794745
Cheese intake || id:ukb-b-1489	rs2352974	451486	-0.0145	0.002243	1.00E-10	T	C	0.48979	41.78180193
Cheese intake || id:ukb-b-1489	rs6774906	451486	0.031629	0.005673	2.50E-08	C	A	0.040653	31.08955626
Cheese intake || id:ukb-b-1489	rs4681981	451486	-0.01244	0.002241	2.90E-08	A	C	0.469086	30.78954762
Cheese intake || id:ukb-b-1489	rs4860341	451486	0.024365	0.004352	2.20E-08	C	T	0.928742	31.35147611
Cheese intake || id:ukb-b-1489	rs73096946	451486	-0.02059	0.003067	1.90E-11	C	T	0.157396	45.08646211
Cheese intake || id:ukb-b-1489	rs13107325	451486	-0.02916	0.004253	7.00E-12	T	C	0.074693	47.02524538
Cheese intake || id:ukb-b-1489	rs10938397	451486	-0.01271	0.002258	1.80E-08	G	A	0.434462	31.68352763
Cheese intake || id:ukb-b-1489	rs4692708	451486	0.01473	0.002589	1.30E-08	C	A	0.252659	32.36802342
Cheese intake || id:ukb-b-1489	rs26579	451486	-0.0128	0.002294	2.40E-08	C	G	0.586217	31.12994736
Cheese intake || id:ukb-b-1489	rs6873324	451486	-0.01248	0.002271	3.90E-08	C	A	0.425802	30.18988844
Cheese intake || id:ukb-b-1489	rs9504123	451486	0.014171	0.002504	1.50E-08	C	A	0.274743	32.02970398
Cheese intake || id:ukb-b-1489	rs975303	451486	0.021276	0.002907	2.50E-13	G	A	0.181313	53.56443861
Cheese intake || id:ukb-b-1489	rs1931805	451486	0.012636	0.002236	1.60E-08	C	T	0.500062	31.93972844
Cheese intake || id:ukb-b-1489	rs113367286	451486	0.01518	0.0025	1.30E-09	T	C	0.278467	36.87215126
Cheese intake || id:ukb-b-1489	rs34198643	451486	-0.0167	0.002679	4.50E-10	T	C	0.224165	38.87448361
Cheese intake || id:ukb-b-1489	rs12672200	451486	-0.01376	0.002395	9.00E-09	A	G	0.325794	33.03695536
Cheese intake || id:ukb-b-1489	rs9649582	451486	-0.01462	0.002412	1.40E-09	T	A	0.317256	36.73731282
Cheese intake || id:ukb-b-1489	rs7012814	451486	-0.01852	0.002254	2.10E-16	A	G	0.473966	67.48969346
Cheese intake || id:ukb-b-1489	rs7386207	451486	-0.0125	0.002269	3.60E-08	T	C	0.563517	30.33015659
Cheese intake || id:ukb-b-1489	rs13257887	451486	0.016181	0.002562	2.70E-10	C	T	0.358942	39.89231082
Cheese intake || id:ukb-b-1489	rs3911016	451486	0.021357	0.00344	5.30E-10	G	T	0.120938	38.54792995
Cheese intake || id:ukb-b-1489	rs4503172	451486	0.01296	0.002293	1.60E-08	T	C	0.608301	31.94295465
Cheese intake || id:ukb-b-1489	rs1806771	451486	-0.02215	0.004035	4.10E-08	G	T	0.087876	30.12527134
Cheese intake || id:ukb-b-1489	rs73335955	451486	0.027773	0.004978	2.40E-08	C	T	0.05334	31.12299753
Cheese intake || id:ukb-b-1489	rs10896050	451486	-0.01847	0.002834	7.20E-11	T	G	0.193171	42.46138221
Cheese intake || id:ukb-b-1489	rs67238148	451486	0.016543	0.002714	1.10E-09	T	G	0.217471	37.14641847
Cheese intake || id:ukb-b-1489	rs7936836	451486	0.015903	0.002273	2.60E-12	A	C	0.417538	48.96218478
Cheese intake || id:ukb-b-1489	rs73024305	451486	0.032537	0.004927	4.00E-11	C	G	0.054789	43.60799977
Cheese intake || id:ukb-b-1489	rs12786959	451486	-0.01607	0.00282	1.20E-08	T	A	0.196267	32.48144616
Cheese intake || id:ukb-b-1489	rs524468	451486	-0.01424	0.002552	2.40E-08	G	A	0.260633	31.15440566
Cheese intake || id:ukb-b-1489	rs1024853	451486	-0.01286	0.002262	1.30E-08	G	C	0.437902	32.32697644
Cheese intake || id:ukb-b-1489	rs7298331	451486	-0.01318	0.002306	1.10E-08	C	A	0.604531	32.64583963
Cheese intake || id:ukb-b-1489	rs12296440	451486	0.018794	0.00298	2.80E-10	A	G	0.169681	39.77945989
Cheese intake || id:ukb-b-1489	rs61953351	451486	0.014593	0.00258	1.50E-08	T	G	0.250366	31.99448498
Cheese intake || id:ukb-b-1489	rs1073242	451486	0.015728	0.002292	6.70E-12	A	G	0.553825	47.10690108
Cheese intake || id:ukb-b-1489	rs11620149	451486	-0.01767	0.003209	3.60E-08	C	T	0.143308	30.34521272
Cheese intake || id:ukb-b-1489	rs17115145	451486	-0.01288	0.00229	1.80E-08	T	C	0.401282	31.64959297
Cheese intake || id:ukb-b-1489	rs35270670	451486	0.016379	0.00271	1.50E-09	G	A	0.217962	36.53766027
Cheese intake || id:ukb-b-1489	rs4776970	451486	0.015409	0.002327	3.50E-11	T	A	0.357982	43.85391064
Cheese intake || id:ukb-b-1489	rs12447542	451486	0.019748	0.003407	6.80E-09	A	G	0.12558	33.59614718
Cheese intake || id:ukb-b-1489	rs61734410	451486	0.016654	0.002623	2.20E-10	T	C	0.255213	40.31903066
Cheese intake || id:ukb-b-1489	rs62034322	451486	-0.01394	0.0023	1.40E-09	A	G	0.379848	36.71489677
Cheese intake || id:ukb-b-1489	rs71386942	451486	0.014455	0.002522	9.90E-09	A	C	0.268947	32.85132137
Cheese intake || id:ukb-b-1489	rs11649653	451486	0.013849	0.002292	1.50E-09	G	C	0.38208	36.51688563
Cheese intake || id:ukb-b-1489	rs919109	451486	0.019938	0.003243	7.90E-10	C	G	0.138752	37.79203953
Cheese intake || id:ukb-b-1489	rs2854175	451486	0.016994	0.002569	3.70E-11	A	C	0.257474	43.77166659
Cheese intake || id:ukb-b-1489	rs12951057	451486	-0.02116	0.003044	3.60E-12	G	C	0.165627	48.30966088
Cheese intake || id:ukb-b-1489	rs2960578	451486	0.017028	0.002236	2.60E-14	G	T	0.496281	57.99855775
Cheese intake || id:ukb-b-1489	rs1434511	451486	0.012965	0.002259	9.50E-09	T	C	0.455286	32.94646557
Cheese intake || id:ukb-b-1489	rs1291145	451486	-0.02025	0.00241	4.40E-17	C	T	0.685848	70.58311508
Cheese intake || id:ukb-b-1489	rs6126641	451486	0.013226	0.002395	3.30E-08	A	G	0.336196	30.49825667
Cheese intake || id:ukb-b-1489	rs62236533	451486	0.024762	0.003647	1.10E-11	A	G	0.108791	46.105349
Coffee intake || id:ukb-b-5237	rs516636	428860	0.011677	0.001984	4.00E-09	A	C	0.208913	34.6316937
Coffee intake || id:ukb-b-5237	rs4615895	428860	0.012203	0.00185	4.20E-11	A	G	0.740926	43.51967545
Coffee intake || id:ukb-b-5237	rs13387939	428860	0.016556	0.002139	9.80E-15	A	C	0.828363	59.92957774
Coffee intake || id:ukb-b-5237	rs780093	428860	0.013294	0.001657	1.00E-15	C	T	0.615839	64.36649682
Coffee intake || id:ukb-b-5237	rs12989746	428860	0.01035	0.001864	2.80E-08	T	G	0.249928	30.8214992
Coffee intake || id:ukb-b-5237	rs1527961	428860	-0.01334	0.002366	1.70E-08	C	T	0.1349	31.80819827
Coffee intake || id:ukb-b-5237	rs2597805	428860	0.009855	0.001756	2.00E-08	T	C	0.682463	31.48851914
Coffee intake || id:ukb-b-5237	rs2189234	428860	0.009987	0.001661	1.80E-09	G	T	0.617795	36.17198243
Coffee intake || id:ukb-b-5237	rs13163336	428860	0.014947	0.00221	1.30E-11	A	C	0.15761	45.74203614
Coffee intake || id:ukb-b-5237	rs12514566	428860	-0.0114	0.001706	2.40E-11	A	G	0.337107	44.65106059

Coffee intake || id:ukb-b-5237	rs2465037	428860	-0.01063	0.001707	4.80E-10	A	C	0.343017	38.77212562
Coffee intake || id:ukb-b-5237	rs1338549	428860	-0.00945	0.001622	5.60E-09	G	T	0.533932	33.96098155
Coffee intake || id:ukb-b-5237	rs9398171	428860	0.010858	0.00178	1.10E-09	T	C	0.71064	37.21003427
Coffee intake || id:ukb-b-5237	rs73075167	428860	-0.01606	0.002444	5.00E-11	T	A	0.12918	43.19132446
Coffee intake || id:ukb-b-5237	rs7811609	428860	0.009139	0.001665	4.00E-08	T	C	0.374746	30.13711083
Coffee intake || id:ukb-b-5237	rs1057868	428860	0.019951	0.001785	5.40E-29	T	C	0.284986	124.9009737
Coffee intake || id:ukb-b-5237	rs4410790	428860	0.039072	0.001673	1.20E-120	C	T	0.632141	545.5087317
Coffee intake || id:ukb-b-5237	rs34060476	428860	0.018429	0.00237	7.50E-15	G	A	0.133855	60.44986565
Coffee intake || id:ukb-b-5237	rs6469262	428860	-0.00915	0.001629	1.90E-08	C	T	0.564966	31.57592028
Coffee intake || id:ukb-b-5237	rs78267637	428860	-0.02543	0.004317	3.90E-09	G	C	0.038115	34.69533367
Coffee intake || id:ukb-b-5237	rs442355	428860	-0.01114	0.001854	1.90E-09	C	G	0.254435	36.09561078
Coffee intake || id:ukb-b-5237	rs10119174	428860	-0.0094	0.001642	1.00E-08	C	G	0.571035	32.77393185
Coffee intake || id:ukb-b-5237	rs117810762	428860	0.035909	0.006179	6.20E-09	A	G	0.017881	33.77546389
Coffee intake || id:ukb-b-5237	rs61928609	428860	-0.01473	0.002175	1.30E-11	C	A	0.835328	45.85352204
Coffee intake || id:ukb-b-5237	rs2472297	428860	0.046471	0.001827	1.10E-142	T	C	0.262883	646.7349474
Coffee intake || id:ukb-b-5237	rs117968677	428860	-0.03103	0.005516	1.90E-08	A	G	0.024207	31.6454053
Coffee intake || id:ukb-b-5237	rs8056750	428860	0.010533	0.001737	1.30E-09	T	C	0.359129	36.77640515
Coffee intake || id:ukb-b-5237	rs1421085	428860	0.018543	0.001644	1.70E-29	C	T	0.40357	127.159102
Coffee intake || id:ukb-b-5237	rs62064918	428860	-0.01031	0.001879	4.10E-08	T	C	0.244545	30.10111407
Coffee intake || id:ukb-b-5237	rs57918684	428860	0.012886	0.002238	8.60E-09	A	G	0.154747	33.14121451
Coffee intake || id:ukb-b-5237	rs7224815	428860	-0.01086	0.001642	3.70E-11	T	A	0.407832	43.76534713
Coffee intake || id:ukb-b-5237	rs630194	428860	-0.01135	0.001699	2.30E-11	C	T	0.343374	44.67841767
Coffee intake || id:ukb-b-5237	rs1942965	428860	-0.0089	0.001619	3.80E-08	C	T	0.504585	30.23610867
Coffee intake || id:ukb-b-5237	rs476828	428860	0.017346	0.001895	5.60E-20	C	T	0.237409	83.75065094
Coffee intake || id:ukb-b-5237	rs56113850	428860	0.012667	0.001634	8.90E-15	C	T	0.578109	60.12891602
Coffee intake || id:ukb-b-5237	rs75347775	428860	0.01045	0.001879	2.70E-08	A	G	0.244531	30.93296233
Coffee intake || id:ukb-b-5237	rs6063085	428860	0.010411	0.001669	4.50E-10	C	A	0.373473	38.8991375
Coffee intake || id:ukb-b-5237	rs6062682	428860	0.01037	0.001639	2.50E-10	T	C	0.464546	40.02021679
Coffee intake || id:ukb-b-5237	rs13054099	428860	-0.01078	0.001836	4.30E-09	C	T	0.261004	34.46045604
Coffee intake || id:ukb-b-5237	rs17842490	428860	-0.04517	0.006808	3.30E-11	G	A	0.014248	44.01160013
Cooked vegetable intake || id:ukb-b-8089	rs2252508	448651	0.009102	0.001562	5.70E-09	G	A	0.480256	33.94940589
Cooked vegetable intake || id:ukb-b-8089	rs2102738	448651	-0.01216	0.002083	5.30E-09	C	A	0.172403	34.06615436
Cooked vegetable intake || id:ukb-b-8089	rs4851029	448651	0.010174	0.001564	7.80E-11	G	T	0.526969	42.30740555
Cooked vegetable intake || id:ukb-b-8089	rs12629972	448651	0.0118	0.001591	1.20E-13	C	T	0.588311	55.01758267
Cooked vegetable intake || id:ukb-b-8089	rs28450747	448651	-0.01016	0.001855	4.30E-08	A	G	0.23257	30.01057162
Cooked vegetable intake || id:ukb-b-8089	rs1816263	448651	0.009581	0.001741	3.70E-08	C	T	0.280188	30.29902797
Cooked vegetable intake || id:ukb-b-8089	rs2844672	448651	-0.00964	0.001609	2.10E-09	A	G	0.624125	35.90141171
Cooked vegetable intake || id:ukb-b-8089	rs12550717	448651	0.009187	0.001619	1.40E-08	A	G	0.372413	32.20362875
Cooked vegetable intake || id:ukb-b-8089	rs10156602	448651	0.011006	0.001637	1.80E-11	G	A	0.361369	45.21206197
Cooked vegetable intake || id:ukb-b-8089	rs11138705	448651	0.010372	0.00183	1.40E-08	C	G	0.757204	32.11899728
Cooked vegetable intake || id:ukb-b-8089	rs2052063	448651	-0.00946	0.001568	1.60E-09	T	C	0.515902	36.38484857
Cooked vegetable intake || id:ukb-b-8089	rs349062	448651	-0.0089	0.001598	2.50E-08	C	G	0.39275	31.06003887
Cooked vegetable intake || id:ukb-b-8089	rs28711392	448651	-0.01075	0.001632	4.60E-11	C	T	0.367132	43.34500363
Cooked vegetable intake || id:ukb-b-8089	rs10161952	448651	-0.00958	0.001686	1.30E-08	C	A	0.31274	32.28898664
Cooked vegetable intake || id:ukb-b-8089	rs1421085	448651	0.010332	0.001591	8.30E-11	C	T	0.40346	42.1935412
Cooked vegetable intake || id:ukb-b-8089	rs838133	448651	0.011688	0.001615	4.50E-13	G	A	0.549575	52.39638412
Cooked vegetable intake || id:ukb-b-8089	rs34155012	448651	0.010558	0.001922	3.90E-08	T	C	0.227386	30.17541854
Dried fruit intake || id:ukb-b-16576	rs261809	421764	-0.00963	0.001679	9.80E-09	G	A	0.540636	32.88612836
Dried fruit intake || id:ukb-b-16576	rs11586016	421764	0.009878	0.00173	1.10E-08	C	G	0.371004	32.59049647
Dried fruit intake || id:ukb-b-16576	rs12137234	421764	0.010205	0.001837	2.80E-08	T	C	0.303772	30.84666355
Dried fruit intake || id:ukb-b-16576	rs72720396	421764	0.011427	0.001985	8.70E-09	G	A	0.229157	33.1227234
Dried fruit intake || id:ukb-b-16576	rs11811826	421764	0.013218	0.002006	4.40E-11	A	T	0.224231	43.42532711
Dried fruit intake || id:ukb-b-16576	rs3101339	421764	0.01426	0.001705	6.20E-17	C	A	0.603285	69.90778136
Dried fruit intake || id:ukb-b-16576	rs75641275	421764	-0.01416	0.002385	2.90E-09	C	A	0.143372	35.25039016
Dried fruit intake || id:ukb-b-16576	rs7582086	421764	-0.00963	0.001674	8.80E-09	T	G	0.468273	33.08863044
Dried fruit intake || id:ukb-b-16576	rs7599488	421764	-0.01042	0.001687	6.70E-10	T	C	0.426408	38.10416437
Dried fruit intake || id:ukb-b-16576	rs4149513	421764	0.01173	0.001671	2.20E-12	A	G	0.493537	49.25387744
Dried fruit intake || id:ukb-b-16576	rs17184707	421764	-0.01144	0.00204	2.10E-08	T	C	0.212811	31.4340695
Dried fruit intake || id:ukb-b-16576	rs4269101	421764	-0.01381	0.001859	1.10E-13	G	T	0.718948	55.17269195
Dried fruit intake || id:ukb-b-16576	rs11720884	421764	0.01118	0.001936	7.60E-09	G	A	0.250137	33.3623011
Dried fruit intake || id:ukb-b-16576	rs57499472	421764	0.009912	0.001719	8.10E-09	C	T	0.404131	33.26248192
Dried fruit intake || id:ukb-b-16576	rs10026792	421764	0.010847	0.001842	3.90E-09	A	G	0.290404	34.6623926
Dried fruit intake || id:ukb-b-16576	rs1648404	421764	0.009416	0.001674	1.80E-08	T	C	0.476112	31.65486917
Dried fruit intake || id:ukb-b-16576	rs746868	421764	-0.01291	0.001715	5.20E-14	G	C	0.614662	56.65843301
Dried fruit intake || id:ukb-b-16576	rs9385269	421764	0.012067	0.001682	7.20E-13	T	C	0.524565	51.48207338
Dried fruit intake || id:ukb-b-16576	rs2328887	421764	0.018948	0.002776	8.80E-12	C	T	0.899467	46.58811024
Dried fruit intake || id:ukb-b-16576	rs2533273	421764	-0.00988	0.001677	3.90E-09	A	C	0.48453	34.6797068
Dried fruit intake || id:ukb-b-16576	rs7808471	421764	-0.01154	0.001786	1.10E-10	C	T	0.322136	41.71989351
Dried fruit intake || id:ukb-b-16576	rs11772627	421764	0.018334	0.002171	3.00E-17	C	G	0.18202	71.31638696
Dried fruit intake || id:ukb-b-16576	rs7829800	421764	-0.01045	0.001787	5.10E-09	G	A	0.671041	34.16898975
Dried fruit intake || id:ukb-b-16576	rs10740991	421764	0.016739	0.001857	2.00E-19	C	G	0.717606	81.23296683
Dried fruit intake || id:ukb-b-16576	rs7916868	421764	0.009607	0.001672	9.10E-09	T	A	0.503499	33.03022654
Dried fruit intake || id:ukb-b-16576	rs893856	421764	-0.01336	0.002349	1.30E-08	A	G	0.148988	32.34646694
Dried fruit intake || id:ukb-b-16576	rs10896126	421764	-0.01501	0.001819	1.60E-16	G	A	0.303582	68.0686904
Dried fruit intake || id:ukb-b-16576	rs11037497	421764	0.01044	0.001684	5.70E-10	C	G	0.446175	38.41200163
Dried fruit intake || id:ukb-b-16576	rs1622515	421764	0.009917	0.001671	2.90E-09	G	A	0.484704	35.23253812
Dried fruit intake || id:ukb-b-16576	rs3764002	421764	0.013122	0.001901	5.10E-12	T	C	0.261416	47.6529239
Dried fruit intake || id:ukb-b-16576	rs4140799	421764	0.009457	0.001678	1.80E-08	A	G	0.531856	31.7430057
Dried fruit intake || id:ukb-b-16576	rs34162196	421764	-0.02236	0.002772	7.10E-16	T	C	0.101001	65.09778077
Dried fruit intake || id:ukb-b-16576	rs10129747	421764	0.009359	0.001681	2.60E-08	G	A	0.530254	30.98781751
Dried fruit intake || id:ukb-b-16576	rs1797235	421764	-0.01002	0.001742	8.90E-09	C	G	0.374623	33.07627952
Dried fruit intake || id:ukb-b-16576	rs11632215	421764	-0.01414	0.002584	4.40E-08	C	A	0.120179	29.96285118
Dried fruit intake || id:ukb-b-16576	rs862227	421764	-0.00916	0.001672	4.30E-08	G	A	0.458327	30.03012213
Dried fruit intake || id:ukb-b-16576	rs1582322	421764	0.009943	0.001716	6.80E-09	G	A	0.604805	33.58824381
Dried fruit intake || id:ukb-b-16576	rs62084586	421764	0.013395	0.002262	3.20E-09	C	T	0.165729	35.07309892
Dried fruit intake || id:ukb-b-16576	rs8081370	421764	-0.01667	0.002938	1.40E-08	T	C	0.910232	32.18142153
Dried fruit intake || id:ukb-b-16576	rs4800488	421764	0.011984	0.001672	7.70E-13	A	C	0.489858	51.36788358

Dried fruit intake || id:ukb-b-16576	rs17175518	421764	0.011496	0.001975	5.90E-09	A	C	0.232775	33.87761691
Dried fruit intake || id:ukb-b-16576	rs11152349	421764	0.009912	0.001818	4.90E-08	A	G	0.302856	29.73987255
Dried fruit intake || id:ukb-b-16576	rs429358	421764	0.019945	0.002314	6.70E-18	C	T	0.154208	74.31256412
Fresh fruit intake || id:ukb-b-3881	rs2790688	446462	0.011447	0.001696	1.50E-11	T	C	0.154068	45.54437451
Fresh fruit intake || id:ukb-b-3881	rs559734	446462	0.007768	0.001361	1.10E-08	C	G	0.711817	32.58736969
Fresh fruit intake || id:ukb-b-3881	rs12044599	446462	0.009421	0.001503	3.70E-10	G	A	0.21008	39.27152797
Fresh fruit intake || id:ukb-b-3881	rs7554485	446462	-0.00801	0.001254	1.70E-10	C	T	0.611539	40.79323752
Fresh fruit intake || id:ukb-b-3881	rs1620977	446462	-0.01317	0.001378	1.10E-21	G	A	0.730517	91.45257352
Fresh fruit intake || id:ukb-b-3881	rs2867113	446462	-0.01386	0.00196	1.50E-12	A	G	0.130942	49.99667651
Fresh fruit intake || id:ukb-b-3881	rs17049185	446462	0.00804	0.00139	7.30E-09	T	G	0.267872	33.44435024
Fresh fruit intake || id:ukb-b-3881	rs11896330	446462	-0.00845	0.001274	3.40E-11	A	G	0.632839	43.91942955
Fresh fruit intake || id:ukb-b-3881	rs72974263	446462	0.007387	0.001312	1.80E-08	T	C	0.318282	31.67901755
Fresh fruit intake || id:ukb-b-3881	rs817223	446462	-0.00727	0.001223	2.80E-09	C	T	0.481314	35.30441703
Fresh fruit intake || id:ukb-b-3881	rs4953150	446462	-0.00844	0.001293	6.60E-11	T	C	0.344088	42.62079328
Fresh fruit intake || id:ukb-b-3881	rs10192394	446462	-0.00766	0.001229	4.50E-10	T	C	0.528785	38.87538231
Fresh fruit intake || id:ukb-b-3881	rs1375566	446462	-0.00783	0.001266	6.10E-10	A	G	0.627412	38.2895062
Fresh fruit intake || id:ukb-b-3881	rs13072255	446462	0.00898	0.001224	2.10E-13	C	A	0.493966	53.86675793
Fresh fruit intake || id:ukb-b-3881	rs1356292	446462	0.009175	0.001553	3.50E-09	T	C	0.807546	34.89052938
Fresh fruit intake || id:ukb-b-3881	rs12641371	446462	0.007913	0.001234	1.40E-10	T	C	0.433148	41.14755406
Fresh fruit intake || id:ukb-b-3881	rs10064431	446462	-0.00757	0.001224	6.00E-10	C	T	0.522495	38.31130962
Fresh fruit intake || id:ukb-b-3881	rs149449	446462	0.007289	0.001221	2.40E-09	A	G	0.489167	35.65565571
Fresh fruit intake || id:ukb-b-3881	rs2143081	446462	0.008321	0.001229	1.30E-11	A	G	0.539822	45.84402103
Fresh fruit intake || id:ukb-b-3881	rs586346	446462	-0.00692	0.001266	4.50E-08	C	T	0.635353	29.900247
Fresh fruit intake || id:ukb-b-3881	rs994270	446462	0.013259	0.001444	4.20E-20	G	C	0.235126	84.31805282
Fresh fruit intake || id:ukb-b-3881	rs329274	446462	0.006818	0.001228	2.80E-08	G	A	0.485636	30.82219272
Fresh fruit intake || id:ukb-b-3881	rs10271924	446462	-0.00705	0.001256	2.00E-08	T	C	0.492595	31.48147035
Fresh fruit intake || id:ukb-b-3881	rs10249294	446462	0.019564	0.001263	4.10E-54	A	G	0.372973	239.9397296
Fresh fruit intake || id:ukb-b-3881	rs12536253	446462	-0.00816	0.001416	8.30E-09	C	G	0.24901	33.20467771
Fresh fruit intake || id:ukb-b-3881	rs7818437	446462	-0.00805	0.001453	3.00E-08	C	T	0.235903	30.70109476
Fresh fruit intake || id:ukb-b-3881	rs1866823	446462	0.007431	0.001241	2.10E-09	A	G	0.544469	35.87619883
Fresh fruit intake || id:ukb-b-3881	rs6475724	446462	0.007722	0.001374	1.90E-08	T	C	0.727296	31.57959681
Fresh fruit intake || id:ukb-b-3881	rs7869969	446462	0.007567	0.001299	5.70E-09	G	A	0.330836	33.92702255
Fresh fruit intake || id:ukb-b-3881	rs4302893	446462	0.007389	0.0013	1.30E-08	A	G	0.334189	32.3227321
Fresh fruit intake || id:ukb-b-3881	rs2093654	446462	0.007129	0.001259	1.50E-08	G	A	0.388133	32.07771602
Fresh fruit intake || id:ukb-b-3881	rs11248509	446462	0.00733	0.001268	7.40E-09	T	A	0.371237	33.41416379
Fresh fruit intake || id:ukb-b-3881	rs9919429	446462	-0.00672	0.001223	3.80E-08	G	A	0.486008	30.22594293
Fresh fruit intake || id:ukb-b-3881	rs10828266	446462	0.012364	0.001357	8.10E-20	G	A	0.715634	83.02290993
Fresh fruit intake || id:ukb-b-3881	rs12780952	446462	0.007475	0.001354	3.40E-08	A	G	0.286382	30.4919584
Fresh fruit intake || id:ukb-b-3881	rs10840126	446462	-0.00772	0.001286	1.90E-09	G	A	0.376121	36.02781772
Fresh fruit intake || id:ukb-b-3881	rs10838724	446462	0.009011	0.001283	2.10E-12	T	G	0.367992	49.34428076
Fresh fruit intake || id:ukb-b-3881	rs11032362	446462	0.012393	0.002123	5.30E-09	A	G	0.090976	34.06327143
Fresh fruit intake || id:ukb-b-3881	rs60452247	446462	0.00797	0.001269	3.40E-10	A	G	0.363079	39.42217772
Fresh fruit intake || id:ukb-b-3881	rs7982441	446462	-0.00841	0.001376	9.80E-10	C	T	0.731883	37.37086732
Fresh fruit intake || id:ukb-b-3881	rs9517948	446462	0.006953	0.001233	1.70E-08	T	C	0.451352	31.80743346
Fresh fruit intake || id:ukb-b-3881	rs12885598	446462	0.007515	0.001247	1.70E-09	A	G	0.596671	36.30087134
Fresh fruit intake || id:ukb-b-3881	rs34162196	446462	-0.01814	0.00203	4.00E-19	T	C	0.1008	79.88997008
Fresh fruit intake || id:ukb-b-3881	rs28479795	446462	0.011232	0.001473	2.50E-14	T	C	0.221473	58.11313644
Fresh fruit intake || id:ukb-b-3881	rs62051554	446462	0.011612	0.001981	4.60E-09	A	G	0.108545	34.34326853
Fresh fruit intake || id:ukb-b-3881	rs862227	446462	-0.01015	0.001224	1.10E-16	G	A	0.457858	68.70250671
Fresh fruit intake || id:ukb-b-3881	rs1051547	446462	-0.00758	0.001242	1.10E-09	C	T	0.561537	37.22384605
Fresh fruit intake || id:ukb-b-3881	rs139042899	446462	0.035995	0.006081	3.20E-09	C	A	0.013469	35.03690849
Fresh fruit intake || id:ukb-b-3881	rs2048522	446462	0.009563	0.001248	1.80E-14	T	A	0.434968	58.73751713
Fresh fruit intake || id:ukb-b-3881	rs73455661	446462	0.010294	0.001362	4.10E-14	G	A	0.279338	57.10441675
Fresh fruit intake || id:ukb-b-3881	rs8095324	446462	-0.00695	0.001249	2.70E-08	G	A	0.404041	30.9271624
Fresh fruit intake || id:ukb-b-3881	rs11085749	446462	-0.00773	0.001255	7.10E-10	A	G	0.387076	37.98711232
Fresh fruit intake || id:ukb-b-3881	rs2302593	446462	0.008528	0.001223	3.10E-12	G	C	0.486625	48.62081118
Fresh fruit intake || id:ukb-b-3881	rs739320	446462	-0.009	0.001277	1.90E-12	C	T	0.60575	49.63512973
Fresh fruit intake || id:ukb-b-3881	rs78537042	446462	-0.01193	0.002184	4.80E-08	A	C	0.086801	29.80916441
Lamb/mutton intake || id:ukb-b-14179	rs7550173	460006	-0.00912	0.001502	1.30E-09	T	A	0.610281	36.88458002
Lamb/mutton intake || id:ukb-b-14179	rs660880	460006	-0.00903	0.001464	6.80E-10	A	G	0.51281	38.06426421
Lamb/mutton intake || id:ukb-b-14179	rs56394517	460006	-0.01377	0.002491	3.20E-08	G	A	0.09584	30.57354321
Lamb/mutton intake || id:ukb-b-14179	rs139237013	460006	0.018899	0.00314	1.80E-09	A	G	0.057668	36.22383231
Lamb/mutton intake || id:ukb-b-14179	rs2222760	460006	-0.00909	0.001637	2.80E-08	A	G	0.28089	30.84381164
Lamb/mutton intake || id:ukb-b-14179	rs62106258	460006	0.02163	0.003402	2.00E-10	C	T	0.048562	40.4369077
Lamb/mutton intake || id:ukb-b-14179	rs2678900	460006	0.010084	0.001481	9.90E-12	G	T	0.427924	46.35307872
Lamb/mutton intake || id:ukb-b-14179	rs2140714	460006	-0.00852	0.001476	7.70E-09	G	C	0.557738	33.34108908
Lamb/mutton intake || id:ukb-b-14179	rs12634740	460006	-0.01009	0.001697	2.80E-09	G	T	0.252078	35.3238662
Lamb/mutton intake || id:ukb-b-14179	rs6829572	460006	0.008401	0.001474	1.20E-08	A	G	0.456717	32.48116867
Lamb/mutton intake || id:ukb-b-14179	rs16891982	460006	-0.02427	0.004364	2.70E-08	G	C	0.972143	30.91921278
Lamb/mutton intake || id:ukb-b-14179	rs11743441	460006	-0.00884	0.001486	2.70E-09	T	G	0.574343	35.4081582
Lamb/mutton intake || id:ukb-b-14179	rs7447465	460006	0.009582	0.001506	2.00E-10	C	T	0.619449	40.47399311
Lamb/mutton intake || id:ukb-b-14179	rs62398404	460006	0.012915	0.002194	4.00E-09	T	C	0.127211	34.64173075
Lamb/mutton intake || id:ukb-b-14179	rs994270	460006	0.00982	0.001729	1.40E-08	G	C	0.234851	32.24173634
Lamb/mutton intake || id:ukb-b-14179	rs35797675	460006	-0.01085	0.001793	1.40E-09	G	T	0.215928	36.60067834
Lamb/mutton intake || id:ukb-b-14179	rs4272399	460006	-0.00924	0.001576	4.50E-09	A	C	0.321491	34.40571043
Lamb/mutton intake || id:ukb-b-14179	rs276453	460006	-0.01424	0.001467	2.90E-22	C	A	0.488356	94.15438988
Lamb/mutton intake || id:ukb-b-14179	rs1556147	460006	0.009101	0.001559	5.30E-09	T	A	0.671626	34.07775792
Lamb/mutton intake || id:ukb-b-14179	rs673696	460006	0.015821	0.002682	3.70E-09	T	C	0.080995	34.79576423
Lamb/mutton intake || id:ukb-b-14179	rs4489752	460006	0.013811	0.001976	2.80E-12	T	G	0.836007	48.85825051
Lamb/mutton intake || id:ukb-b-14179	rs6581296	460006	0.01002	0.001824	4.00E-08	G	C	0.794714	30.16615614
Lamb/mutton intake || id:ukb-b-14179	rs3105056	460006	-0.01162	0.001649	1.80E-12	C	T	0.732738	49.71869219
Lamb/mutton intake || id:ukb-b-14179	rs1958801	460006	-0.00894	0.001617	3.20E-08	G	A	0.287998	30.57536372
Lamb/mutton intake || id:ukb-b-14179	rs55813438	460006	-0.01142	0.001735	4.70E-11	A	G	0.763165	43.3000708
Lamb/mutton intake || id:ukb-b-14179	rs2726033	460006	-0.00949	0.00148	1.50E-10	G	A	0.422384	41.09066335
Lamb/mutton intake || id:ukb-b-14179	rs3964074	460006	-0.00814	0.001472	3.20E-08	C	T	0.547043	30.58063405
Lamb/mutton intake || id:ukb-b-14179	rs2926119	460006	0.008111	0.001482	4.40E-08	A	C	0.569417	29.96387466
Lamb/mutton intake || id:ukb-b-14179	rs429358	460006	-0.0182	0.002027	2.70E-19	C	T	0.154171	80.61155081

Lamb/mutton intake || id:ukb-b-14179	rs17270057	460006	0.012653	0.002309	4.30E-08	C	T	0.113347	30.03108543
Lamb/mutton intake || id:ukb-b-14179	rs136548	460006	0.009532	0.001512	2.90E-10	T	C	0.376668	39.73257881
Lamb/mutton intake || id:ukb-b-14179	rs11090045	460006	-0.01068	0.001607	3.00E-11	A	G	0.307145	44.15968432
Non-oily fish intake || id:ukb-b-17627	rs16822430	460880	0.011634	0.00192	1.40E-09	C	T	0.23321	36.72008016
Non-oily fish intake || id:ukb-b-17627	rs1260326	460880	-0.00955	0.001655	7.90E-09	C	T	0.604249	33.31004524
Non-oily fish intake || id:ukb-b-17627	rs11680516	460880	0.012284	0.002028	1.40E-09	C	T	0.202388	36.68036582
Non-oily fish intake || id:ukb-b-17627	rs3799077	460880	-0.01073	0.001758	1.00E-09	G	T	0.309993	37.30026154
Non-oily fish intake || id:ukb-b-17627	rs4318925	460880	-0.01504	0.002121	1.30E-12	T	C	0.177245	50.28105253
Non-oily fish intake || id:ukb-b-17627	rs6957745	460880	-0.01218	0.002023	1.80E-09	C	T	0.202978	36.23310369
Non-oily fish intake || id:ukb-b-17627	rs17317920	460880	0.00906	0.001631	2.80E-08	G	A	0.479238	30.85103737
Non-oily fish intake || id:ukb-b-17627	rs35287743	460880	-0.01774	0.002552	3.60E-12	T	G	0.11583	48.30599625
Non-oily fish intake || id:ukb-b-17627	rs7148387	460880	-0.00931	0.00165	1.70E-08	G	A	0.590715	31.80433145
Non-oily fish intake || id:ukb-b-17627	rs56094641	460880	0.012588	0.001651	2.50E-14	G	A	0.404619	58.12229223
Non-oily fish intake || id:ukb-b-17627	rs838133	460880	0.016184	0.001676	4.70E-22	G	A	0.549409	93.21679306
Oily fish intake || id:ukb-b-2209	rs973526	460443	-0.01151	0.00193	2.50E-09	T	C	0.513307	35.56154676
Oily fish intake || id:ukb-b-2209	rs45501495	460443	0.01568	0.002257	3.70E-12	T	C	0.236013	48.26091912
Oily fish intake || id:ukb-b-2209	rs55930451	460443	-0.01705	0.003076	2.90E-08	T	C	0.108001	30.74453656
Oily fish intake || id:ukb-b-2209	rs55985303	460443	0.012974	0.002236	6.60E-09	A	G	0.241079	33.65669436
Oily fish intake || id:ukb-b-2209	rs17050031	460443	-0.01205	0.001921	3.50E-10	T	C	0.4801	39.35476772
Oily fish intake || id:ukb-b-2209	rs275160	460443	0.012119	0.002101	8.00E-09	C	T	0.700597	33.27236476
Oily fish intake || id:ukb-b-2209	rs13070166	460443	0.014211	0.002278	4.40E-10	A	T	0.228586	38.91072627
Oily fish intake || id:ukb-b-2209	rs114497213	460443	0.027323	0.004236	1.10E-10	T	G	0.05481	41.60243148
Oily fish intake || id:ukb-b-2209	rs10513136	460443	-0.02332	0.003863	1.60E-09	A	G	0.065424	36.45114017
Oily fish intake || id:ukb-b-2209	rs1876245	460443	0.015116	0.001931	5.00E-15	C	T	0.431482	61.26631478
Oily fish intake || id:ukb-b-2209	rs10510554	460443	0.011045	0.001936	1.20E-08	C	T	0.569277	32.53327454
Oily fish intake || id:ukb-b-2209	rs905575	460443	0.013882	0.002519	3.60E-08	G	C	0.82397	30.36785772
Oily fish intake || id:ukb-b-2209	rs9841174	460443	0.014778	0.00198	8.50E-14	C	T	0.373913	55.69255701
Oily fish intake || id:ukb-b-2209	rs1201289	460443	-0.01073	0.00196	4.40E-08	G	T	0.394525	29.96577668
Oily fish intake || id:ukb-b-2209	rs7683782	460443	0.014478	0.002574	1.90E-08	G	C	0.833405	31.63540776
Oily fish intake || id:ukb-b-2209	rs10076975	460443	0.011239	0.001967	1.10E-08	C	T	0.381413	32.64293677
Oily fish intake || id:ukb-b-2209	rs10061973	460443	-0.01085	0.001916	1.50E-08	T	G	0.5139	32.08083262
Oily fish intake || id:ukb-b-2209	rs16891727	460443	-0.02372	0.00284	6.80E-17	A	C	0.129814	69.74043288
Oily fish intake || id:ukb-b-2209	rs34555420	460443	-0.02377	0.003219	1.50E-13	T	G	0.097821	54.51388329
Oily fish intake || id:ukb-b-2209	rs12663865	460443	0.012772	0.002232	1.10E-08	A	G	0.758185	32.73238742
Oily fish intake || id:ukb-b-2209	rs4869859	460443	0.01401	0.001922	3.10E-13	C	T	0.449939	53.12824506
Oily fish intake || id:ukb-b-2209	rs11767283	460443	0.017674	0.002319	2.50E-14	G	A	0.221709	58.06074287
Oily fish intake || id:ukb-b-2209	rs6465487	460443	-0.01235	0.001956	2.70E-10	G	A	0.399814	39.87658965
Oily fish intake || id:ukb-b-2209	rs11986122	460443	0.014817	0.001949	2.90E-14	G	C	0.422568	57.77687487
Oily fish intake || id:ukb-b-2209	rs790564	460443	0.014691	0.002148	7.90E-12	C	A	0.722952	46.78945758
Oily fish intake || id:ukb-b-2209	rs552234	460443	-0.01165	0.001913	1.10E-09	A	G	0.495449	37.08907567
Oily fish intake || id:ukb-b-2209	rs9886779	460443	-0.01072	0.001929	2.70E-08	A	T	0.439213	30.8942246
Oily fish intake || id:ukb-b-2209	rs10828250	460443	-0.02015	0.002073	2.60E-22	G	C	0.309332	94.40954499
Oily fish intake || id:ukb-b-2209	rs703987	460443	0.011122	0.001974	1.70E-08	C	G	0.615005	31.75580212
Oily fish intake || id:ukb-b-2209	rs61882686	460443	0.019756	0.003425	8.00E-09	A	C	0.085234	33.26373802
Oily fish intake || id:ukb-b-2209	rs4278546	460443	0.012556	0.001938	9.30E-11	G	A	0.441085	41.96948001
Oily fish intake || id:ukb-b-2209	rs2374424	460443	-0.01144	0.001956	4.90E-09	G	A	0.601525	34.22593037
Oily fish intake || id:ukb-b-2209	rs510161	460443	-0.0113	0.002066	4.50E-08	G	C	0.31037	29.91009679
Oily fish intake || id:ukb-b-2209	rs631490	460443	-0.01514	0.002103	6.00E-13	C	G	0.709106	51.85084921
Oily fish intake || id:ukb-b-2209	rs303817	460443	0.01358	0.00221	8.00E-10	G	A	0.751146	37.76992884
Oily fish intake || id:ukb-b-2209	rs35287743	460443	-0.02822	0.003011	7.00E-21	T	G	0.115869	87.85758202
Oily fish intake || id:ukb-b-2209	rs9597870	460443	-0.01274	0.00223	1.10E-08	G	T	0.245844	32.65006017
Oily fish intake || id:ukb-b-2209	rs3124402	460443	-0.022	0.002157	1.90E-24	G	A	0.733261	104.0872851
Oily fish intake || id:ukb-b-2209	rs12855717	460443	-0.01223	0.001923	2.00E-10	T	C	0.526774	40.45694357
Oily fish intake || id:ukb-b-2209	rs1361016	460443	0.014957	0.002653	1.70E-08	G	T	0.844853	31.7843574
Oily fish intake || id:ukb-b-2209	rs9301837	460443	-0.01574	0.00273	8.10E-09	A	C	0.143273	33.2426395
Oily fish intake || id:ukb-b-2209	rs4982738	460443	0.010861	0.001969	3.50E-08	A	G	0.582899	30.4139906
Oily fish intake || id:ukb-b-2209	rs12896749	460443	-0.01096	0.001966	2.50E-08	C	G	0.384721	31.09911448
Oily fish intake || id:ukb-b-2209	rs1951286	460443	-0.01458	0.001999	3.00E-13	G	T	0.644911	53.19581795
Oily fish intake || id:ukb-b-2209	rs28533540	460443	0.014642	0.001925	2.80E-14	A	G	0.534203	57.86880877
Oily fish intake || id:ukb-b-2209	rs1421085	460443	0.018481	0.001949	2.50E-21	C	T	0.40341	89.94279752
Oily fish intake || id:ukb-b-2209	rs11859365	460443	0.022567	0.002197	9.40E-25	C	A	0.253765	105.5277608
Oily fish intake || id:ukb-b-2209	rs9889161	460443	-0.01332	0.002002	2.80E-11	T	G	0.357929	44.28980638
Oily fish intake || id:ukb-b-2209	rs28623270	460443	-0.01783	0.002737	7.30E-11	T	A	0.14872	42.43688656
Oily fish intake || id:ukb-b-2209	rs2952140	460443	-0.01068	0.001915	2.50E-08	T	C	0.482607	31.0867504
Oily fish intake || id:ukb-b-2209	rs4510068	460443	-0.01301	0.001971	4.00E-11	T	G	0.402798	43.60078526
Oily fish intake || id:ukb-b-2209	rs7243428	460443	-0.01298	0.002292	1.50E-08	G	A	0.224648	32.06030201
Oily fish intake || id:ukb-b-2209	rs9958909	460443	0.015762	0.002778	1.40E-08	G	T	0.139718	32.18944523
Oily fish intake || id:ukb-b-2209	rs59355765	460443	-0.01625	0.00261	4.70E-10	T	C	0.160079	38.78434474
Oily fish intake || id:ukb-b-2209	rs4002471	460443	-0.01924	0.001924	1.50E-23	T	C	0.547362	99.99896072
Oily fish intake || id:ukb-b-2209	rs7254235	460443	-0.01063	0.00194	4.30E-08	G	A	0.577254	30.02216639
Oily fish intake || id:ukb-b-2209	rs75887709	460443	-0.01591	0.002815	1.60E-08	G	A	0.13588	31.95052732
Oily fish intake || id:ukb-b-2209	rs12983532	460443	-0.01339	0.002233	2.00E-09	T	C	0.251137	35.94371149
Oily fish intake || id:ukb-b-2209	rs6033437	460443	0.012468	0.00221	1.70E-08	A	C	0.257329	31.83613518
Oily fish intake || id:ukb-b-2209	rs6059844	460443	0.011	0.001915	9.20E-09	G	A	0.495121	33.01246762
Oily fish intake || id:ukb-b-2209	rs6089753	460443	-0.01154	0.001918	1.80E-09	T	C	0.530967	36.19793605
Oily fish intake || id:ukb-b-2209	rs2827161	460443	0.010711	0.001937	3.20E-08	G	T	0.422842	30.57370271
Oily fish intake || id:ukb-b-2209	rs9606833	460443	0.016986	0.002231	2.70E-14	C	T	0.243593	57.97132923
Pork intake || id:ukb-b-5640	rs11211124	460162	-0.00995	0.001754	1.40E-08	C	T	0.230603	32.19479408
Pork intake || id:ukb-b-5640	rs9973426	460162	0.011085	0.001937	1.00E-08	G	A	0.176598	32.75573161
Pork intake || id:ukb-b-5640	rs7641973	460162	0.008446	0.001541	4.20E-08	A	G	0.353362	30.04202564
Pork intake || id:ukb-b-5640	rs254152	460162	-0.01043	0.001742	2.20E-09	G	C	0.23498	35.81825003
Pork intake || id:ukb-b-5640	rs9379832	460162	-0.01147	0.001706	1.80E-11	G	A	0.255452	45.200144
Pork intake || id:ukb-b-5640	rs10972033	460162	0.008976	0.001479	1.30E-09	T	G	0.45643	36.85322886
Pork intake || id:ukb-b-5640	rs1355171	460162	-0.01098	0.001477	1.00E-13	A	C	0.488844	55.29639177
Pork intake || id:ukb-b-5640	rs34161520	460162	0.011592	0.002021	9.60E-09	G	C	0.160381	32.91156654
Pork intake || id:ukb-b-5640	rs2387807	460162	-0.01508	0.002748	4.10E-08	T	C	0.077893	30.12431625
Pork intake || id:ukb-b-5640	rs4146837	460162	0.008792	0.001494	4.00E-09	T	C	0.455561	34.61523839

Pork intake || id:ukb-b-5640	rs3964074	460162	-0.00895	0.001483	1.60E-09	C	T	0.546932	36.40632524
Pork intake || id:ukb-b-5640	rs36124222	460162	0.008409	0.001501	2.10E-08	C	T	0.433239	31.39509867
Pork intake || id:ukb-b-5640	rs12721051	460162	-0.01236	0.001887	5.60E-11	G	C	0.188393	42.94470595
Pork intake || id:ukb-b-5640	rs838133	460162	0.010894	0.001525	9.00E-13	G	A	0.549354	51.0408865
Poultry intake || id:ukb-b-8006	rs9997448	461900	-0.01046	0.001883	2.70E-08	T	C	0.369236	30.88872486
Poultry intake || id:ukb-b-8006	rs7829800	461900	0.011458	0.001943	3.70E-09	G	A	0.670759	34.78147808
Poultry intake || id:ukb-b-8006	rs7046351	461900	0.011075	0.001817	1.10E-09	A	T	0.509739	37.13120108
Poultry intake || id:ukb-b-8006	rs1051730	461900	-0.01088	0.001929	1.70E-08	A	G	0.331381	31.77633978
Poultry intake || id:ukb-b-8006	rs9923768	461900	0.010512	0.001859	1.60E-08	A	G	0.59853	31.97727011
Poultry intake || id:ukb-b-8006	rs2565017	461900	0.010952	0.001882	5.90E-09	A	G	0.372697	33.85768114
Poultry intake || id:ukb-b-8006	rs2965200	461900	-0.01042	0.001901	4.20E-08	A	G	0.640011	30.04667768
Poultry intake || id:ukb-b-8006	rs2426440	461900	0.011218	0.002053	4.70E-08	G	A	0.732602	29.84918243
Processed meat intake || id:ukb-b-6324	rs7531118	461981	-0.01407	0.002113	2.80E-11	C	T	0.531133	44.33158973
Processed meat intake || id:ukb-b-6324	rs77165542	461981	0.033879	0.005725	3.30E-09	T	C	0.035496	35.01844797
Processed meat intake || id:ukb-b-6324	rs11887120	461981	0.011962	0.00216	3.10E-08	T	C	0.397683	30.65733918
Processed meat intake || id:ukb-b-6324	rs11894162	461981	0.012049	0.002107	1.10E-08	T	C	0.547464	32.70197435
Processed meat intake || id:ukb-b-6324	rs4077924	461981	0.012499	0.002285	4.50E-08	C	T	0.701892	29.92808497
Processed meat intake || id:ukb-b-6324	rs3762621	461981	-0.01499	0.00272	3.60E-08	T	C	0.183451	30.37723627
Processed meat intake || id:ukb-b-6324	rs9809856	461981	0.013289	0.0021	2.50E-10	G	A	0.475881	40.05556924
Processed meat intake || id:ukb-b-6324	rs2873054	461981	0.013996	0.00219	1.60E-10	C	A	0.353276	40.84724346
Processed meat intake || id:ukb-b-6324	rs6786550	461981	0.012187	0.002174	2.10E-08	C	T	0.635043	31.42314922
Processed meat intake || id:ukb-b-6324	rs6765179	461981	-0.01276	0.002266	1.80E-08	A	G	0.309968	31.7087382
Processed meat intake || id:ukb-b-6324	rs10454812	461981	-0.01996	0.003442	6.70E-09	C	A	0.103032	33.62616394
Processed meat intake || id:ukb-b-6324	rs2029401	461981	0.014631	0.002129	6.30E-12	G	A	0.586212	47.2201083
Processed meat intake || id:ukb-b-6324	rs1422192	461981	0.016968	0.002871	3.40E-09	A	G	0.158076	34.92330511
Processed meat intake || id:ukb-b-6324	rs6961970	461981	-0.01401	0.002441	9.50E-09	A	C	0.244698	32.95106191
Processed meat intake || id:ukb-b-6324	rs4240672	461981	0.01712	0.002094	3.00E-16	A	G	0.494014	66.83584837
Processed meat intake || id:ukb-b-6324	rs6484504	461981	0.015473	0.002348	4.40E-11	C	T	0.72462	43.42888028
Processed meat intake || id:ukb-b-6324	rs11032380	461981	-0.01332	0.002223	2.10E-09	T	A	0.333352	35.92015049
Processed meat intake || id:ukb-b-6324	rs4778053	461981	0.016467	0.002899	1.30E-08	G	C	0.843808	32.2701224
Processed meat intake || id:ukb-b-6324	rs34241936	461981	0.032837	0.005753	1.10E-08	G	A	0.037369	32.57424823
Processed meat intake || id:ukb-b-6324	rs8096167	461981	-0.01459	0.002671	4.70E-08	C	T	0.1926	29.85623806
Processed meat intake || id:ukb-b-6324	rs838133	461981	0.019014	0.002166	1.60E-18	G	A	0.549369	77.08978661
Processed meat intake || id:ukb-b-6324	rs6010651	461981	-0.01241	0.002168	1.10E-08	C	A	0.379439	32.74568067
Processed meat intake || id:ukb-b-6324	rs203319	461981	-0.01642	0.002602	2.80E-10	T	C	0.204594	39.84163356
Salad / raw vegetable intake || id:ukb-b-1996	rs9427220	435435	-0.00801	0.001442	2.80E-08	T	A	0.554661	30.83517474
Salad / raw vegetable intake || id:ukb-b-1996	rs4083969	435435	0.017133	0.003115	3.80E-08	G	C	0.057217	30.24846399
Salad / raw vegetable intake || id:ukb-b-1996	rs7619139	435435	0.012477	0.001451	8.00E-18	A	T	0.589193	73.94937827
Salad / raw vegetable intake || id:ukb-b-1996	rs13102393	435435	0.007979	0.00143	2.40E-08	G	C	0.499077	31.12322902
Salad / raw vegetable intake || id:ukb-b-1996	rs17460017	435435	0.011174	0.001813	7.20E-10	T	A	0.190124	37.97263927
Salad / raw vegetable intake || id:ukb-b-1996	rs2194027	435435	-0.00861	0.001436	2.00E-09	A	T	0.484664	35.95028361
Salad / raw vegetable intake || id:ukb-b-1996	rs3129962	435435	-0.0133	0.002123	3.70E-10	A	G	0.129168	39.28062216
Salad / raw vegetable intake || id:ukb-b-1996	rs12203592	435435	-0.01029	0.001694	1.30E-09	T	C	0.219338	36.86605923
Salad / raw vegetable intake || id:ukb-b-1996	rs3095337	435435	-0.01262	0.001766	9.00E-13	C	G	0.203909	51.05981399
Salad / raw vegetable intake || id:ukb-b-1996	rs75248709	435435	-0.01973	0.003528	2.20E-08	T	C	0.045947	31.29195574
Salad / raw vegetable intake || id:ukb-b-1996	rs57221424	435435	0.008941	0.001533	5.50E-09	G	C	0.321733	34.00446016
Salad / raw vegetable intake || id:ukb-b-1996	rs62461186	435435	-0.01134	0.001857	1.00E-09	C	A	0.179827	37.30299064
Salad / raw vegetable intake || id:ukb-b-1996	rs790561	435435	0.012474	0.001562	1.40E-15	G	A	0.704134	63.77566285
Salad / raw vegetable intake || id:ukb-b-1996	rs7821179	435435	-0.01082	0.001976	4.40E-08	C	G	0.846577	29.96117219
Salad / raw vegetable intake || id:ukb-b-1996	rs10819082	435435	-0.00916	0.001513	1.40E-09	A	G	0.66734	36.67096236
Salad / raw vegetable intake || id:ukb-b-1996	rs6482190	435435	0.011272	0.00159	1.40E-12	G	A	0.719408	50.2415982
Salad / raw vegetable intake || id:ukb-b-1996	rs1890012	435435	-0.01043	0.001808	8.10E-09	G	T	0.19473	33.25452525
Salad / raw vegetable intake || id:ukb-b-1996	rs12908495	435435	-0.00935	0.001666	2.00E-08	A	C	0.242533	31.50750448
Salad / raw vegetable intake || id:ukb-b-1996	rs1052352	435435	0.008164	0.001426	1.00E-08	T	C	0.52355	32.79691991
Salad / raw vegetable intake || id:ukb-b-1996	rs34186148	435435	-0.00805	0.001475	4.80E-08	C	G	0.370054	29.80024351
Salad / raw vegetable intake || id:ukb-b-1996	rs4291983	435435	-0.0084	0.001425	3.70E-09	A	C	0.517576	34.75594909
Salad / raw vegetable intake || id:ukb-b-1996	rs8130508	435435	0.00874	0.001578	3.00E-08	A	G	0.289686	30.68069068
Salt added to food || id:ukb-b-8121	rs12563932	462630	-0.02849	0.00521	4.50E-08	C	A	0.029478	29.90918161
Salt added to food || id:ukb-b-8121	rs10752999	462630	0.012762	0.001927	3.50E-11	C	A	0.702035	43.85373858
Salt added to food || id:ukb-b-8121	rs12094804	462630	0.020506	0.003656	2.00E-08	G	A	0.061754	31.46264204
Salt added to food || id:ukb-b-8121	rs11210985	462630	-0.01566	0.001818	7.20E-18	A	G	0.3791	74.16054984
Salt added to food || id:ukb-b-8121	rs6695915	462630	-0.01663	0.002998	2.90E-08	G	A	0.095551	30.75941412
Salt added to food || id:ukb-b-8121	rs1008078	462630	0.01166	0.001806	1.10E-10	T	C	0.395512	41.65850007
Salt added to food || id:ukb-b-8121	rs528301	462630	0.015261	0.00177	6.60E-18	A	G	0.554846	74.3317749
Salt added to food || id:ukb-b-8121	rs976179	462630	0.010067	0.001765	1.20E-08	T	A	0.485324	32.53725369
Salt added to food || id:ukb-b-8121	rs55897719	462630	0.013502	0.001896	1.10E-12	A	C	0.316817	50.71377907
Salt added to food || id:ukb-b-8121	rs7591518	462630	-0.02077	0.00193	5.10E-27	C	T	0.298453	115.8653479
Salt added to food || id:ukb-b-8121	rs11126666	462630	-0.01199	0.002019	2.90E-09	A	G	0.255562	35.25162725
Salt added to food || id:ukb-b-8121	rs2263636	462630	0.011256	0.001965	1.00E-08	C	A	0.719001	32.82698401
Salt added to food || id:ukb-b-8121	rs13400612	462630	0.020285	0.002043	3.00E-23	G	C	0.246906	98.62800776
Salt added to food || id:ukb-b-8121	rs12988960	462630	0.014981	0.001896	2.80E-15	C	G	0.315972	62.40138572
Salt added to food || id:ukb-b-8121	rs264932	462630	-0.01042	0.001802	7.30E-09	G	A	0.605889	33.44367058
Salt added to food || id:ukb-b-8121	rs7581335	462630	0.014931	0.002533	3.80E-09	T	A	0.140518	34.74126996
Salt added to food || id:ukb-b-8121	rs17805497	462630	0.012482	0.00185	1.50E-11	C	T	0.345071	45.51036228
Salt added to food || id:ukb-b-8121	rs13084934	462630	-0.00968	0.001767	4.40E-08	T	A	0.494691	29.969865
Salt added to food || id:ukb-b-8121	rs6776248	462630	0.011341	0.001998	1.40E-08	C	T	0.26336	32.21977471
Salt added to food || id:ukb-b-8121	rs6780346	462630	-0.01684	0.001817	1.90E-20	T	C	0.620997	85.84503183
Salt added to food || id:ukb-b-8121	rs400750	462630	0.016712	0.001815	3.30E-20	G	T	0.616712	84.8128261
Salt added to food || id:ukb-b-8121	rs2736748	462630	0.028012	0.00216	1.80E-38	G	A	0.789193	168.2486097
Salt added to food || id:ukb-b-8121	rs73040343	462630	-0.01035	0.001814	1.20E-08	G	A	0.381434	32.5544108
Salt added to food || id:ukb-b-8121	rs736935	462630	0.015688	0.001879	6.80E-17	T	C	0.336413	69.72802014
Salt added to food || id:ukb-b-8121	rs9843358	462630	0.018953	0.002344	6.20E-16	T	C	0.171903	65.36627414
Salt added to food || id:ukb-b-8121	rs9835772	462630	0.011451	0.002052	2.40E-08	T	A	0.243664	31.13738978
Salt added to food || id:ukb-b-8121	rs11130206	462630	-0.01203	0.001776	1.20E-11	G	C	0.432321	45.9249289
Salt added to food || id:ukb-b-8121	rs6804929	462630	0.016706	0.002889	7.40E-09	A	G	0.105016	33.43330753
Salt added to food || id:ukb-b-8121	rs6443950	462630	-0.01421	0.001831	8.50E-15	T	A	0.631504	60.21326612
Salt added to food || id:ukb-b-8121	rs7673170	462630	-0.0126	0.001801	2.70E-12	A	G	0.395273	48.90204364

Salt added to food || id:ukb-b-8121	rs12501838	462630	0.014667	0.002082	1.80E-12	T	A	0.23366	49.64778409
Salt added to food || id:ukb-b-8121	rs7670308	462630	-0.01164	0.001801	1.00E-10	A	G	0.409195	41.75946011
Salt added to food || id:ukb-b-8121	rs4860797	462630	0.011667	0.001808	1.10E-10	A	G	0.601276	41.65808626
Salt added to food || id:ukb-b-8121	rs34906832	462630	0.015098	0.00253	2.40E-09	G	A	0.140896	35.60826027
Salt added to food || id:ukb-b-8121	rs526210	462630	0.010095	0.001819	2.80E-08	A	G	0.542868	30.8095771
Salt added to food || id:ukb-b-8121	rs3890316	462630	-0.01133	0.002042	2.80E-08	A	G	0.249954	30.81064487
Salt added to food || id:ukb-b-8121	rs13131880	462630	-0.01262	0.002128	3.00E-09	C	T	0.220413	35.18221896
Salt added to food || id:ukb-b-8121	rs12658060	462630	-0.02741	0.002118	2.50E-38	C	T	0.222971	167.5963664
Salt added to food || id:ukb-b-8121	rs868720	462630	0.015994	0.001925	9.60E-17	C	G	0.305451	69.05358891
Salt added to food || id:ukb-b-8121	rs33137	462630	-0.01208	0.001773	9.40E-12	T	C	0.493871	46.44332372
Salt added to food || id:ukb-b-8121	rs1823011	462630	-0.01023	0.001861	3.90E-08	G	A	0.659939	30.18540582
Salt added to food || id:ukb-b-8121	rs4912891	462630	0.010655	0.001923	3.00E-08	C	T	0.691882	30.70249555
Salt added to food || id:ukb-b-8121	rs4235642	462630	0.012294	0.001816	1.30E-11	G	A	0.379691	45.80406063
Salt added to food || id:ukb-b-8121	rs6887291	462630	0.012676	0.001836	5.10E-12	G	T	0.636126	47.63945323
Salt added to food || id:ukb-b-8121	rs2339234	462630	-0.01764	0.0019	1.60E-20	A	G	0.683186	86.20494285
Salt added to food || id:ukb-b-8121	rs9375448	462630	-0.01168	0.001762	3.40E-11	T	A	0.492981	43.95057289
Salt added to food || id:ukb-b-8121	rs35099536	462630	0.025959	0.003207	5.80E-16	C	A	0.08461	65.49908856
Salt added to food || id:ukb-b-8121	rs4236065	462630	-0.01117	0.002031	3.80E-08	C	T	0.255885	30.26706717
Salt added to food || id:ukb-b-8121	rs28366169	462630	-0.02085	0.003299	2.60E-10	A	G	0.077319	39.92541372
Salt added to food || id:ukb-b-8121	rs2506738	462630	-0.0151	0.001801	5.00E-17	G	A	0.401141	70.33413718
Salt added to food || id:ukb-b-8121	rs9278020	462630	0.023222	0.002862	4.80E-16	A	G	0.10619	65.85915985
Salt added to food || id:ukb-b-8121	rs2463710	462630	0.011952	0.002191	4.90E-08	A	T	0.753885	29.75383129
Salt added to food || id:ukb-b-8121	rs1726866	462630	0.026544	0.00177	7.70E-51	A	G	0.552077	224.8983154
Salt added to food || id:ukb-b-8121	rs11761254	462630	-0.01768	0.002546	3.80E-12	T	C	0.136978	48.2298397
Salt added to food || id:ukb-b-8121	rs2457427	462630	-0.01658	0.002569	1.10E-10	C	T	0.863856	41.64764359
Salt added to food || id:ukb-b-8121	rs6987313	462630	0.009907	0.001766	2.00E-08	C	T	0.520156	31.47780687
Salt added to food || id:ukb-b-8121	rs4739105	462630	0.015465	0.00218	1.30E-12	C	T	0.787781	50.3180536
Salt added to food || id:ukb-b-8121	rs10971930	462630	0.015501	0.002644	4.50E-09	C	T	0.127418	34.38315493
Salt added to food || id:ukb-b-8121	rs586716	462630	0.014367	0.001793	1.10E-15	A	G	0.414681	64.18088129
Salt added to food || id:ukb-b-8121	rs7465705	462630	-0.01185	0.001816	6.80E-11	A	G	0.384538	42.56725616
Salt added to food || id:ukb-b-8121	rs7021360	462630	-0.01012	0.00181	2.30E-08	A	C	0.388225	31.25260398
Salt added to food || id:ukb-b-8121	rs10128297	462630	-0.01055	0.0018	4.50E-09	T	C	0.402787	34.38236171
Salt added to food || id:ukb-b-8121	rs10883796	462630	0.011441	0.001934	3.30E-09	A	G	0.294358	34.98459757
Salt added to food || id:ukb-b-8121	rs4595499	462630	-0.01728	0.001808	1.20E-21	T	C	0.391888	91.34708069
Salt added to food || id:ukb-b-8121	rs4948275	462630	0.012715	0.001773	7.40E-13	T	C	0.481851	51.44692403
Salt added to food || id:ukb-b-8121	rs10736951	462630	-0.01136	0.001843	7.00E-10	C	G	0.635537	38.00926553
Salt added to food || id:ukb-b-8121	rs9667150	462630	0.012589	0.001781	1.50E-12	A	G	0.559523	49.98615471
Salt added to food || id:ukb-b-8121	rs329670	462630	-0.01529	0.002633	6.30E-09	C	T	0.870289	33.73501715
Salt added to food || id:ukb-b-8121	rs35271178	462630	0.010244	0.001791	1.10E-08	T	C	0.588507	32.71763603
Salt added to food || id:ukb-b-8121	rs7927679	462630	0.014954	0.001764	2.30E-17	T	C	0.504024	71.86569511
Salt added to food || id:ukb-b-8121	rs12789951	462630	-0.01172	0.001797	7.00E-11	T	C	0.411036	42.52020593
Salt added to food || id:ukb-b-8121	rs7110845	462630	-0.01154	0.001786	1.10E-10	G	A	0.579174	41.71927452
Salt added to food || id:ukb-b-8121	rs35702851	462630	0.012196	0.002191	2.60E-08	C	G	0.204203	30.99853126
Salt added to food || id:ukb-b-8121	rs11022746	462630	0.011827	0.001913	6.30E-10	G	T	0.681941	38.21516977
Salt added to food || id:ukb-b-8121	rs99780	462630	0.010907	0.001848	3.60E-09	T	C	0.350239	34.85112376
Salt added to food || id:ukb-b-8121	rs667128	462630	0.015426	0.002652	6.00E-09	T	C	0.126584	33.82515219
Salt added to food || id:ukb-b-8121	rs1895951	462630	-0.01169	0.002142	4.80E-08	C	T	0.220794	29.78591831
Salt added to food || id:ukb-b-8121	rs324018	462630	0.01001	0.001812	3.30E-08	G	T	0.613528	30.53313421
Salt added to food || id:ukb-b-8121	rs12579997	462630	0.016031	0.002348	8.60E-12	C	G	0.170455	46.62146884
Salt added to food || id:ukb-b-8121	rs1045411	462630	-0.01507	0.001993	4.00E-14	T	C	0.268305	57.17062891
Salt added to food || id:ukb-b-8121	rs9569747	462630	-0.01256	0.001936	8.60E-11	G	T	0.298073	42.10585185
Salt added to food || id:ukb-b-8121	rs9317406	462630	-0.0107	0.001824	4.40E-09	T	C	0.376115	34.42832064
Salt added to food || id:ukb-b-8121	rs7982263	462630	0.010608	0.001788	2.90E-09	C	T	0.581981	35.22148582
Salt added to food || id:ukb-b-8121	rs10140751	462630	-0.01127	0.002047	3.60E-08	T	G	0.245951	30.34374832
Salt added to food || id:ukb-b-8121	rs4981196	462630	-0.01146	0.001822	3.30E-10	C	A	0.635898	39.51373198
Salt added to food || id:ukb-b-8121	rs8022455	462630	-0.01114	0.001774	3.40E-10	C	T	0.54702	39.44578346
Salt added to food || id:ukb-b-8121	rs2693687	462630	0.011628	0.00182	1.70E-10	T	C	0.393736	40.79768528
Salt added to food || id:ukb-b-8121	rs961044	462630	-0.01401	0.002527	3.00E-08	T	C	0.857621	30.72064854
Salt added to food || id:ukb-b-8121	rs2521501	462630	-0.0114	0.0019	2.00E-09	T	A	0.322086	35.95619075
Salt added to food || id:ukb-b-8121	rs8040685	462630	-0.01541	0.002758	2.30E-08	T	C	0.884465	31.20392241
Salt added to food || id:ukb-b-8121	rs491907	462630	0.010986	0.001763	4.60E-10	G	A	0.511555	38.83452439
Salt added to food || id:ukb-b-8121	rs6416794	462630	-0.012	0.0022	4.90E-08	C	T	0.798724	29.74170034
Salt added to food || id:ukb-b-8121	rs1728779	462630	0.009871	0.001789	3.50E-08	G	A	0.436092	30.43583114
Salt added to food || id:ukb-b-8121	rs11075194	462630	-0.00982	0.001787	4.00E-08	G	A	0.425797	30.16236733
Salt added to food || id:ukb-b-8121	rs2547040	462630	-0.01271	0.002229	1.20E-08	C	G	0.805074	32.49178461
Salt added to food || id:ukb-b-8121	rs72807804	462630	0.012064	0.002139	1.70E-08	T	C	0.218782	31.81063573
Salt added to food || id:ukb-b-8121	rs35142265	462630	0.015013	0.002136	2.10E-12	G	A	0.218202	49.38614983
Salt added to food || id:ukb-b-8121	rs11082431	462630	0.010565	0.001921	3.80E-08	T	C	0.301904	30.25466782
Salt added to food || id:ukb-b-8121	rs62098445	462630	-0.01586	0.001911	1.00E-16	A	C	0.318657	68.87986577
Salt added to food || id:ukb-b-8121	rs8097544	462630	0.01403	0.002513	2.40E-08	G	A	0.145366	31.17563684
Salt added to food || id:ukb-b-8121	rs4799949	462630	-0.01098	0.001872	4.50E-09	T	C	0.667226	34.39990489
Salt added to food || id:ukb-b-8121	rs2852348	462630	-0.01312	0.001777	1.60E-13	G	A	0.458696	54.4601483
Salt added to food || id:ukb-b-8121	rs429358	462630	-0.0154	0.002442	2.90E-10	C	T	0.154168	39.75827215
Salt added to food || id:ukb-b-8121	rs2835623	462630	0.022677	0.004144	4.40E-08	T	C	0.04766	29.95112178
Salt added to food || id:ukb-b-8121	rs9611875	462630	0.066201	0.004519	1.30E-48	G	A	0.039616	214.635715
Salt added to food || id:ukb-b-8121	rs2899345	462630	0.009887	0.001773	2.50E-08	C	T	0.504298	31.09436749
Water intake || id:ukb-b-14898	rs34967813	427588	-0.01206	0.001956	7.10E-10	G	A	0.311025	38.00302747
Water intake || id:ukb-b-14898	rs2305813	427588	-0.01912	0.002769	5.00E-12	C	G	0.123354	47.66951406
Water intake || id:ukb-b-14898	rs782221	427588	-0.01271	0.002262	1.90E-08	T	C	0.200429	31.58096512
Water intake || id:ukb-b-14898	rs182050989	427588	0.030528	0.005474	2.40E-08	T	C	0.02835	31.10316429
Water intake || id:ukb-b-14898	rs11890994	427588	0.011095	0.001879	3.60E-09	T	A	0.371683	34.85067295
Water intake || id:ukb-b-14898	rs2198234	427588	0.01167	0.001816	1.30E-10	T	G	0.528757	41.30033818
Water intake || id:ukb-b-14898	rs11125629	427588	0.010867	0.001823	2.50E-09	G	A	0.546761	35.53872078
Water intake || id:ukb-b-14898	rs9830293	427588	0.019082	0.003475	4.00E-08	G	A	0.073462	30.15829689
Water intake || id:ukb-b-14898	rs7626335	427588	0.010769	0.001933	2.50E-08	C	A	0.668446	31.04722459
Water intake || id:ukb-b-14898	rs6844845	427588	-0.02151	0.003894	3.30E-08	G	A	0.057487	30.5230641
Water intake || id:ukb-b-14898	rs6835325	427588	-0.01187	0.001978	2.00E-09	G	T	0.30096	35.97622437

Water intake || id:ukb-b-14898	rs67174962	427588	-0.01226	0.002157	1.30E-08	A	G	0.232324	32.34092507
Water intake || id:ukb-b-14898	rs6905712	427588	0.011088	0.001928	8.90E-09	A	T	0.664977	33.06715227
Water intake || id:ukb-b-14898	rs2274156	427588	-0.01149	0.002071	2.90E-08	T	C	0.258771	30.76619403
Water intake || id:ukb-b-14898	rs6957745	427588	-0.01529	0.002262	1.40E-11	C	T	0.202686	45.69599112
Water intake || id:ukb-b-14898	rs3808058	427588	0.018332	0.002816	7.50E-11	T	C	0.117807	42.37345884
Water intake || id:ukb-b-14898	rs4410790	427588	-0.03087	0.001877	8.60E-61	C	T	0.629877	270.5421948
Water intake || id:ukb-b-14898	rs10954732	427588	0.01062	0.001928	3.60E-08	A	G	0.671297	30.33775273
Water intake || id:ukb-b-14898	rs2656285	427588	0.012242	0.002007	1.10E-09	C	T	0.709918	37.19674487
Water intake || id:ukb-b-14898	rs9414686	427588	0.01476	0.002406	8.60E-10	T	C	0.178007	37.62204636
Water intake || id:ukb-b-14898	rs10758255	427588	-0.01079	0.00186	6.70E-09	A	T	0.609855	33.61739575
Water intake || id:ukb-b-14898	rs11140831	427588	-0.01238	0.001836	1.50E-11	G	A	0.514151	45.47717791
Water intake || id:ukb-b-14898	rs35028442	427588	0.016424	0.002688	9.90E-10	C	G	0.137272	37.33602984
Water intake || id:ukb-b-14898	rs11012726	427588	-0.01224	0.001979	6.20E-10	C	T	0.307334	38.24414741
Water intake || id:ukb-b-14898	rs3763874	427588	0.01633	0.00184	6.90E-19	A	G	0.41763	78.79441688
Water intake || id:ukb-b-14898	rs7124005	427588	0.010367	0.00186	2.50E-08	T	C	0.415348	31.04995536
Water intake || id:ukb-b-14898	rs2229357	427588	-0.01221	0.002115	7.90E-09	A	G	0.240393	33.30314151
Water intake || id:ukb-b-14898	rs34940743	427588	-0.01039	0.001898	4.40E-08	G	A	0.346783	29.96484877
Water intake || id:ukb-b-14898	rs146394874	427588	0.026142	0.00422	5.80E-10	A	C	0.049323	38.37927245
Water intake || id:ukb-b-14898	rs1963510	427588	0.013307	0.00182	2.60E-13	G	A	0.458107	53.48637183
Water intake || id:ukb-b-14898	rs4603502	427588	-0.01307	0.001993	5.40E-11	C	T	0.292378	43.0259953
Water intake || id:ukb-b-14898	rs2472297	427588	-0.03342	0.002057	2.40E-59	T	C	0.26061	263.9073012
Water intake || id:ukb-b-14898	rs8054636	427588	-0.01033	0.001851	2.40E-08	C	T	0.537476	31.14808957
Water intake || id:ukb-b-14898	rs2289292	427588	-0.01277	0.001927	3.40E-11	T	C	0.334337	43.90867628
Water intake || id:ukb-b-14898	rs1421085	427588	0.01201	0.001846	7.70E-11	C	T	0.403711	42.33483863
Water intake || id:ukb-b-14898	rs2435200	427588	0.011214	0.001838	1.10E-09	A	G	0.412694	37.20774372
Water intake || id:ukb-b-14898	rs4239466	427588	0.010643	0.001896	2.00E-08	C	A	0.391897	31.49257013
Water intake || id:ukb-b-14898	rs9957088	427588	-0.01183	0.002087	1.40E-08	T	C	0.253192	32.12392725
Water intake || id:ukb-b-14898	rs429358	427588	0.016681	0.002509	3.00E-11	C	T	0.154368	44.2019162
Water intake || id:ukb-b-14898	rs56100328	427588	-0.01755	0.003217	4.80E-08	G	A	0.090196	29.78130102
Water intake || id:ukb-b-14898	rs3746410	427588	0.016839	0.00225	7.30E-14	G	A	0.20396	55.99847645
Hot drink temperature || id:ukb-b-14203	rs79564740	457873	-0.01925	0.003453	2.50E-08	C	T	0.030417	31.08358054
Hot drink temperature || id:ukb-b-14203	rs2786529	457873	-0.00831	0.001456	1.20E-08	G	C	0.209494	32.55132125
Hot drink temperature || id:ukb-b-14203	rs12132579	457873	0.007053	0.001238	1.20E-08	T	C	0.357498	32.43585083
Hot drink temperature || id:ukb-b-14203	rs10927006	457873	-0.01374	0.001693	4.80E-16	C	T	0.143611	65.88046637
Hot drink temperature || id:ukb-b-14203	rs12038134	457873	0.006537	0.001187	3.60E-08	A	T	0.508213	30.35154638
Hot drink temperature || id:ukb-b-14203	rs1001880	457873	0.007384	0.001284	8.90E-09	C	T	0.313764	33.06738103
Hot drink temperature || id:ukb-b-14203	rs12622811	457873	-0.01026	0.001302	3.10E-15	T	C	0.298571	62.17992726
Hot drink temperature || id:ukb-b-14203	rs74384251	457873	0.009525	0.001727	3.50E-08	T	C	0.136886	30.43161005
Hot drink temperature || id:ukb-b-14203	rs62134736	457873	0.007577	0.001351	2.00E-08	A	G	0.2591	31.45238438
Hot drink temperature || id:ukb-b-14203	rs17409597	457873	-0.00699	0.001194	4.90E-09	C	T	0.491526	34.2191888
Hot drink temperature || id:ukb-b-14203	rs1260326	457873	-0.00919	0.00121	3.20E-14	C	T	0.604332	57.59371483
Hot drink temperature || id:ukb-b-14203	rs1568452	457873	-0.01205	0.001222	5.90E-23	T	C	0.382005	97.32815252
Hot drink temperature || id:ukb-b-14203	rs17024335	457873	-0.00829	0.001431	6.90E-09	C	T	0.223585	33.5667636
Hot drink temperature || id:ukb-b-14203	rs6736362	457873	-0.00665	0.001195	2.70E-08	T	C	0.560656	30.93425193
Hot drink temperature || id:ukb-b-14203	rs1997468	457873	0.010862	0.001194	9.40E-20	T	C	0.439876	82.7289413
Hot drink temperature || id:ukb-b-14203	rs34292254	457873	0.007443	0.001195	4.70E-10	T	G	0.552492	38.81751032
Hot drink temperature || id:ukb-b-14203	rs1144428	457873	0.012221	0.00146	5.80E-17	A	G	0.790277	70.02982548
Hot drink temperature || id:ukb-b-14203	rs9835772	457873	-0.00808	0.001381	4.90E-09	T	A	0.243662	34.24505228
Hot drink temperature || id:ukb-b-14203	rs12695358	457873	-0.00706	0.00121	5.40E-09	T	G	0.597342	34.04426384
Hot drink temperature || id:ukb-b-14203	rs888405	457873	0.009196	0.001468	3.80E-10	G	A	0.794793	39.22013877
Hot drink temperature || id:ukb-b-14203	rs17023019	457873	0.012121	0.001242	1.70E-22	G	A	0.647408	95.24613315
Hot drink temperature || id:ukb-b-14203	rs11710570	457873	-0.00768	0.001192	1.20E-10	C	T	0.448108	41.53538335
Hot drink temperature || id:ukb-b-14203	rs1513475	457873	-0.00891	0.001244	7.80E-13	C	T	0.34937	51.34250586
Hot drink temperature || id:ukb-b-14203	rs2255015	457873	-0.00698	0.001192	4.70E-09	A	G	0.454555	34.29084504
Hot drink temperature || id:ukb-b-14203	rs2702576	457873	0.007416	0.001224	1.40E-09	G	A	0.621311	36.73459649
Hot drink temperature || id:ukb-b-14203	rs2952894	457873	0.0072	0.001258	1.00E-08	C	T	0.664339	32.76689754
Hot drink temperature || id:ukb-b-14203	rs6876382	457873	-0.01514	0.002524	2.00E-09	G	C	0.05895	35.97775683
Hot drink temperature || id:ukb-b-14203	rs111251222	457873	-0.00773	0.00136	1.30E-08	G	A	0.258804	32.340478
Hot drink temperature || id:ukb-b-14203	rs2728784	457873	-0.01101	0.001463	5.30E-14	G	A	0.208554	56.63337995
Hot drink temperature || id:ukb-b-14203	rs6892119	457873	-0.00807	0.00122	3.90E-11	G	A	0.574513	43.68683053
Hot drink temperature || id:ukb-b-14203	rs6469	457873	0.010933	0.001931	1.50E-08	T	C	0.127478	32.05461809
Hot drink temperature || id:ukb-b-14203	rs1326609	457873	-0.00781	0.001228	2.10E-10	A	G	0.371579	40.3905707
Hot drink temperature || id:ukb-b-14203	rs12664031	457873	-0.01045	0.001376	3.00E-14	A	C	0.289569	57.71417917
Hot drink temperature || id:ukb-b-14203	rs3132487	457873	-0.00965	0.00128	4.80E-14	T	G	0.345911	56.79464766
Hot drink temperature || id:ukb-b-14203	rs9372734	457873	-0.00802	0.001188	1.40E-11	T	C	0.482495	45.60284033
Hot drink temperature || id:ukb-b-14203	rs62403110	457873	-0.00743	0.00129	8.40E-09	A	G	0.302512	33.18868793
Hot drink temperature || id:ukb-b-14203	rs3117103	457873	-0.01526	0.001707	4.00E-19	T	A	0.139554	79.89262948
Hot drink temperature || id:ukb-b-14203	rs58391518	457873	-0.00737	0.001307	1.70E-08	C	T	0.291369	31.79826863
Hot drink temperature || id:ukb-b-14203	rs4410790	457873	-0.01096	0.001229	4.80E-19	C	T	0.630852	79.50706537
Hot drink temperature || id:ukb-b-14203	rs2113336	457873	-0.00721	0.001192	1.40E-09	A	C	0.470426	36.60539892
Hot drink temperature || id:ukb-b-14203	rs35968894	457873	-0.00722	0.001225	3.80E-09	G	A	0.377461	34.71953421
Hot drink temperature || id:ukb-b-14203	rs210600	457873	0.007675	0.001326	7.10E-09	A	G	0.27676	33.50258837
Hot drink temperature || id:ukb-b-14203	rs2360802	457873	-0.00942	0.001423	3.60E-11	T	A	0.225125	43.8358507
Hot drink temperature || id:ukb-b-14203	rs62572325	457873	0.011211	0.001739	1.20E-10	A	C	0.13467	41.53914692
Hot drink temperature || id:ukb-b-14203	rs62580693	457873	0.008041	0.001367	4.10E-09	C	G	0.252484	34.58186543
Hot drink temperature || id:ukb-b-14203	rs1027583	457873	-0.00766	0.001344	1.20E-08	C	G	0.272924	32.50465369
Hot drink temperature || id:ukb-b-14203	rs10822089	457873	-0.00728	0.001188	8.90E-10	G	A	0.523143	37.55909285
Hot drink temperature || id:ukb-b-14203	rs10764990	457873	0.009482	0.001214	5.80E-15	A	G	0.607319	60.96180986
Hot drink temperature || id:ukb-b-14203	rs10829603	457873	0.007357	0.001195	7.50E-10	G	T	0.451828	37.87986576
Hot drink temperature || id:ukb-b-14203	rs17461712	457873	0.012089	0.001595	3.40E-14	C	T	0.167426	57.46297641
Hot drink temperature || id:ukb-b-14203	rs11570094	457873	0.007546	0.001302	6.80E-09	A	C	0.295801	33.59042568
Hot drink temperature || id:ukb-b-14203	rs4492837	457873	-0.00679	0.001206	1.80E-08	C	G	0.586887	31.71251389
Hot drink temperature || id:ukb-b-14203	rs1447182	457873	0.009392	0.001451	9.50E-11	C	T	0.782248	41.91942528
Hot drink temperature || id:ukb-b-14203	rs61909866	457873	-0.00734	0.001277	8.90E-09	T	C	0.316065	33.06403153
Hot drink temperature || id:ukb-b-14203	rs2712661	457873	-0.00824	0.001409	5.10E-09	A	G	0.769898	34.16220827
Hot drink temperature || id:ukb-b-14203	rs10744560	457873	-0.00689	0.001254	3.90E-08	T	C	0.340057	30.19418932
Hot drink temperature || id:ukb-b-14203	rs826848	457873	-0.01478	0.002388	6.10E-10	T	C	0.933713	38.30242227

Hot drink temperature || id:ukb-b-14203	rs4477562	457873	-0.01047	0.001786	4.50E-09	T	C	0.1287	34.38656641
Hot drink temperature || id:ukb-b-14203	rs9570736	457873	-0.00656	0.001187	3.30E-08	G	A	0.518982	30.49624368
Hot drink temperature || id:ukb-b-14203	rs34759521	457873	-0.00826	0.001417	5.60E-09	A	T	0.227087	33.97207095
Hot drink temperature || id:ukb-b-14203	rs2472297	457873	-0.01539	0.001345	2.60E-30	T	C	0.261771	130.9128068
Hot drink temperature || id:ukb-b-14203	rs34935263	457873	-0.00682	0.00119	1.00E-08	G	A	0.533192	32.82318419
Hot drink temperature || id:ukb-b-14203	rs58726064	457873	0.006771	0.001196	1.50E-08	G	C	0.442086	32.06326818
Hot drink temperature || id:ukb-b-14203	rs55880962	457873	0.008487	0.001505	1.70E-08	A	G	0.192325	31.7875179
Hot drink temperature || id:ukb-b-14203	rs17513240	457873	-0.01065	0.001569	1.20E-11	T	A	0.172466	46.04594926
Hot drink temperature || id:ukb-b-14203	rs4452038	457873	-0.01118	0.001223	6.50E-20	T	C	0.389867	83.45611928
Hot drink temperature || id:ukb-b-14203	rs1132845	457873	-0.00699	0.001213	8.20E-09	T	C	0.404377	33.23506008
Hot drink temperature || id:ukb-b-14203	rs1680349	457873	-0.00673	0.001198	1.90E-08	A	G	0.563781	31.57143105
Hot drink temperature || id:ukb-b-14203	rs6045331	457873	-0.00759	0.001227	6.10E-10	G	C	0.37918	38.28236343
Hot drink temperature || id:ukb-b-14203	rs2825972	457873	0.008751	0.001304	1.90E-11	G	A	0.292758	45.04134557
Hot drink temperature || id:ukb-b-14203	rs2032512	457873	-0.00892	0.001215	2.10E-13	C	A	0.607796	53.89209135
Tea intake || id:ukb-b-6066	rs11587444	447485	0.014033	0.002171	1.00E-10	G	A	0.393464	41.78852554
Tea intake || id:ukb-b-6066	rs11164870	447485	-0.01196	0.002182	4.20E-08	G	C	0.604574	30.03686088
Tea intake || id:ukb-b-6066	rs56188862	447485	-0.01576	0.002175	4.30E-13	C	T	0.387454	52.49734455
Tea intake || id:ukb-b-6066	rs1156588	447485	-0.01545	0.002603	2.90E-09	G	A	0.210071	35.24114916
Tea intake || id:ukb-b-6066	rs57462170	447485	0.019151	0.003406	1.90E-08	A	G	0.108773	31.62025072
Tea intake || id:ukb-b-6066	rs2117137	447485	0.012995	0.002156	1.70E-09	G	A	0.405148	36.33812848
Tea intake || id:ukb-b-6066	rs1481012	447485	-0.02624	0.003356	5.30E-15	G	A	0.112209	61.14752739
Tea intake || id:ukb-b-6066	rs34619	447485	0.011712	0.002138	4.30E-08	A	G	0.430905	30.02117124
Tea intake || id:ukb-b-6066	rs72797284	447485	-0.01711	0.002384	7.00E-13	G	A	0.270797	51.55815132
Tea intake || id:ukb-b-6066	rs7757102	447485	-0.0118	0.002133	3.10E-08	G	A	0.555426	30.62394866
Tea intake || id:ukb-b-6066	rs2478875	447485	0.021894	0.002611	5.10E-17	G	A	0.208758	70.29944849
Tea intake || id:ukb-b-6066	rs149805207	447485	-0.07193	0.012582	1.10E-08	G	A	0.008538	32.68471685
Tea intake || id:ukb-b-6066	rs4410790	447485	0.040551	0.002195	3.40E-76	C	T	0.631224	341.2697706
Tea intake || id:ukb-b-6066	rs17685	447485	0.023066	0.002362	1.60E-22	A	G	0.277512	95.36397544
Tea intake || id:ukb-b-6066	rs141071726	447485	0.040732	0.006812	2.20E-09	A	G	0.026713	35.75355444
Tea intake || id:ukb-b-6066	rs9648476	447485	0.012501	0.002185	1.10E-08	A	G	0.622954	32.72205182
Tea intake || id:ukb-b-6066	rs713598	447485	0.013397	0.002157	5.20E-10	G	C	0.402254	38.58988258
Tea intake || id:ukb-b-6066	rs13282783	447485	-0.01358	0.002354	7.90E-09	T	C	0.285899	33.28933955
Tea intake || id:ukb-b-6066	rs56348300	447485	0.015882	0.002732	6.10E-09	G	C	0.184619	33.79866009
Tea intake || id:ukb-b-6066	rs10764990	447485	-0.01219	0.002169	1.90E-08	A	G	0.607155	31.58923203
Tea intake || id:ukb-b-6066	rs10752269	447485	-0.01287	0.00212	1.30E-09	A	G	0.506082	36.87822488
Tea intake || id:ukb-b-6066	rs2351187	447485	0.012902	0.002282	1.60E-08	A	G	0.318935	31.95867836
Tea intake || id:ukb-b-6066	rs17245213	447485	-0.01465	0.002609	2.00E-08	A	G	0.208046	31.52084042
Tea intake || id:ukb-b-6066	rs10741694	447485	0.015004	0.002194	7.90E-12	C	T	0.627915	46.78446497
Tea intake || id:ukb-b-6066	rs1453548	447485	-0.01334	0.00225	3.00E-09	A	T	0.664929	35.16754172
Tea intake || id:ukb-b-6066	rs977474	447485	0.021781	0.002856	2.40E-14	T	C	0.833746	58.18029093
Tea intake || id:ukb-b-6066	rs2783129	447485	-0.01173	0.002133	3.80E-08	G	C	0.484878	30.25428023
Tea intake || id:ukb-b-6066	rs17576658	447485	-0.01348	0.002457	4.10E-08	A	G	0.247081	30.11659629
Tea intake || id:ukb-b-6066	rs6829	447485	-0.01192	0.002165	3.70E-08	T	C	0.596155	30.28185843
Tea intake || id:ukb-b-6066	rs2645929	447485	-0.01498	0.002717	3.50E-08	G	A	0.813066	30.42395917
Tea intake || id:ukb-b-6066	rs12591786	447485	-0.01844	0.002942	3.70E-10	T	C	0.158804	39.27397356
Tea intake || id:ukb-b-6066	rs2472297	447485	0.053345	0.002401	2.30E-109	T	C	0.262049	493.6455641
Tea intake || id:ukb-b-6066	rs9937354	447485	-0.01409	0.002143	4.90E-11	A	G	0.424074	43.23125388
Tea intake || id:ukb-b-6066	rs9302428	447485	0.012246	0.002201	2.60E-08	G	C	0.635799	30.94855178
Tea intake || id:ukb-b-6066	rs2279844	447485	-0.01199	0.002183	4.00E-08	A	G	0.379343	30.15137588
Tea intake || id:ukb-b-6066	rs4808193	447485	0.015115	0.002247	1.70E-11	C	T	0.335324	45.24046958
Tea intake || id:ukb-b-6066	rs57631352	447485	-0.0131	0.002321	1.70E-08	G	A	0.296859	31.86843439
Tea intake || id:ukb-b-6066	rs2273447	447485	0.017472	0.002634	3.30E-11	T	A	0.203788	43.99057077
Tea intake || id:ukb-b-6066	rs4817505	447485	0.015068	0.002175	4.20E-12	C	T	0.38998	48.01229387
Tea intake || id:ukb-b-6066	rs132904	447485	0.016601	0.002553	7.80E-11	C	G	0.778651	42.29582311
Tea intake || id:ukb-b-6066	rs9624470	447485	0.025207	0.002155	1.30E-31	A	G	0.580054	136.8395635
Alcohol intake frequency. || id:ukb-b-5779	rs780569	462346	0.019803	0.003365	4.00E-09	A	T	0.70882	34.64365792
Alcohol intake frequency. || id:ukb-b-5779	rs4503294	462346	0.018148	0.00307	3.40E-09	T	C	0.565333	34.93196544
Alcohol intake frequency. || id:ukb-b-5779	rs28787109	462346	0.017811	0.003085	7.70E-09	A	G	0.40423	33.33968197
Alcohol intake frequency. || id:ukb-b-5779	rs2244598	462346	-0.01838	0.003119	3.80E-09	C	T	0.605114	34.7173247
Alcohol intake frequency. || id:ukb-b-5779	rs4417025	462346	-0.01884	0.003165	2.70E-09	A	G	0.361153	35.42204553
Alcohol intake frequency. || id:ukb-b-5779	rs7514579	462346	0.019667	0.003598	4.60E-08	C	A	0.232457	29.88076555
Alcohol intake frequency. || id:ukb-b-5779	rs2717063	462346	-0.02037	0.003085	4.00E-11	A	C	0.585731	43.61238661
Alcohol intake frequency. || id:ukb-b-5779	rs6727281	462346	-0.02432	0.00392	5.50E-10	T	C	0.184023	38.5040317
Alcohol intake frequency. || id:ukb-b-5779	rs780094	462346	-0.05099	0.003105	1.30E-60	C	T	0.615206	269.7085781
Alcohol intake frequency. || id:ukb-b-5779	rs13390019	462346	0.029612	0.004492	4.30E-11	C	T	0.134041	43.458933
Alcohol intake frequency. || id:ukb-b-5779	rs10188314	462346	-0.01979	0.003036	7.20E-11	T	C	0.470852	42.47541969
Alcohol intake frequency. || id:ukb-b-5779	rs4241258	462346	0.025064	0.004403	1.30E-08	T	C	0.13763	32.3996422
Alcohol intake frequency. || id:ukb-b-5779	rs72769229	462346	-0.02314	0.004192	3.40E-08	T	A	0.154942	30.464867
Alcohol intake frequency. || id:ukb-b-5779	rs17662759	462346	0.030135	0.00546	3.40E-08	C	T	0.089115	30.45804524
Alcohol intake frequency. || id:ukb-b-5779	rs1991083	462346	-0.02239	0.003258	6.30E-12	T	C	0.679886	47.23548167
Alcohol intake frequency. || id:ukb-b-5779	rs473098	462346	-0.02174	0.003043	9.10E-13	T	C	0.557689	51.02825402
Alcohol intake frequency. || id:ukb-b-5779	rs9829192	462346	0.016932	0.00305	2.80E-08	T	G	0.435133	30.81386917
Alcohol intake frequency. || id:ukb-b-5779	rs76082653	462346	0.046427	0.006687	3.80E-12	T	C	0.054327	48.2071878
Alcohol intake frequency. || id:ukb-b-5779	rs262240	462346	-0.01721	0.003035	1.40E-08	T	C	0.468553	32.14708366
Alcohol intake frequency. || id:ukb-b-5779	rs9814516	462346	-0.02511	0.003556	1.60E-12	T	G	0.237423	49.87186315
Alcohol intake frequency. || id:ukb-b-5779	rs7610856	462346	-0.02386	0.00307	7.70E-15	A	C	0.429053	60.41537671
Alcohol intake frequency. || id:ukb-b-5779	rs1515591	462346	0.01823	0.003116	4.90E-09	G	T	0.383186	34.22119536
Alcohol intake frequency. || id:ukb-b-5779	rs1228589	462346	0.02107	0.003528	2.30E-09	A	G	0.246133	35.66588658
Alcohol intake frequency. || id:ukb-b-5779	rs28622224	462346	-0.01862	0.003368	3.20E-08	T	C	0.280364	30.55916633
Alcohol intake frequency. || id:ukb-b-5779	rs13135092	462346	0.043834	0.005499	1.60E-15	G	A	0.083483	63.54509331
Alcohol intake frequency. || id:ukb-b-5779	rs11940694	462346	-0.04371	0.003116	1.00E-44	G	A	0.604193	196.7963941
Alcohol intake frequency. || id:ukb-b-5779	rs362307	462346	0.043305	0.005802	8.40E-14	T	C	0.074582	55.70397358
Alcohol intake frequency. || id:ukb-b-5779	rs1229984	462346	-0.26171	0.009185	1.40E-178	C	T	0.97277	811.8568483
Alcohol intake frequency. || id:ukb-b-5779	rs13102973	462346	-0.01941	0.003119	4.90E-10	C	T	0.61881	38.72261133
Alcohol intake frequency. || id:ukb-b-5779	rs62339673	462346	0.018294	0.003154	6.60E-09	A	C	0.626705	33.64276576
Alcohol intake frequency. || id:ukb-b-5779	rs34811474	462346	-0.02018	0.003593	1.90E-08	A	G	0.230728	31.54674383
Alcohol intake frequency. || id:ukb-b-5779	rs2159935	462346	-0.01857	0.003026	8.30E-10	A	G	0.490369	37.68200968

Alcohol intake frequency. || id:ukb-b-5779	rs62305780	462346	-0.04852	0.005066	9.90E-22	G	C	0.102253	91.74144456
Alcohol intake frequency. || id:ukb-b-5779	rs13178443	462346	-0.01865	0.00339	3.80E-08	T	C	0.276349	30.27445602
Alcohol intake frequency. || id:ukb-b-5779	rs11750777	462346	-0.02049	0.003726	3.80E-08	A	G	0.209454	30.24788927
Alcohol intake frequency. || id:ukb-b-5779	rs4916723	462346	0.023948	0.0031	1.10E-14	C	A	0.420617	59.69648887
Alcohol intake frequency. || id:ukb-b-5779	rs461599	462346	-0.01919	0.00304	2.70E-10	C	A	0.462259	39.84870236
Alcohol intake frequency. || id:ukb-b-5779	rs56194430	462346	0.02254	0.004071	3.10E-08	T	C	0.16931	30.64889155
Alcohol intake frequency. || id:ukb-b-5779	rs9403297	462346	0.018823	0.00313	1.80E-09	A	G	0.372967	36.15803299
Alcohol intake frequency. || id:ukb-b-5779	rs9349379	462346	-0.01935	0.003082	3.50E-10	G	A	0.405493	39.39602243
Alcohol intake frequency. || id:ukb-b-5779	rs12153855	462346	0.029444	0.004935	2.40E-09	C	T	0.10497	35.59858335
Alcohol intake frequency. || id:ukb-b-5779	rs9372625	462346	-0.02556	0.003125	2.90E-16	A	G	0.381706	66.9024493
Alcohol intake frequency. || id:ukb-b-5779	rs62466318	462346	-0.02549	0.003774	1.40E-11	T	C	0.202827	45.6199276
Alcohol intake frequency. || id:ukb-b-5779	rs2622167	462346	-0.01912	0.003067	4.60E-10	A	G	0.428653	38.83564281
Alcohol intake frequency. || id:ukb-b-5779	rs73050128	462346	-0.026	0.004091	2.10E-10	A	C	0.164488	40.40857686
Alcohol intake frequency. || id:ukb-b-5779	rs6943160	462346	0.020627	0.003728	3.10E-08	C	T	0.208646	30.6182884
Alcohol intake frequency. || id:ukb-b-5779	rs4726481	462346	0.021761	0.003102	2.30E-12	T	G	0.400576	49.21796788
Alcohol intake frequency. || id:ukb-b-5779	rs9648478	462346	0.01686	0.003029	2.60E-08	A	G	0.510245	30.98545356
Alcohol intake frequency. || id:ukb-b-5779	rs2160935	462346	-0.01872	0.003091	1.40E-09	T	C	0.604293	36.66680814
Alcohol intake frequency. || id:ukb-b-5779	rs34440851	462346	-0.02268	0.004151	4.60E-08	T	C	0.157151	29.8666998
Alcohol intake frequency. || id:ukb-b-5779	rs11787216	462346	0.024416	0.003201	2.40E-14	T	C	0.369127	58.18948292
Alcohol intake frequency. || id:ukb-b-5779	rs2977454	462346	-0.02592	0.004599	1.70E-08	G	C	0.124072	31.77647113
Alcohol intake frequency. || id:ukb-b-5779	rs74679146	462346	-0.03207	0.005758	2.50E-08	C	T	0.074515	31.03232076
Alcohol intake frequency. || id:ukb-b-5779	rs489062	462346	0.01665	0.003053	4.90E-08	A	G	0.437454	29.74336079
Alcohol intake frequency. || id:ukb-b-5779	rs34473884	462346	-0.02036	0.003503	6.20E-09	A	G	0.24819	33.77732173
Alcohol intake frequency. || id:ukb-b-5779	rs61873510	462346	0.020374	0.003303	6.90E-10	T	G	0.32785	38.0446629
Alcohol intake frequency. || id:ukb-b-5779	rs4242715	462346	-0.01865	0.003248	9.30E-09	A	G	0.680585	32.97941711
Alcohol intake frequency. || id:ukb-b-5779	rs10792669	462346	0.017432	0.003041	9.90E-09	G	A	0.505254	32.86786802
Alcohol intake frequency. || id:ukb-b-5779	rs11223617	462346	0.025091	0.003754	2.30E-11	A	G	0.206155	44.6770453
Alcohol intake frequency. || id:ukb-b-5779	rs550942	462346	0.022401	0.003989	2.00E-08	T	C	0.823865	31.53853961
Alcohol intake frequency. || id:ukb-b-5779	rs11039429	462346	-0.02356	0.003037	8.70E-15	T	C	0.454624	60.16400522
Alcohol intake frequency. || id:ukb-b-5779	rs1666658	462346	0.017967	0.003099	6.70E-09	C	T	0.392206	33.62192855
Alcohol intake frequency. || id:ukb-b-5779	rs12312693	462346	-0.01768	0.00305	6.80E-09	C	T	0.451772	33.60043049
Alcohol intake frequency. || id:ukb-b-5779	rs7302200	462346	-0.01842	0.003198	8.40E-09	A	G	0.339998	33.17528913
Alcohol intake frequency. || id:ukb-b-5779	rs28768122	462346	0.0207	0.003552	5.60E-09	C	T	0.759525	33.96135389
Alcohol intake frequency. || id:ukb-b-5779	rs7298932	462346	-0.02372	0.004312	3.80E-08	G	A	0.147849	30.26855022
Alcohol intake frequency. || id:ukb-b-5779	rs58905411	462346	-0.02663	0.003078	5.10E-18	A	G	0.410052	74.85311902
Alcohol intake frequency. || id:ukb-b-5779	rs1937522	462346	0.016898	0.003032	2.50E-08	G	A	0.528054	31.05896823
Alcohol intake frequency. || id:ukb-b-5779	rs7330939	462346	-0.02133	0.003405	3.70E-10	T	C	0.720352	39.25125918
Alcohol intake frequency. || id:ukb-b-5779	rs2535911	462346	-0.01885	0.003168	2.70E-09	T	C	0.354749	35.3840411
Alcohol intake frequency. || id:ukb-b-5779	rs186347	462346	0.017949	0.003051	4.00E-09	T	G	0.463343	34.61575629
Alcohol intake frequency. || id:ukb-b-5779	rs80292319	462346	-0.03937	0.006496	1.40E-09	C	T	0.057704	36.73865902
Alcohol intake frequency. || id:ukb-b-5779	rs117799466	462346	-0.01967	0.00332	3.10E-09	C	G	0.336989	35.11025138
Alcohol intake frequency. || id:ukb-b-5779	rs34631026	462346	-0.01691	0.003048	2.90E-08	T	C	0.446061	30.78292721
Alcohol intake frequency. || id:ukb-b-5779	rs72787062	462346	-0.02819	0.004103	6.40E-12	A	G	0.162767	47.21512202
Alcohol intake frequency. || id:ukb-b-5779	rs35105141	462346	0.026345	0.003088	1.40E-17	T	C	0.401541	72.79064649
Alcohol intake frequency. || id:ukb-b-5779	rs1421085	462346	0.019939	0.003085	1.00E-10	C	T	0.403447	41.77905563
Alcohol intake frequency. || id:ukb-b-5779	rs1104608	462346	0.017421	0.003088	1.70E-08	C	G	0.426338	31.81733013
Alcohol intake frequency. || id:ukb-b-5779	rs8043563	462346	0.023365	0.003471	1.70E-11	C	G	0.737192	45.30797529
Alcohol intake frequency. || id:ukb-b-5779	rs2411453	462346	-0.03508	0.00309	7.30E-30	G	T	0.597353	128.8472807
Alcohol intake frequency. || id:ukb-b-5779	rs728538	462346	0.022875	0.004063	1.80E-08	G	T	0.168868	31.70471177
Alcohol intake frequency. || id:ukb-b-5779	rs9906502	462346	0.023788	0.003962	1.90E-09	A	G	0.176998	36.05338973
Alcohol intake frequency. || id:ukb-b-5779	rs8614	462346	0.024781	0.003925	2.70E-10	A	C	0.182509	39.85313011
Alcohol intake frequency. || id:ukb-b-5779	rs4968391	462346	-0.01927	0.003227	2.30E-09	T	G	0.674892	35.66790102
Alcohol intake frequency. || id:ukb-b-5779	rs9912298	462346	0.020589	0.00359	9.70E-09	C	A	0.239585	32.89390404
Alcohol intake frequency. || id:ukb-b-5779	rs17690703	462346	0.025034	0.00343	2.90E-13	T	C	0.262687	53.26304827
Alcohol intake frequency. || id:ukb-b-5779	rs650558	462346	0.020736	0.003508	3.40E-09	T	C	0.247918	34.94330164
Alcohol intake frequency. || id:ukb-b-5779	rs1893659	462346	-0.02933	0.003053	7.60E-22	A	C	0.459939	92.2603435
Alcohol intake frequency. || id:ukb-b-5779	rs5022348	462346	0.020264	0.00357	1.40E-08	T	C	0.40703	32.21855383
Alcohol intake frequency. || id:ukb-b-5779	rs2043677	462346	0.026113	0.004327	1.60E-09	T	C	0.145599	36.41745392
Alcohol intake frequency. || id:ukb-b-5779	rs9958320	462346	0.024855	0.004271	5.90E-09	C	T	0.153147	33.86843628
Alcohol intake frequency. || id:ukb-b-5779	rs62097995	462346	0.020002	0.003067	6.90E-11	A	T	0.423591	42.53903583
Alcohol intake frequency. || id:ukb-b-5779	rs2924321	462346	-0.01951	0.00305	1.60E-10	A	G	0.539592	40.92514357
Alcohol intake frequency. || id:ukb-b-5779	rs4940926	462346	-0.0191	0.003441	2.80E-08	C	T	0.735045	30.81710309
Alcohol intake frequency. || id:ukb-b-5779	rs838145	462346	0.021955	0.003055	6.70E-13	A	G	0.542982	51.6294189
Alcohol intake frequency. || id:ukb-b-5779	rs6030200	462346	-0.01953	0.003271	2.40E-09	A	G	0.31415	35.65047418
Alcohol intake frequency. || id:ukb-b-5779	rs11700855	462346	-0.0298	0.005233	1.20E-08	G	A	0.093465	32.41892344
Alcohol intake frequency. || id:ukb-b-5779	rs71651683	462346	-0.07046	0.012791	3.60E-08	T	C	0.0142	30.3451915
Alcohol intake frequency. || id:ukb-b-5779	rs1894544	462346	0.017393	0.003046	1.10E-08	C	G	0.454379	32.60071975

Table S3:SNPs removed due to associated with outcome or confounders

trait	SNP	samplesize.exposure	beta.exposure	se.exposure	pval.exposure	effect_allele.exposure	other_allele.exposure	eaf.exposure
Years of educational attainment in females	rs1008078	462630	0.01166	0.001806	1.10E-10	T	C	0.395512
Years of educational attainment	rs10140751	462630	-0.01127	0.002047	3.60E-08	T	G	0.245951
Years of educational attainment in females	rs10156602	448651	0.011006	0.001637	1.80E-11	G	A	0.361369
Ever smoked	rs10161952	448651	-0.00958	0.001686	1.30E-08	C	A	0.31274
Alcohol intake frequency	rs10188314	462346	-0.01979	0.003036	7.20E-11	T	C	0.470852
Ever smoked	rs10192394	446462	-0.00766	0.001229	4.50E-10	T	C	0.528785
Ever smoked	rs1051730	461900	-0.01088	0.001929	1.70E-08	A	G	0.331381
Years of educational attainment in females	rs1073242	451486	0.015728	0.002292	6.70E-12	A	G	0.553825
Usual walking pace	rs10740991	421764	0.016739	0.001857	2.00E-19	C	G	0.717606
Usual walking pace	rs10828250	460443	-0.02015	0.002073	2.60E-22	G	C	0.309332
Usual walking pace	rs10828266	446462	0.012364	0.001357	8.10E-20	G	A	0.715634
Usual walking pace	rs10838724	446462	0.009011	0.001283	2.10E-12	T	G	0.367992
Years of educational attainment	rs10883796	462630	0.011441	0.001934	3.30E-09	A	G	0.294358
Time spent watching television	rs10896050	451486	-0.01847	0.002834	7.20E-11	T	G	0.193171
Usual walking pace	rs10927006	457873	-0.01374	0.001693	4.80E-16	C	T	0.143611
Usual walking pace	rs11012726	427588	-0.01224	0.001979	6.20E-10	C	T	0.307334
Usual walking pace	rs11039429	462346	-0.02356	0.003037	8.70E-15	T	C	0.454624
Alcohol intake frequency	rs1104608	462346	0.017421	0.003088	1.70E-08	C	G	0.426338
Alcohol intake frequency	rs1104608	441640	0.010863	0.001818	2.30E-09	C	G	0.426239
Years of educational attainment in females	rs11130206	462630	-0.01203	0.001776	1.20E-11	G	C	0.432321
Alcohol intake frequency	rs11223617	462346	0.025091	0.003754	2.30E-11	A	G	0.206155
Usual walking pace	rs11570094	457873	0.007546	0.001302	6.80E-09	A	C	0.295801
Years of educational attainment in females	rs11586016	421764	0.009878	0.00173	1.10E-08	C	G	0.371004
Alcohol intake frequency	rs11649653	451486	0.013849	0.002292	1.50E-09	G	C	0.38208
Alcohol intake frequency	rs11700855	462346	-0.0298	0.005233	1.20E-08	G	A	0.093465
Years of educational attainment in females	rs11743441	460006	-0.00884	0.001486	2.70E-09	T	G	0.574343
Years of educational attainment	rs11772627	421764	0.018334	0.002171	3.00E-17	C	G	0.18202
Alcohol intake frequency	rs117799466	462346	-0.01967	0.00332	3.10E-09	C	G	0.336989
Years of educational attainment in females	rs11787216	462346	0.024416	0.003201	2.40E-14	T	C	0.369127
Years of educational attainment in females	rs11811826	421764	0.013218	0.002006	4.40E-11	A	T	0.224231
Alcohol intake frequency	rs11940694	462346	-0.04371	0.003116	1.00E-44	G	A	0.604193
Alcohol intake frequency	rs11940694	441640	-0.01266	0.001834	5.00E-12	G	A	0.604068
Years of educational attainment in females	rs12044599	446462	0.009421	0.001503	3.70E-10	G	A	0.21008
Alcohol intake frequency	rs12153855	462346	0.029444	0.004935	2.40E-09	C	T	0.10497
Years of educational attainment in males	rs12247907	461053	0.009841	0.001711	8.90E-09	C	G	0.485752
Alcohol intake frequency	rs1228589	462346	0.02107	0.003528	2.30E-09	A	G	0.246133
Alcohol intake frequency	rs1229984	462346	-0.26171	0.009185	1.40E-178	C	T	0.97277
Alcohol intake frequency	rs12312693	462346	-0.01768	0.00305	6.80E-09	C	T	0.451772
Years of educational attainment in males	rs12354267	441640	0.011647	0.001933	1.70E-09	C	T	0.309145
Alcohol intake frequency	rs1260326	460880	-0.00955	0.001655	7.90E-09	C	T	0.604249
Alcohol intake frequency	rs1260326	457873	-0.00919	0.00121	3.20E-14	C	T	0.604332
Ever smoked	rs12855717	460443	-0.01223	0.001923	2.00E-10	T	C	0.526774
Alcohol intake frequency	rs1291145	451486	-0.02025	0.00241	4.40E-17	C	T	0.685848
Alcohol intake frequency	rs13070166	460443	0.014211	0.002278	4.40E-10	A	T	0.228586
Alcohol intake frequency	rs13102973	462346	-0.01941	0.003119	4.90E-10	C	T	0.61881
Usual walking pace	rs13107325	451486	-0.02916	0.004253	7.00E-12	T	C	0.074693
Usual walking pace	rs13135092	462346	0.043834	0.005499	1.60E-15	G	A	0.083483
Years of educational attainment	rs13163336	428860	0.014947	0.00221	1.30E-11	A	C	0.15761
Alcohol intake frequency	rs13178443	462346	-0.01865	0.00339	3.80E-08	T	C	0.276349
Alcohol intake frequency	rs13234131	441640	0.017012	0.00266	1.60E-10	G	A	0.128369
Years of educational attainment in females	rs1338549	428860	-0.00945	0.001622	5.60E-09	G	T	0.533932
Alcohol intake frequency	rs13390019	462346	0.029612	0.004492	4.30E-11	C	T	0.134041
Alcohol intake frequency	rs1375566	446462	-0.00783	0.001266	6.10E-10	A	G	0.627412
Usual walking pace	rs1421085	460443	0.018481	0.001949	2.50E-21	C	T	0.40341
Usual walking pace	rs1421085	428860	0.018543	0.001644	1.70E-29	C	T	0.40357
Usual walking pace	rs1421085	461053	-0.01212	0.001743	3.50E-12	C	T	0.403433
Usual walking pace	rs1421085	448651	0.010332	0.001591	8.30E-11	C	T	0.40346
Usual walking pace	rs1421085	462346	0.019939	0.003085	1.00E-10	C	T	0.403447
Usual walking pace	rs1421085	427588	0.01201	0.001846	7.70E-11	C	T	0.403711
Years of educational attainment	rs1422192	461981	0.016968	0.002871	3.40E-09	A	G	0.158076
Ever smoked	rs1453548	447485	-0.01334	0.00225	3.00E-09	A	T	0.664929
Coffee consumption cups per day	rs1481012	447485	-0.02624	0.003356	5.30E-15	G	A	0.112209
Usual walking pace	rs1513475	457873	-0.00891	0.001244	7.80E-13	C	T	0.34937
Alcohol intake frequency	rs1515591	462346	0.01823	0.003116	4.90E-09	G	T	0.383186
Years of educational attainment in females	rs1620977	446462	-0.01317	0.001378	1.10E-21	G	A	0.730517
Alcohol intake frequency	rs1666658	462346	0.017967	0.003099	6.70E-09	C	T	0.392206
Alcohol intake frequency	rs1680349	457873	-0.00673	0.001198	1.90E-08	A	G	0.563781
Years of educational attainment	rs16822430	460880	0.011634	0.00192	1.40E-09	C	T	0.23321
Ever smoked	rs16891727	460443	-0.02372	0.00284	6.80E-17	A	C	0.129814

Alcohol intake frequency	rs17023019	457873	0.012121	0.001242	1.70E-22	G	A	0.647408
Alcohol intake frequency	rs17175518	421764	0.011496	0.001975	5.90E-09	A	C	0.232775
Alcohol intake frequency	rs1726866	462630	0.026544	0.00177	7.70E-51	A	G	0.552077
Ever smoked	rs17409597	457873	-0.00699	0.001194	4.90E-09	C	T	0.491526
Alcohol intake frequency	rs17662759	462346	0.030135	0.00546	3.40E-08	C	T	0.089115
Coffee consumption	rs17685	447485	0.023066	0.002362	1.60E-22	A	G	0.277512
Years of educational attainment	rs17690703	462346	0.025034	0.00343	2.90E-13	T	C	0.262687
Alcohol intake frequency	rs17842490	428860	-0.04517	0.006808	3.30E-11	G	A	0.014248
Alcohol intake frequency	rs186347	462346	0.017949	0.003051	4.00E-09	T	G	0.463343
Years of educational attainment	rs1876245	460443	0.015116	0.001931	5.00E-15	C	T	0.431482
Alcohol intake frequency	rs1894544	462346	0.017393	0.003046	1.10E-08	C	G	0.454379
Alcohol intake frequency	rs1991083	462346	-0.02239	0.003258	6.30E-12	T	C	0.679886
Alcohol intake frequency	rs2043677	462346	0.026113	0.004327	1.60E-09	T	C	0.145599
Years of educational attainment	rs2048522	446462	0.009563	0.001248	1.80E-14	T	A	0.434968
Alcohol intake frequency	rs2159935	462346	-0.01857	0.003026	8.30E-10	A	G	0.490369
Alcohol intake frequency	rs2160935	462346	-0.01872	0.003091	1.40E-09	T	C	0.604293
Years of educational attainment	rs2189234	428860	0.009987	0.001661	1.80E-09	G	T	0.617795
Alcohol intake frequency	rs2194027	435435	-0.00861	0.001436	2.00E-09	A	T	0.484664
Ever smoked	rs2198234	427588	0.01167	0.001816	1.30E-10	T	G	0.528757
Alcohol intake frequency	rs2244598	462346	-0.01838	0.003119	3.80E-09	C	T	0.605114
Time spent watching television	rs2252508	448651	0.009102	0.001562	5.70E-09	G	A	0.480256
Years of educational attainment in females	rs2352974	451486	-0.0145	0.002243	1.00E-10	T	C	0.48979
Usual walking pace	rs2411453	462346	-0.03508	0.00309	7.30E-30	G	T	0.597353
Coffee consumption	rs2472297	457873	-0.01539	0.001345	2.60E-30	T	C	0.261771
Coffee consumption	rs2472297	428860	0.046471	0.001827	1.10E-142	T	C	0.262883
Coffee consumption	rs2472297	427588	-0.03342	0.002057	2.40E-59	T	C	0.26061
Coffee consumption	rs2472297	447485	0.053345	0.002401	2.30E-109	T	C	0.262049
Coffee consumption	rs2472297	441640	-0.01585	0.002022	4.50E-15	T	C	0.261458
Alcohol intake frequency	rs2535911	462346	-0.01885	0.003168	2.70E-09	T	C	0.354749
Alcohol intake frequency	rs2622167	462346	-0.01912	0.003067	4.60E-10	A	G	0.428653
Alcohol intake frequency	rs262240	462346	-0.01721	0.003035	1.40E-08	T	C	0.468553
Ever smoked	rs264932	462630	-0.01042	0.001802	7.30E-09	G	A	0.605889
Years of educational attainment in females	rs26579	451486	-0.0128	0.002294	2.40E-08	C	G	0.586217
Alcohol intake frequency	rs2717063	462346	-0.02037	0.003085	4.00E-11	A	C	0.585731
Alcohol intake frequency	rs2726033	460006	-0.00949	0.00148	1.50E-10	G	A	0.422384
Years of educational attainment	rs2728784	457873	-0.01101	0.001463	5.30E-14	G	A	0.208554
Years of educational attainment in females	rs2736748	462630	0.028012	0.00216	1.80E-38	G	A	0.789193
Ever smoked	rs2799849	441640	-0.01233	0.001906	9.80E-11	T	C	0.678123
Years of educational attainment in males	rs2802530	451486	0.018627	0.003397	4.20E-08	A	G	0.876502
Alcohol intake frequency	rs28479795	446462	0.011232	0.001473	2.50E-14	T	C	0.221473
Years of educational attainment in females	rs28768122	462346	0.0207	0.003552	5.60E-09	C	T	0.759525
Alcohol intake frequency	rs2924321	462346	-0.01951	0.00305	1.60E-10	A	G	0.539592
Years of educational attainment	rs2960578	451486	0.017028	0.002236	2.60E-14	G	T	0.496281
Ever smoked	rs3095337	435435	-0.01262	0.001766	9.00E-13	C	G	0.203909
Years of educational attainment	rs3101339	421764	0.01426	0.001705	6.20E-17	C	A	0.603285
Alcohol intake frequency	rs34060476	428860	0.018429	0.00237	7.50E-15	G	A	0.133855
Alcohol intake frequency	rs34161520	460162	0.011592	0.002021	9.60E-09	G	C	0.160381
Years of educational attainment	rs34186148	435435	-0.00805	0.001475	4.80E-08	C	G	0.370054
Years of educational attainment	rs34198643	451486	-0.0167	0.002679	4.50E-10	T	C	0.224165
Years of educational attainment	rs34473884	462346	-0.02036	0.003503	6.20E-09	A	G	0.24819
Years of educational attainment in females	rs34619	447485	0.011712	0.002138	4.30E-08	A	G	0.430905
Alcohol intake frequency	rs34631026	462346	-0.01691	0.003048	2.90E-08	T	C	0.446061
Usual walking pace	rs34811474	462346	-0.02018	0.003593	1.90E-08	A	G	0.230728
Alcohol intake frequency	rs35105141	462346	0.026345	0.003088	1.40E-17	T	C	0.401541
Alcohol intake frequency	rs362307	462346	0.043305	0.005802	8.40E-14	T	C	0.074582
Alcohol intake frequency	rs4002471	460443	-0.01924	0.001924	1.50E-23	T	C	0.547362
Years of educational attainment in males	rs4149513	421764	0.01173	0.001671	2.20E-12	A	G	0.493537
Ever smoked	rs4235642	462630	0.012294	0.001816	1.30E-11	G	A	0.379691
Time spent watching television	rs4240672	461981	0.01712	0.002094	3.00E-16	A	G	0.494014
Alcohol intake frequency	rs4241258	462346	0.025064	0.004403	1.30E-08	T	C	0.13763
Alcohol intake frequency	rs4242715	462346	-0.01865	0.003248	9.30E-09	A	G	0.680585
Alcohol intake frequency	rs4291983	435435	-0.0084	0.001425	3.70E-09	A	C	0.517576
Years of educational attainment in females	rs4302893	446462	0.007389	0.0013	1.30E-08	A	G	0.334189
Coffee consumption	rs4410790	457873	-0.01096	0.001229	4.80E-19	C	T	0.630852
Coffee consumption	rs4410790	447485	0.040551	0.002195	3.40E-76	C	T	0.631224
Coffee consumption	rs4410790	428860	0.039072	0.001673	1.20E-120	C	T	0.632141
Coffee consumption	rs4410790	427588	-0.03087	0.001877	8.60E-61	C	T	0.629877
Coffee consumption	rs4410790	441640	-0.01091	0.001847	3.40E-09	C	T	0.630668
Alcohol intake frequency	rs4417025	462346	-0.01884	0.003165	2.70E-09	A	G	0.361153
Time spent watching television	rs4503172	451486	0.01296	0.002293	1.60E-08	T	C	0.608301
Alcohol intake frequency	rs4503294	462346	0.018148	0.00307	3.40E-09	T	C	0.565333
Alcohol intake frequency	rs4510068	460443	-0.01301	0.001971	4.00E-11	T	G	0.402798
Years of educational attainment in females	rs45501495	460443	0.01568	0.002257	3.70E-12	T	C	0.236013
Alcohol intake frequency	rs461599	462346	-0.01919	0.00304	2.70E-10	C	A	0.462259
Alcohol intake frequency	rs4665972	452236	-0.01424	0.002043	3.20E-12	C	T	0.604541
Alcohol intake frequency	rs4726481	462346	0.021761	0.003102	2.30E-12	T	G	0.400576

Alcohol intake frequency	rs473098	462346	-0.02174	0.003043	9.10E-13	T	C	0.557689
Alcohol intake frequency	rs4739105	462630	0.015465	0.00218	1.30E-12	C	T	0.787781
Alcohol intake frequency	rs476828	428860	0.017346	0.001895	5.60E-20	C	T	0.237409
Years of educational attainment in females	rs4799949	462630	-0.01098	0.001872	4.50E-09	T	C	0.667226
Years of educational attainment	rs4800488	421764	0.011984	0.001672	7.70E-13	A	C	0.489858
Ever smoked	rs4851029	448651	0.010174	0.001564	7.80E-11	G	T	0.526969
Years of educational attainment	rs4860797	462630	0.011667	0.001808	1.10E-10	A	G	0.601276
Alcohol intake frequency	rs489062	462346	0.01665	0.003053	4.90E-08	A	G	0.437454
Alcohol intake frequency	rs4916723	462346	0.023948	0.0031	1.10E-14	C	A	0.420617
Alcohol intake frequency	rs4940926	462346	-0.0191	0.003441	2.80E-08	C	T	0.735045
Alcohol intake frequency	rs4953150	446462	-0.00844	0.001293	6.60E-11	T	C	0.344088
Alcohol intake frequency	rs4968391	462346	-0.01927	0.003227	2.30E-09	T	G	0.674892
Ever smoked	rs4984685	452236	0.013585	0.002481	4.40E-08	A	G	0.201006
Alcohol intake frequency	rs504675	451486	0.027442	0.002342	1.00E-31	T	C	0.352634
Alcohol intake frequency	rs528301	462630	0.015261	0.00177	6.60E-18	A	G	0.554846
Time spent watching television	rs531358	451486	0.013167	0.002337	1.80E-08	T	C	0.649768
Alcohol intake frequency	rs550942	462346	0.022401	0.003989	2.00E-08	T	C	0.823865
Usual walking pace	rs56094641	460880	0.012588	0.001651	2.50E-14	G	A	0.404619
Alcohol intake frequency	rs58905411	462346	-0.02663	0.003078	5.10E-18	A	G	0.410052
Time spent watching television	rs6010651	461981	-0.01241	0.002168	1.10E-08	C	A	0.379439
Alcohol intake frequency	rs6030200	462346	-0.01953	0.003271	2.40E-09	A	G	0.31415
Alcohol intake frequency	rs61873510	462346	0.020374	0.003303	6.90E-10	T	G	0.32785
Years of educational attainment	rs62034322	451486	-0.01394	0.0023	1.40E-09	A	G	0.379848
Years of educational attainment	rs62097995	462346	0.020002	0.003067	6.90E-11	A	T	0.423591
Alcohol intake frequency	rs62305780	462346	-0.04852	0.005066	9.90E-22	G	C	0.102253
Alcohol intake frequency	rs62339673	462346	0.018294	0.003154	6.60E-09	A	C	0.626705
Years of educational attainment in females	rs62396185	461053	-0.01484	0.001951	2.80E-14	C	G	0.260057
Years of educational attainment	rs62442924	441640	0.012731	0.002257	1.70E-08	T	C	0.194276
Alcohol intake frequency	rs62466318	462346	-0.02549	0.003774	1.40E-11	T	C	0.202827
Usual walking pace	rs6482190	435435	0.011272	0.00159	1.40E-12	G	A	0.719408
Alcohol intake frequency	rs650558	462346	0.020736	0.003508	3.40E-09	T	C	0.247918
Years of educational attainment	rs6727281	462346	-0.02432	0.00392	5.50E-10	T	C	0.184023
Alcohol intake frequency	rs6780346	462630	-0.01684	0.001817	1.90E-20	T	C	0.620997
Ever smoked	rs6887291	462630	0.012676	0.001836	5.10E-12	G	T	0.636126
Alcohol intake frequency	rs6957745	427588	-0.01529	0.002262	1.40E-11	C	T	0.202686
Alcohol intake frequency	rs6957745	460880	-0.01218	0.002023	1.80E-09	C	T	0.202978
Alcohol intake frequency	rs713598	447485	0.013397	0.002157	5.20E-10	G	C	0.402254
Alcohol intake frequency	rs71386942	451486	0.014455	0.002522	9.90E-09	A	C	0.268947
Alcohol intake frequency	rs71651683	462346	-0.07046	0.012791	3.60E-08	T	C	0.0142
Years of educational attainment in females	rs7243428	460443	-0.01298	0.002292	1.50E-08	G	A	0.224648
Alcohol intake frequency	rs72787062	462346	-0.02819	0.004103	6.40E-12	A	G	0.162767
Alcohol intake frequency	rs728538	462346	0.022875	0.004063	1.80E-08	G	T	0.168868
Years of educational attainment in females	rs7302200	462346	-0.01842	0.003198	8.40E-09	A	G	0.339998
Alcohol intake frequency	rs73050128	462346	-0.026	0.004091	2.10E-10	A	C	0.164488
Time spent watching television	rs73096946	451486	-0.02059	0.003067	1.90E-11	C	T	0.157396
Alcohol intake frequency	rs7330939	462346	-0.02133	0.003405	3.70E-10	T	C	0.720352
Time spent watching television	rs73802707	452236	-0.01593	0.002762	8.00E-09	T	C	0.153694
Time spent watching television	rs7386207	451486	-0.0125	0.002269	3.60E-08	T	C	0.563517
Alcohol intake frequency	rs739320	446462	-0.009	0.001277	1.90E-12	C	T	0.60575
Alcohol intake frequency	rs7514579	462346	0.019667	0.003598	4.60E-08	C	A	0.232457
Years of educational attainment	rs7531118	461981	-0.01407	0.002113	2.80E-11	C	T	0.531133
Years of educational attainment	rs75641275	421764	-0.01416	0.002385	2.90E-09	C	A	0.143372
Years of educational attainment in females	rs7599488	421764	-0.01042	0.001687	6.70E-10	T	C	0.426408
Years of educational attainment in females	rs76082653	462346	0.046427	0.006687	3.80E-12	T	C	0.054327
Years of educational attainment	rs7610856	462346	-0.02386	0.00307	7.70E-15	A	C	0.429053
Alcohol intake frequency	rs780093	428860	0.013294	0.001657	1.00E-15	C	T	0.615839
Alcohol intake frequency	rs780094	462346	-0.05099	0.003105	1.30E-60	C	T	0.615206
Alcohol intake frequency	rs780569	462346	0.019803	0.003365	4.00E-09	A	T	0.70882
Ever smoked	rs782221	427588	-0.01271	0.002262	1.90E-08	T	C	0.200429
Time spent watching television	rs7829800	461900	0.011458	0.001943	3.70E-09	G	A	0.670759
Time spent watching television	rs7829800	421764	-0.01045	0.001787	5.10E-09	G	A	0.671041
Years of educational attainment in females	rs7869969	446462	0.007567	0.001299	5.70E-09	G	A	0.330836
Alcohol intake frequency	rs790561	435435	0.012474	0.001562	1.40E-15	G	A	0.704134
Alcohol intake frequency	rs790564	460443	0.014691	0.002148	7.90E-12	C	A	0.722952
Years of educational attainment in males	rs7916868	421764	0.009607	0.001672	9.10E-09	T	A	0.503499
Alcohol intake frequency	rs80292319	462346	-0.03937	0.006496	1.40E-09	C	T	0.057704
Alcohol intake frequency	rs8043563	462346	0.023365	0.003471	1.70E-11	C	G	0.737192
Ever smoked	rs817223	446462	-0.00727	0.001223	2.80E-09	C	T	0.481314
Alcohol intake frequency	rs838133	461981	0.019014	0.002166	1.60E-18	G	A	0.549369
Alcohol intake frequency	rs838133	441640	-0.02065	0.001843	3.90E-29	G	A	0.549201
Alcohol intake frequency	rs838133	448651	0.011688	0.001615	4.50E-13	G	A	0.549575
Alcohol intake frequency	rs838133	460880	0.016184	0.001676	4.70E-22	G	A	0.549409
Alcohol intake frequency	rs838133	460162	0.010894	0.001525	9.00E-13	G	A	0.549354
Alcohol intake frequency	rs838145	462346	0.021955	0.003055	6.70E-13	A	G	0.542982
Alcohol intake frequency	rs8614	462346	0.024781	0.003925	2.70E-10	A	C	0.182509
Alcohol intake frequency	rs9349379	462346	-0.01935	0.003082	3.50E-10	G	A	0.405493

Years of educational attainment in females	rs9372625	462346	-0.02556	0.003125	2.90E-16	A	G	0.381706
Years of educational attainment in females	rs9372734	457873	-0.00802	0.001188	1.40E-11	T	C	0.482495
Years of educational attainment in females	rs9374896	441640	0.017527	0.001792	1.30E-22	T	C	0.466274
Usual walking pace	rs9379832	460162	-0.01147	0.001706	1.80E-11	G	A	0.255452
Years of educational attainment in females	rs9385269	421764	0.012067	0.001682	7.20E-13	T	C	0.524565
Alcohol intake frequency	rs9403297	462346	0.018823	0.00313	1.80E-09	A	G	0.372967
Time spent watching television	rs9517948	446462	0.006953	0.001233	1.70E-08	T	C	0.451352
Usual walking pace	rs9648478	462346	0.01686	0.003029	2.60E-08	A	G	0.510245
Years of educational attainment	rs975303	451486	0.021276	0.002907	2.50E-13	G	A	0.181313
Alcohol intake frequency	rs9814516	462346	-0.02511	0.003556	1.60E-12	T	G	0.237423
Alcohol intake frequency	rs9829192	462346	0.016932	0.00305	2.80E-08	T	G	0.435133
Ever smoked	rs9835772	462630	0.011451	0.002052	2.40E-08	T	A	0.243664
Ever smoked	rs9835772	457873	-0.00808	0.001381	4.90E-09	T	A	0.243662
Ever smoked	rs9843358	462630	0.018953	0.002344	6.20E-16	T	C	0.171903
Alcohol intake frequency	rs9906502	462346	0.023788	0.003962	1.90E-09	A	G	0.176998
Alcohol intake frequency	rs9912298	462346	0.020589	0.00359	9.70E-09	C	A	0.239585
Years of educational attainment in males	rs9919429	446462	-0.00672	0.001223	3.80E-08	G	A	0.486008
Alcohol intake frequency	rs9923768	461900	0.010512	0.001859	1.60E-08	A	G	0.59853
Usual walking pace	rs9937354	447485	-0.01409	0.002143	4.90E-11	A	G	0.424074
Years of educational attainment in females	rs9997448	461900	-0.01046	0.001883	2.70E-08	T	C	0.369236

Table S4:Palindromic SNPs with intermediate allele frequencies

exposure	SNP	samplesize.exposure	beta.exposure	se.exposure	pval.exposure	effect_allele.exposure	other_allele.exposure	eaf.exposure
Beef intake || id:ukb-b-2862	rs9407624	461053	-0.01376	0.001716	1.10E-15	A	T	0.488187
Bread intake || id:ukb-b-11348	rs7276867	452236	0.011348	0.002003	1.50E-08	C	G	0.541677
Bread intake || id:ukb-b-11348	rs9832088	452236	0.014729	0.001989	1.30E-13	A	T	0.521617
Bread intake || id:ukb-b-11348	rs9881332	452236	0.01137	0.002022	1.90E-08	G	C	0.581804
Cereal intake || id:ukb-b-15926	rs10837531	441640	0.010784	0.001797	2.00E-09	G	C	0.455002
Cereal intake || id:ukb-b-15926	rs3859193	441640	-0.01033	0.001799	9.50E-09	A	T	0.470074
Cereal intake || id:ukb-b-15926	rs627185	441640	-0.01083	0.001791	1.50E-09	G	C	0.544427
Cheese intake || id:ukb-b-1489	rs1024853	451486	-0.01286	0.002262	1.30E-08	G	C	0.437902
Coffee intake || id:ukb-b-5237	rs10119174	428860	-0.0094	0.001642	1.00E-08	C	G	0.571035
Dried fruit intake || id:ukb-b-16576	rs11037497	421764	0.01044	0.001684	5.70E-10	C	G	0.446175
Fresh fruit intake || id:ukb-b-3881	rs2302593	446462	0.008528	0.001223	3.10E-12	G	C	0.486625
Lamb/mutton intake || id:ukb-b-14179	rs2140714	460006	-0.00852	0.001476	7.70E-09	G	C	0.557738
Oily fish intake || id:ukb-b-2209	rs11986122	460443	0.014817	0.001949	2.90E-14	G	C	0.422568
Oily fish intake || id:ukb-b-2209	rs9886779	460443	-0.01072	0.001929	2.70E-08	A	T	0.439213
Poultry intake || id:ukb-b-8006	rs7046351	461900	0.011075	0.001817	1.10E-09	A	T	0.509739
Salad / raw vegetable intake || id:ukb-b-1996	rs13102393	435435	0.007979	0.00143	2.40E-08	G	C	0.499077
Salad / raw vegetable intake || id:ukb-b-1996	rs9427220	435435	-0.00801	0.001442	2.80E-08	T	A	0.554661
Salt added to food || id:ukb-b-8121	rs13084934	462630	-0.00968	0.001767	4.40E-08	T	A	0.494691
Salt added to food || id:ukb-b-8121	rs9375448	462630	-0.01168	0.001762	3.40E-11	T	A	0.492981
Salt added to food || id:ukb-b-8121	rs976179	462630	0.010067	0.001765	1.20E-08	T	A	0.485324
Hot drink temperature || id:ukb-b-14203	rs12038134	457873	0.006537	0.001187	3.60E-08	A	T	0.508213
Hot drink temperature || id:ukb-b-14203	rs58726064	457873	0.006771	0.001196	1.50E-08	G	C	0.442086
Tea intake || id:ukb-b-6066	rs2783129	447485	-0.01173	0.002133	3.80E-08	G	C	0.484878
Alcohol intake frequency. || id:ukb-b-5779	rs9958320	462346	0.024855	0.004271	5.90E-09	C	T	0.153147

Table S5: Numbers of SNP in final anaylsis

Dietary habits	Number of SNPs
Alcohol intake frequency. || id:ukb-b-5779	14
Beef intake || id:ukb-b-2862	13
Bread intake || id:ukb-b-11348	26
Cereal intake || id:ukb-b-15926	30
Cheese intake || id:ukb-b-1489	46
Coffee intake || id:ukb-b-5237	29
Cooked vegetable intake || id:ukb-b-8089	11
Dried fruit intake || id:ukb-b-16576	29
Fresh fruit intake || id:ukb-b-3881	39
Hot drink temperature || id:ukb-b-14203	57
Lamb/mutton intake || id:ukb-b-14179	29
Non-oily fish intake || id:ukb-b-17627	6
Oily fish intake || id:ukb-b-2209	50
Pork intake || id:ukb-b-5640	11
Poultry intake || id:ukb-b-8006	3
Processed meat intake || id:ukb-b-6324	18
Salad / raw vegetable intake || id:ukb-b-1996	14
Salt added to food || id:ukb-b-8121	87
Tea intake || id:ukb-b-6066	32
Water intake || id:ukb-b-14898	34

Table S6: MR results of IVW method for association of dietary habits and chronic hip pain.

Exposure	Number of SNPs	β	SE_{β}	*p* -value	adjusted *p* value
Beef intake	13	-0.01	0.07	0.824	0.891
Bread intake	27	0.03	0.04	0.483	0.691
Cereal intake	30	-0.05	0.04	0.244	0.52
Cheese intake	46	-0.08	0.03	**0.005**	0.109
Coffee intake	29	0.07	0.05	0.145	0.468
Cooked vegetable intake	11	0.13	0.08	0.133	0.468
Dried fruit intake	29	-0.07	0.05	0.114	0.468
Fresh fruit intake	39	0.07	0.05	0.174	0.468
Lamb/mutton intake	29	-0.11	0.05	**0.033**	0.217
Non-oily fish intake	6	-0.06	0.1	0.558	0.744
Oily fish intake	50	-0.07	0.03	**0.027**	0.217
Pork intake	11	-0.02	0.09	0.847	0.891
Poultry intake	3	0	0.15	0.985	0.985
Processed meat intake	18	0.06	0.05	0.26	0.52
Salad / raw vegetable intake	14	-0.08	0.11	0.443	0.691
Salt added to food	87	0.03	0.02	0.187	0.468
Water intake	34	-0.03	0.04	0.478	0.691
Hot drink temperature	57	-0.04	0.04	0.406	0.691
Tea intake	32	0.02	0.03	0.649	0.811
Alcohol intake frequency	15	-0.01	0.04	0.796	0.891

Table S7: MR results of IVW method for association of dietary habits and chronic headache.

Exposure	Number of SNPs	β	SE_{β}	*p* -value	adjusted *p* value
Beef intake	13	0.02	0.09	0.814	0.904
Bread intake	27	0.03	0.03	0.428	0.892
Cereal intake	30	0	0.04	0.907	0.907
Cheese intake	46	0.04	0.03	0.158	0.892
Coffee intake	29	-0.05	0.05	0.388	0.892
Cooked vegetable intake	11	0.01	0.07	0.846	0.904
Dried fruit intake	29	-0.05	0.04	0.249	0.892
Fresh fruit intake	39	-0.02	0.06	0.711	0.904
Lamb/mutton intake	29	0.02	0.05	0.612	0.904
Non-oily fish intake	6	-0.11	0.14	0.446	0.892
Oily fish intake	50	-0.03	0.03	0.322	0.892
Pork intake	11	0.03	0.14	0.853	0.904
Poultry intake	3	-0.03	0.13	0.794	0.904
Processed meat intake	18	-0.02	0.04	0.63	0.904
Salad / raw vegetable intake	14	0.06	0.07	0.373	0.892
Salt added to food	87	0	0.03	0.859	0.904
Water intake	34	-0.06	0.05	0.243	0.892
Hot drink temperature	57	0.08	0.05	0.091	0.892
Tea intake	32	-0.08	0.03	**0.008**	0.155
Alcohol intake frequency	15	0.03	0.04	0.506	0.904

Table S8: MR results of IVW method for association of dietary habits and chronic knee pain.

Exposure	Number of SNPs	β	$SE_β$	*p* -value	adjusted *p* value
Beef intake	13	-0.03	0.05	0.602	0.935
Bread intake	27	-0.01	0.04	0.74	0.935
Cereal intake	30	0	0.03	0.882	0.98
Cheese intake	46	-0.02	0.02	0.326	0.844
Coffee intake	29	0.01	0.03	0.748	0.935
Cooked vegetable intake	11	0.08	0.06	0.199	0.665
Dried fruit intake	29	-0.03	0.03	0.409	0.844
Fresh fruit intake	39	0	0.04	0.999	0.999
Lamb/mutton intake	29	-0.1	0.04	**0.009**	0.171
Non-oily fish intake	6	0.06	0.07	0.458	0.844
Oily fish intake	50	-0.04	0.03	0.134	0.619
Pork intake	11	0.02	0.06	0.746	0.935
Poultry intake	3	0.18	0.11	0.084	0.585
Processed meat intake	18	0.02	0.04	0.633	0.935
Salad / raw vegetable intake	14	0	0.08	0.983	0.999
Salt added to food	87	0.01	0.02	0.464	0.844
Water intake	34	-0.05	0.03	0.088	0.585
Hot drink temperature	57	0.01	0.03	0.859	0.98
Tea intake	32	-0.02	0.02	0.376	0.844
Alcohol intake frequency	15	0.04	0.03	0.155	0.619

Table S9: MR results of WM method for association of dietary habits and multisite chronic pain.

Exposure	Number of SNPs	β	SE_{β}	*p* -value	adjusted *p* value
Beef intake	13	0.04	0.10	0.684	0.912
Bread intake	26	-0.06	0.06	0.298	0.481
Cereal intake	30	-0.24	0.06	**<0.001**	**0.002**
Cheese intake	46	-0.08	0.04	0.052	0.138
Coffee intake	29	0.08	0.07	0.255	0.481
Cooked vegetable intake	11	-0.01	0.11	0.922	0.97
Dried fruit intake	29	-0.07	0.07	0.313	0.481
Fresh fruit intake	39	-0.17	0.08	**0.026**	0.104
Lamb/mutton intake	29	0.02	0.08	0.838	0.956
Non-oily fish intake	6	-0.25	0.14	0.072	0.159
Oily fish intake	50	0.05	0.05	0.302	0.481
Pork intake	11	0.36	0.13	**0.006**	**0.028**
Poultry intake	3	0.70	0.19	**<0.001**	**0.002**
Processed meat intake	18	0.07	0.07	0.35	0.499
Salad / raw vegetable intake	14	-0.25	0.12	0.038	0.127
Salt added to food	87	0.07	0.04	0.055	0.138
Water intake	33	0.00	0.06	0.985	0.985
Hot drink temperature	57	-0.02	0.06	0.775	0.956
Tea intake	31	-0.01	0.06	0.86	0.956
Alcohol intake frequency	14	0.22	0.06	**<0.001**	**0.002**

Table S10: MR results of WM method for association of dietary habits and chronic back pain.

Exposure	Number of SNPs	β	$SE_{β}$	*p* -value	adjusted *p* value
Beef intake	13	0	0.08	0.98	0.98
Bread intake	27	0.02	0.04	0.65	0.867
Cereal intake	30	-0.05	0.04	0.228	0.757
Cheese intake	46	-0.03	0.03	0.265	0.757
Coffee intake	29	-0.03	0.05	0.606	0.867
Cooked vegetable intake	11	0.04	0.08	0.643	0.867
Dried fruit intake	29	-0.06	0.05	0.196	0.757
Fresh fruit intake	39	-0.05	0.06	0.425	0.849
Lamb/mutton intake	29	-0.01	0.06	0.927	0.98
Non-oily fish intake	6	0.09	0.1	0.361	0.803
Oily fish intake	50	0	0.03	0.96	0.98
Pork intake	11	0.32	0.09	**0.001**	**0.011**
Poultry intake	3	0.23	0.14	0.096	0.482
Processed meat intake	18	-0.05	0.05	0.338	0.803
Salad / raw vegetable intake	14	0.06	0.09	0.512	0.867
Salt added to food	87	0.01	0.03	0.753	0.935
Water intake	34	-0.02	0.04	0.584	0.867
Hot drink temperature	57	-0.01	0.05	0.795	0.935
Tea intake	32	-0.06	0.04	0.089	0.482
Alcohol intake frequency	15	0.09	0.04	**0.016**	0.158

Table S11: MR results of WM method for association of dietary habits and chronic hip pain.

Exposure	Number of SNPs	β	SE_{β}	*p* -value	adjusted *p* value
Beef intake	13	-0.01	0.09	0.926	0.952
Bread intake	27	0.06	0.05	0.296	0.658
Cereal intake	30	-0.07	0.06	0.261	0.658
Cheese intake	46	-0.08	0.04	0.053	0.526
Coffee intake	29	0.07	0.06	0.283	0.658
Cooked vegetable intake	11	0.1	0.11	0.372	0.697
Dried fruit intake	29	-0.08	0.06	0.217	0.658
Fresh fruit intake	39	0.09	0.08	0.258	0.658
Lamb/mutton intake	29	-0.12	0.07	0.084	0.561
Non-oily fish intake	6	-0.1	0.13	0.425	0.708
Oily fish intake	50	-0.08	0.04	**0.048**	0.526
Pork intake	11	0.05	0.12	0.682	0.802
Poultry intake	3	0.11	0.2	0.594	0.78
Processed meat intake	18	0.1	0.07	0.143	0.658
Salad / raw vegetable intake	14	-0.02	0.12	0.84	0.934
Salt added to food	87	0.02	0.03	0.614	0.78
Water intake	34	-0.03	0.06	0.624	0.78
Hot drink temperature	57	-0.05	0.06	0.383	0.697
Tea intake	32	0.03	0.05	0.493	0.758
Alcohol intake frequency	15	0	0.05	0.952	0.952

Table S12: MR results of WM method for association of dietary habits and chronic neck/shoulder pain.

Exposure	Number of SNPs	β	SE_{β}	*p* -value	adjusted *p* value
Beef intake	13	-0.03	0.07	0.71	0.902
Bread intake	27	-0.05	0.04	0.298	0.709
Cereal intake	30	-0.04	0.04	0.4	0.8
Cheese intake	46	-0.01	0.03	0.784	0.902
Coffee intake	29	0.01	0.05	0.8	0.902
Cooked vegetable intake	11	0.03	0.09	0.707	0.902
Dried fruit intake	29	-0.02	0.05	0.772	0.902
Fresh fruit intake	39	-0.08	0.06	0.167	0.477
Lamb/mutton intake	29	-0.11	0.06	0.053	0.32
Non-oily fish intake	6	-0.11	0.11	0.319	0.709
Oily fish intake	50	-0.06	0.03	0.059	0.32
Pork intake	11	0.18	0.1	0.064	0.32
Poultry intake	3	0.32	0.15	**0.03**	0.32
Processed meat intake	18	-0.09	0.06	0.15	0.477
Salad / raw vegetable intake	14	0	0.09	0.957	0.957
Salt added to food	87	-0.01	0.03	0.702	0.902
Water intake	34	0.01	0.04	0.857	0.902
Hot drink temperature	57	0.02	0.05	0.676	0.902
Tea intake	32	0.01	0.04	0.849	0.902
Alcohol intake frequency	15	0.07	0.04	0.097	0.389

Table S13: MR results of WM method for association of dietary habits and chronic headache.

Exposure	Number of SNPs	β	SE_{β}	*p* -value	adjusted *p* value
Beef intake	13	0.06	0.09	0.504	0.836
Bread intake	27	0.03	0.05	0.557	0.836
Cereal intake	30	0.04	0.05	0.417	0.836
Cheese intake	46	0	0.04	0.975	0.99
Coffee intake	29	-0.01	0.07	0.877	0.974
Cooked vegetable intake	11	-0.06	0.11	0.546	0.836
Dried fruit intake	29	-0.03	0.06	0.581	0.836
Fresh fruit intake	39	-0.04	0.07	0.547	0.836
Lamb/mutton intake	29	0.04	0.07	0.511	0.836
Non-oily fish intake	6	-0.15	0.13	0.247	0.836
Oily fish intake	50	0.02	0.04	0.618	0.836
Pork intake	11	0.14	0.13	0.259	0.836
Poultry intake	3	-0.04	0.18	0.832	0.974
Processed meat intake	18	-0.03	0.07	0.65	0.836
Salad / raw vegetable intake	14	0	0.09	0.99	0.99
Salt added to food	87	0.01	0.03	0.641	0.836
Water intake	34	-0.03	0.05	0.564	0.836
Hot drink temperature	57	0.07	0.06	0.245	0.836
Tea intake	32	-0.04	0.05	0.357	0.836
Alcohol intake frequency	15	-0.02	0.05	0.669	0.836

Table S14: MR results of WM method for association of dietary habits and chronic knee pain.

Exposure	Number of SNPs	β	SE_{β}	*p* -value	adjusted *p* value
Beef intake	13	-0.12	0.07	0.081	0.751
Bread intake	27	-0.03	0.04	0.473	0.751
Cereal intake	30	-0.04	0.04	0.323	0.751
Cheese intake	46	-0.03	0.03	0.329	0.751
Coffee intake	29	-0.04	0.05	0.393	0.751
Cooked vegetable intake	11	0.05	0.08	0.563	0.751
Dried fruit intake	29	-0.05	0.05	0.255	0.751
Fresh fruit intake	39	0	0.06	0.994	0.994
Lamb/mutton intake	29	-0.11	0.05	0.054	0.751
Non-oily fish intake	6	0.05	0.09	0.602	0.752
Oily fish intake	50	0.01	0.03	0.821	0.966
Pork intake	11	-0.07	0.08	0.383	0.751
Poultry intake	3	0.01	0.15	0.943	0.994
Processed meat intake	18	-0.03	0.05	0.508	0.751
Salad / raw vegetable intake	14	-0.07	0.08	0.386	0.751
Salt added to food	87	0	0.03	0.951	0.994
Water intake	34	-0.06	0.04	0.166	0.751
Hot drink temperature	57	0.05	0.05	0.273	0.751
Tea intake	32	-0.02	0.04	0.532	0.751
Alcohol intake frequency	15	0.03	0.04	0.518	0.751

Table S15: MR results of PRESSO method for association of dietary habits and multi-site chronic pain.

Exposure	Number of SNPs	β	SE_{β}	*p* -value	adjusted *p* value
Beef intake	13	0.13	0.1	0.253	0.562
Bread intake	26	-0.03	0.06	0.647	0.827
Cereal intake	30	-0.16	0.06	**0.013**	**0.049**
Cheese intake	46	-0.12	0.04	**0.003**	**0.017**
Coffee intake	29	-0.04	0.06	0.523	0.827
Cooked vegetable intake	11	0.11	0.11	0.318	0.626
Dried fruit intake	29	-0.22	0.06	**0.002**	**0.017**
Fresh fruit intake	39	-0.15	0.07	**0.032**	0.093
Lamb/mutton intake	29	-0.03	0.08	0.719	0.846
Non-oily fish intake	6	-0.02	0.22	0.928	0.937
Oily fish intake	50	-0.05	0.05	0.344	0.626
Pork intake	11	0.36	0.12	**0.015**	**0.049**
Poultry intake	3	0.63	0.1	**0.007**	**0.036**
Processed meat intake	18	0.01	0.08	0.937	0.937
Salad / raw vegetable intake	14	-0.07	0.12	0.542	0.827
Salt added to food	87	0.11	0.04	**0.002**	**0.017**
Water intake	33	-0.01	0.06	0.809	0.899
Hot drink temperature	57	0.03	0.07	0.662	0.827
Tea intake	31	-0.04	0.06	0.589	0.827
Alcohol intake frequency	14	0.17	0.08	0.053	0.133

Table S16: MR results of PRESSO method for association of dietary habits and chronic back pain.

Exposure	Number of SNPs	β	SE_{β}	*p*-value	adjusted *p* value
Beef intake	13	0.11	0.06	0.105	0.349
Bread intake	27	-0.01	0.03	0.782	0.9
Cereal intake	30	-0.08	0.03	**0.026**	0.11
Cheese intake	46	-0.06	0.02	**0.005**	0.097
Coffee intake	29	-0.02	0.03	0.62	0.886
Cooked vegetable intake	11	0.06	0.05	0.264	0.529
Dried fruit intake	29	-0.05	0.03	0.168	0.48
Fresh fruit intake	39	-0.05	0.04	0.262	0.529
Lamb/mutton intake	29	0.02	0.04	0.708	0.9
Non-oily fish intake	6	0.11	0.09	0.25	0.529
Oily fish intake	50	-0.01	0.02	0.81	0.9
Pork intake	11	0.23	0.08	**0.023**	0.11
Poultry intake	3	0.26	0.07	**0.028**	0.11
Processed meat intake	18	-0.02	0.04	0.728	0.9
Salad / raw vegetable intake	14	0	0.06	0.966	0.966
Salt added to food	87	0.01	0.02	0.582	0.886
Water intake	34	-0.02	0.03	0.609	0.886
Hot drink temperature	57	0	0.04	0.939	0.966
Tea intake	32	-0.02	0.03	0.468	0.851
Alcohol intake frequency	15	0.08	0.03	**0.013**	0.11

Table S17: MR results of PRESSO method for association of dietary habits and chronic hip pain.

Exposure	Number of SNPs	β	$SE_β$	*p* -value	adjusted *p* value
Beef intake	13	-0.01	0.05	0.919	0.919
Bread intake	27	0.02	0.04	0.594	0.742
Cereal intake	30	-0.03	0.05	0.501	0.742
Cheese intake	46	-0.08	0.03	**0.003**	0.054
Coffee intake	29	0.07	0.04	0.071	0.449
Cooked vegetable intake	11	0.13	0.08	0.144	0.449
Dried fruit intake	29	-0.07	0.05	0.157	0.449
Fresh fruit intake	39	0.07	0.06	0.255	0.638
Lamb/mutton intake	29	-0.12	0.04	**0.005**	0.054
Non-oily fish intake	6	-0.06	0.05	0.298	0.645
Oily fish intake	50	-0.05	0.03	0.108	0.449
Pork intake	11	-0.02	0.11	0.876	0.919
Poultry intake	3	0.02	0.13	0.891	0.919
Processed meat intake	18	0.06	0.05	0.322	0.645
Salad / raw vegetable intake	14	-0.06	0.1	0.558	0.742
Salt added to food	87	0.03	0.02	0.127	0.449
Water intake	34	-0.03	0.04	0.523	0.742
Hot drink temperature	57	-0.03	0.04	0.399	0.726
Tea intake	32	0.02	0.03	0.547	0.742
Alcohol intake frequency	15	0	0.03	0.907	0.919

Table S18: MR results of PRESSO method for association of dietary habits and chronic neck/shoulder pain.

Exposure	Number of SNPs	β	SE_{β}	*p* -value	adjusted *p* value
Beef intake	13	0	0.05	0.936	0.985
Bread intake	27	-0.05	0.03	0.124	0.385
Cereal intake	30	-0.05	0.03	0.088	0.351
Cheese intake	46	-0.03	0.02	0.257	0.543
Coffee intake	29	-0.03	0.04	0.408	0.543
Cooked vegetable intake	11	0.08	0.06	0.19	0.474
Dried fruit intake	29	-0.03	0.04	0.487	0.609
Fresh fruit intake	39	-0.03	0.04	0.404	0.543
Lamb/mutton intake	29	-0.14	0.04	**0.003**	0.069
Non-oily fish intake	6	0.01	0.13	0.932	0.985
Oily fish intake	50	-0.04	0.03	0.135	0.385
Pork intake	11	0.11	0.1	0.321	0.543
Poultry intake	3	0.31	0.05	**0.008**	0.085
Processed meat intake	18	-0.05	0.05	0.349	0.543
Salad / raw vegetable intake	14	-0.04	0.05	0.359	0.543
Salt added to food	87	0.03	0.02	**0.079**	0.351
Water intake	34	0.01	0.03	0.678	0.797
Hot drink temperature	57	0	0.03	0.985	0.985
Tea intake	32	0.03	0.03	0.273	0.543
Alcohol intake frequency	15	0.09	0.03	**0.019**	0.125

Table S19: MR results of PRESSO method for association of dietary habits and chronic headache.

Exposure	Number of SNPs	β	SE_β	*p* -value	adjusted *p* value
Beef intake	13	0.04	0.08	0.632	0.889
Bread intake	27	0.01	0.04	0.707	0.889
Cereal intake	30	0.01	0.04	0.728	0.889
Cheese intake	46	0.04	0.03	0.16	0.889
Coffee intake	29	-0.04	0.05	0.415	0.889
Cooked vegetable intake	11	0.01	0.1	0.889	0.889
Dried fruit intake	29	-0.06	0.05	0.266	0.889
Fresh fruit intake	39	-0.01	0.06	0.885	0.889
Lamb/mutton intake	29	0.03	0.05	0.61	0.889
Non-oily fish intake	6	-0.11	0.14	0.481	0.889
Oily fish intake	50	-0.02	0.03	0.436	0.889
Pork intake	11	0.03	0.14	0.856	0.889
Poultry intake	3	0.06	0.15	0.73	0.889
Processed meat intake	18	-0.02	0.05	0.692	0.889
Salad / raw vegetable intake	14	0.02	0.05	0.729	0.889
Salt added to food	87	0	0.02	0.875	0.889
Water intake	34	-0.06	0.05	0.251	0.889
Hot drink temperature	57	0.09	0.05	0.065	0.649
Tea intake	32	-0.08	0.03	**0.02**	0.395
Alcohol intake frequency	15	0.03	0.04	0.488	0.889

Table S20: MR results of PRESSO method for association of dietary habits and chronic knee pain.

Exposure	Number of SNPs	β	SE_{β}	*p* -value	adjusted *p* value
Beef intake	13	-0.03	0.05	0.548	0.913
Bread intake	27	0	0.03	0.928	0.998
Cereal intake	30	0	0.03	0.998	0.998
Cheese intake	46	-0.02	0.02	0.258	0.729
Coffee intake	29	0.01	0.03	0.71	0.989
Cooked vegetable intake	11	0.08	0.04	0.114	0.603
Dried fruit intake	29	-0.03	0.03	0.328	0.729
Fresh fruit intake	39	0	0.04	0.974	0.998
Lamb/mutton intake	29	-0.09	0.04	**0.034**	0.603
Non-oily fish intake	6	0.06	0.06	0.433	0.866
Oily fish intake	50	-0.03	0.03	0.222	0.729
Pork intake	11	0.02	0.06	0.742	0.989
Poultry intake	3	0.11	0.14	0.503	0.913
Processed meat intake	18	0.02	0.04	0.654	0.989
Salad / raw vegetable intake	14	0.01	0.06	0.834	0.998
Salt added to food	87	0.02	0.02	0.304	0.729
Water intake	34	-0.05	0.03	0.09	0.603
Hot drink temperature	57	0	0.03	0.995	0.998
Tea intake	32	-0.03	0.02	0.298	0.729
Alcohol intake frequency	15	0.04	0.03	0.121	0.603

Table S21: Heterogeneity and pleiotropy test for association of dietary habits and multi-site chronic pain.

Exposure	Heterogeneity test (MR-Egger)		Heterogeneity test (IVW)		PRESSO Method		Pleiotropy test (MR-Egger intercept test)		Steiger directionality test	
	Q	*p*	Q	*p*	Global *p*	Distortion Test *p*	Egger Inter	Inter *p*	Correct Causal Direction	Steiger *p*
Beef intake	37.350	<0.001	38.235	<0.001	<0.001	**0.025**	0.004	0.620	TRUE	< 0.001
Bread intake	83.276	<0.001	85.912	<0.001	<0.001	0.107	0.004	0.392	TRUE	< 0.001
Cereal intake	71.064	<0.001	73.388	<0.001	<0.001	NA	0.003	0.347	TRUE	< 0.001
Cheese intake	87.459	<0.001	87.490	<0.001	<0.001	0.098	<0.001	0.901	TRUE	< 0.001
Coffee intake	56.508	0.001	58.700	0.001	0.001	0.298	-0.003	0.315	TRUE	< 0.001
Cooked vegetable intake	18.200	0.033	18.392	0.049	0.052	NA	0.004	0.765	TRUE	< 0.001
Dried fruit intake	67.203	<0.001	67.779	<0.001	<0.001	0.724	0.002	0.634	TRUE	< 0.001
Fresh fruit intake	80.543	<0.001	83.220	<0.001	<0.001	0.749	-0.002	0.275	TRUE	< 0.001
Lamb/mutton	75.593	<0.001	77.058	<0.001	<0.001	0.628	-0.003	0.476	TRUE	< 0.001
Non-oily fish intake	15.713	0.003	25.205	<0.001	0.001	>0.999	0.015	0.195	TRUE	< 0.001
Oily fish intake	145.781	<0.001	147.134	<0.001	<0.001	**0.023**	-0.002	0.508	TRUE	< 0.001
Pork intake	21.477	0.011	22.102	0.015	0.026	0.574	-0.004	0.621	TRUE	< 0.001
Poultry intake	0.313	0.576	0.438	0.803	0.654	NA	-0.018	0.783	TRUE	< 0.001
Processed meat intake	56.528	<0.001	56.872	<0.001	<0.001	0.615	0.002	0.759	TRUE	< 0.001
Salad / raw vegetable intake	20.965	0.051	28.023	0.009	0.001	NA	-0.010	0.067	TRUE	< 0.001
Salt added to food	225.584	<0.001	228.171	<0.001	<0.001	0.952	0.002	0.326	TRUE	< 0.001
Water intake	78.512	<0.001	80.219	<0.001	<0.001	0.997	0.003	0.418	TRUE	< 0.001
Hot drink temperature	137.439	<0.001	142.200	<0.001	<0.001	0.228	-0.004	0.173	TRUE	< 0.001
Tea intake	125.355	<0.001	125.474	<0.001	<0.001	0.837	-0.001	0.869	TRUE	< 0.001
Alcohol intake frequency	43.307	<0.001	45.770	<0.001	<0.001	0.920	0.006	0.425	TRUE	< 0.001

Table S22: Heterogeneity and pleiotropy test for association of dietary habits and chronic neck/shoulder pain.

Exposure	Heterogeneity test (MR-Egger)		Heterogeneity test (IVW)		PRESSO Method		Pleiotropy test (MR-Egger intercept test)		Steiger directionality test	
	Q	*p*	Q	*p*	Global *p*	Distortion Test *p*	Egger Inter	Inter *p*	Correct Causal Direction	Steiger *p*
Beef intake	8.555	0.663	9.326	0.675	0.335	NA	0.003	0.399	TRUE	< 0.001
Bread intake	29.301	0.252	29.449	0.291	0.252	NA	0.001	0.725	TRUE	< 0.001
Cereal intake	26.374	0.552	27.484	0.546	0.516	NA	0.002	0.301	TRUE	< 0.001
Cheese intake	46.712	0.362	47.071	0.388	0.377	NA	0.001	0.564	TRUE	< 0.001
Coffee intake	23.719	0.646	24.259	0.668	0.438	NA	-0.001	0.469	TRUE	< 0.001
Cooked vegetable intake	6.755	0.663	8.020	0.627	0.646	NA	0.008	0.290	TRUE	< 0.001
Dried fruit intake	38.375	0.072	38.377	0.091	0.106	NA	0.000	0.969	TRUE	< 0.001
Fresh fruit intake	41.166	0.293	42.364	0.288	0.294	NA	0.001	0.306	TRUE	< 0.001
Lamb/mutton	31.770	0.241	35.997	0.143	0.165	NA	-0.004	0.069	TRUE	< 0.001
Non-oily fish	9.446	0.051	12.467	0.029	0.049	0.363	0.007	0.321	TRUE	< 0.001
Oily fish intake	59.488	0.124	59.514	0.144	0.006	0.657	0.000	0.886	TRUE	< 0.001
Pork intake	20.543	0.015	22.902	0.011	0.013	0.518	-0.007	0.336	TRUE	< 0.001
Poultry intake	0.119	0.730	0.809	0.667	0.874	NA	0.034	0.559	TRUE	< 0.001
Processed meat intake	31.326	0.012	32.610	0.013	0.012	0.262	0.003	0.430	TRUE	< 0.001
Salad / raw vegetable intake	10.104	0.607	10.233	0.675	0.812	NA	-0.001	0.726	TRUE	< 0.001
Salt added to food	101.492	0.107	108.403	0.052	0.055	NA	0.002	0.018	TRUE	< 0.001
Water intake	23.421	0.865	24.711	0.850	0.850	NA	-0.002	0.264	TRUE	< 0.001
Hot drink temperature	52.064	0.588	52.064	0.625	0.594	NA	0.000	0.988	TRUE	< 0.001
Tea intake	32.074	0.364	32.865	0.376	0.437	NA	0.001	0.396	TRUE	< 0.001
Alcohol intake frequency	17.477	0.178	18.133	0.201	0.157	NA	0.003	0.497	TRUE	< 0.001

Table S23: Heterogeneity and pleiotropy test for association of dietary habits and chronic hip pain.

Exposure	Heterogeneity test (MR-Egger)		Heterogeneity test (IVW)		PRESSO Method		Pleiotropy test (MR-Egger intercept test)		Steiger directionality test	
	Q	*p*	Q	*p*	Global *p*	Distortion Test *p*	Egger Inter	Inter *p*	Correct Causal Direction	Steiger *p*
Beef intake	6.850	0.811	7.674	0.810	0.858	NA	-0.004	0.384	TRUE	< 0.001
Bread intake	25.804	0.418	25.819	0.473	0.534	NA	0.000	0.904	TRUE	< 0.001
Cereal intake	38.630	0.087	39.007	0.102	0.063	NA	0.001	0.605	TRUE	< 0.001
Cheese intake	37.820	0.733	37.823	0.767	0.803	NA	0.000	0.952	TRUE	< 0.001
Coffee intake	18.575	0.885	18.720	0.907	0.929	NA	-0.001	0.706	TRUE	< 0.001
Cooked vegetable intake	7.068	0.630	8.976	0.534	0.546	NA	0.012	0.200	TRUE	< 0.001
Dried fruit intake	28.314	0.395	29.170	0.404	0.432	NA	-0.002	0.374	TRUE	< 0.001
Fresh fruit intake	52.361	0.048	52.601	0.058	0.075	NA	-0.001	0.683	TRUE	< 0.001
Lamb/mutton intake	15.800	0.957	16.604	0.956	0.937	NA	-0.002	0.378	TRUE	< 0.001
Non-oily fish intake	1.169	0.883	1.275	0.938	0.943	NA	0.002	0.762	TRUE	< 0.001
Oily fish intake	53.893	0.259	56.903	0.205	0.178	NA	0.003	0.108	TRUE	< 0.001
Pork intake	14.634	0.101	14.639	0.146	0.168	NA	0.000	0.959	TRUE	< 0.001
Poultry intake	0.904	0.342	3.106	0.212	0.434	NA	-0.078	0.378	TRUE	< 0.001
Processed meat intake	20.046	0.218	20.762	0.237	0.247	NA	0.003	0.461	TRUE	< 0.001
Salad / raw vegetable intake	22.952	0.028	23.577	0.035	0.078	NA	0.003	0.578	TRUE	< 0.001
Salt added to	79.477	0.649	79.485	0.677	0.670	NA	0.000	0.929	TRUE	< 0.001
Water intake	39.420	0.172	39.803	0.193	0.201	NA	0.001	0.581	TRUE	< 0.001
Hot drink temperature	50.278	0.655	50.726	0.674	0.790	NA	-0.001	0.506	TRUE	< 0.001
Tea intake	26.223	0.664	30.033	0.516	0.535	NA	-0.004	0.060	TRUE	< 0.001
Alcohol intake frequency	9.825	0.708	10.255	0.743	0.649	NA	0.003	0.524	TRUE	< 0.001

Table S24: Heterogeneity and pleiotropy test for association of dietary habits and chronic back pain.

Exposure	Heterogeneity test (MR-Egger)		Heterogeneity test (IVW)		PRESSO Method		Pleiotropy test (MR-Egger intercept test)		Steiger directionality test	
	Q	*p*	Q	*p*	Global *p*	Distortion Test *p*	Egger Inter	Inter *p*	Correct Causal Direction	Steiger *p*
Beef intake	19.471	0.053	20.990	0.051	0.044	NA	-0.004	0.374	TRUE	< 0.001
Bread intake	35.295	0.083	36.388	0.085	0.091	NA	-0.002	0.387	TRUE	< 0.001
Cereal intake	42.834	0.036	43.447	0.041	0.052	NA	0.001	0.532	TRUE	< 0.001
Cheese intake	41.687	0.571	41.708	0.612	0.633	NA	0.000	0.887	TRUE	< 0.001
Coffee intake	28.494	0.386	29.040	0.410	0.467	NA	0.001	0.478	TRUE	< 0.001
Cooked vegetable intake	6.079	0.732	6.525	0.769	0.781	NA	0.004	0.521	TRUE	< 0.001
Dried fruit intake	28.464	0.387	28.945	0.415	0.452	NA	0.001	0.505	TRUE	< 0.001
Fresh fruit intake	45.648	0.156	46.464	0.163	0.178	NA	0.001	0.421	TRUE	< 0.001
Lamb/mutton	31.822	0.239	38.944	0.082	0.081	NA	-0.005	0.021	TRUE	< 0.001
Non-oily fish	5.118	0.275	6.371	0.272	0.309	NA	0.004	0.378	TRUE	< 0.001
Oily fish intake	57.957	0.154	58.582	0.164	0.054	NA	-0.001	0.475	TRUE	< 0.001
Pork intake	14.711	0.099	16.698	0.081	0.102	NA	-0.006	0.299	TRUE	< 0.001
Poultry intake	0.095	0.758	1.123	0.570	0.720	NA	0.039	0.496	TRUE	< 0.001
Processed meat intake	23.716	0.096	25.940	0.076	0.079	NA	-0.004	0.238	TRUE	< 0.001
Salad / raw vegetable intake	19.623	0.075	19.966	0.096	0.174	NA	-0.002	0.655	TRUE	< 0.001
Salt added to food	102.093	0.100	102.095	0.113	0.128	NA	0.000	0.971	TRUE	< 0.001
Water intake	44.216	0.074	44.228	0.092	0.082	NA	0.000	0.926	TRUE	< 0.001
Hot drink temperature	69.332	0.093	75.291	0.044	0.050	NA	-0.003	0.034	TRUE	< 0.001
Tea intake	40.311	0.099	40.752	0.113	0.134	NA	0.001	0.571	TRUE	< 0.001
Alcohol intake frequency	10.076	0.688	10.868	0.696	0.469	NA	0.003	0.390	TRUE	< 0.001

Table S25: Heterogeneity and pleiotropy test for association of dietary habits and chronic knee pain.

Exposure	Heterogeneity test (MR-Egger)		Heterogeneity test (IVW)		PRESSO Method		Pleiotropy test (MR-Egger intercept test)		Steiger directionality test	
	Q	*p*	Q	*p*	Global *p*	Distortion Test *p*	Egger Inter	Inter *p*	Correct Causal Direction	Steiger *p*
Beef intake	16.729	0.116	17.891	0.119	0.177	NA	0.003	0.401	TRUE	< 0.001
Bread intake	43.364	0.013	43.703	0.016	0.019	0.768	0.001	0.662	TRUE	< 0.001
Cereal intake	24.723	0.643	28.759	0.478	0.428	NA	0.003	0.054	TRUE	< 0.001
Cheese intake	45.379	0.414	45.772	0.440	0.417	NA	0.001	0.540	TRUE	< 0.001
Coffee intake	28.377	0.392	29.419	0.392	0.442	NA	0.002	0.328	TRUE	< 0.001
Cooked vegetable intake	3.740	0.928	5.481	0.857	0.846	NA	0.008	0.220	TRUE	< 0.001
Dried fruit intake	24.901	0.580	27.151	0.510	0.516	NA	-0.002	0.145	TRUE	< 0.001
Fresh fruit intake	46.255	0.142	51.615	0.069	0.091	NA	-0.003	0.045	TRUE	< 0.001
Lamb/mutton	35.465	0.127	35.609	0.153	0.149	NA	-0.001	0.743	TRUE	< 0.001
Non-oily fish intake	3.745	0.442	3.790	0.580	0.615	NA	0.001	0.842	TRUE	< 0.001
Oily fish intake	72.368	0.013	73.088	0.014	0.015	0.408	-0.001	0.493	TRUE	< 0.001
Pork intake	8.962	0.441	9.146	0.518	0.540	NA	-0.002	0.678	TRUE	< 0.001
Poultry intake	5.228	0.022	5.667	0.059	0.134	NA	-0.025	0.820	TRUE	< 0.001
Processed meat intake	18.490	0.296	18.642	0.349	0.361	NA	0.001	0.722	TRUE	< 0.001
Salad / raw vegetable intake	22.487	0.032	22.526	0.048	0.107	NA	0.001	0.888	TRUE	< 0.001
Salt added to food	82.532	0.556	85.359	0.499	0.437	NA	0.001	0.096	TRUE	< 0.001
Water intake	30.229	0.556	31.467	0.543	0.548	NA	0.002	0.274	TRUE	< 0.001
Hot drink temperature	47.816	0.743	49.903	0.704	0.713	NA	-0.002	0.154	TRUE	< 0.001
Tea intake	30.344	0.448	33.215	0.360	0.327	NA	0.002	0.102	TRUE	< 0.001
Alcohol intake frequency	15.041	0.305	15.070	0.373	0.386	NA	-0.001	0.877	TRUE	< 0.001

Table S26: Heterogeneity and pleiotropy test for association of dietary habits and chronic headache.

Exposure	Heterogeneity test (MR-Egger)		Heterogeneity test (IVW)		PRESSO Method		Pleiotropy test (MR-Egger intercept test)		Steiger directionality test	
	Q	*p*	Q	*p*	Global *p*	Distortion Test *p*	Egger Inter	Inter *p*	Correct Causal Direction	Steiger *p*
Beef intake	26.002	0.006	26.014	0.011	0.014	**0.0381**	0.000	0.944	TRUE	< 0.001
Bread intake	31.056	0.187	33.813	0.140	0.097	NA	0.003	0.149	TRUE	< 0.001
Cereal intake	37.615	0.106	40.613	0.074	0.062	NA	0.004	0.146	TRUE	< 0.001
Cheese intake	48.400	0.300	48.879	0.320	0.351	NA	0.001	0.513	TRUE	< 0.001
Coffee intake	48.589	0.007	48.765	0.009	0.012	NA	0.001	0.757	TRUE	< 0.001
Cooked vegetable intake	17.547	0.041	18.305	0.050	0.064	NA	0.007	0.548	TRUE	< 0.001
Dried fruit intake	35.957	0.116	41.137	0.052	0.052	NA	0.005	0.059	TRUE	< 0.001
Fresh fruit intake	68.481	0.001	68.492	0.002	0.001	0.1324	0.000	0.939	TRUE	< 0.001
Lamb/mutton intake	33.182	0.191	33.453	0.219	0.243	NA	-0.001	0.643	TRUE	< 0.001
Non-oily fish intake	11.475	0.022	11.641	0.040	0.065	NA	0.002	0.822	TRUE	< 0.001
Oily fish intake	65.230	0.049	65.318	0.059	0.069	NA	0.000	0.800	TRUE	< 0.001
Pork intake	18.299	0.032	30.638	NA	0.001	0.3083	-0.018	0.036	TRUE	< 0.001
Poultry intake	0.188	0.665	3.490	0.175	0.230	NA	-0.085	0.320	TRUE	< 0.001
Processed meat	16.879	0.393	24.291	0.112	0.114	NA	-0.008	0.017	TRUE	< 0.001
Salad / raw vegetable intake	3.990	0.984	6.830	0.911	0.899	NA	-0.006	0.118	TRUE	< 0.001
Salt added to food	#######	0.003	130.825	0.001	0.001	NA	0.002	0.076	TRUE	< 0.001
Water intake	58.156	0.003	58.853	0.004	0.004	0.3365	-0.002	0.540	TRUE	< 0.001
Hot drink	83.003	0.009	83.877	0.009	0.018	NA	0.002	0.450	TRUE	< 0.001
Tea intake	38.916	0.128	39.673	0.137	0.175	NA	0.002	0.451	TRUE	< 0.001
Alcohol intake frequency	23.696	0.034	24.123	0.044	0.066	NA	0.003	0.637	TRUE	< 0.001
